# Metal‐Enhanced Charge Transport and its Mechanism in Atomically Precise Ruthenium Single‐Molecule Devices

**DOI:** 10.1002/advs.202520951

**Published:** 2026-04-20

**Authors:** Jie Guo, Qinghua Gao, Ping Duan, Cong Zhao, Huimin Wen, Xiaoyan He, Lucie Norel, Yuzhe Zhang, Chunwen Wang, Wu Zhou, Xinyue Chang, Ju Wang, Stéphane Rigaut, Mingliang Li, Chuancheng Jia, Xuefeng Guo

**Affiliations:** ^1^ Center of Single‐Molecule Sciences Institute of Modern Optics Frontiers Science Center For New Organic Matter Tianjin Key Laboratory of Micro‐Scale Optical Information Science and Technology College of Electronic Information and Optical Engineering Nankai University Tianjin P. R. China; ^2^ School of Integrated Circuits Shanghai Jiao Tong University Shanghai P. R. China; ^3^ Univ Rennes CNRS ISCR (Institut Des Sciences Chimiques de Rennes)‐UMR 6226 Rennes France; ^4^ School of Physical Sciences and CAS Key Laboratory of Vacuum Physics University of Chinese Academy of Sciences Beijing P. R. China; ^5^ School of Materials Science and Engineering Beijing Institute of Technology Beijing P. R. China; ^6^ Beijing National Laboratory for Molecular Sciences National Biomedical Imaging Center College of Chemistry and Molecular Engineering Peking University Beijing P. R. China

**Keywords:** charge transport, edge‐selective oxidation, hydrogen plasma etching, organometallic ruthenium molecules, single‐molecule devices

## Abstract

Achieving reliable, quantitative investigation of charge transport at the single‐molecule level remains a key challenge in molecular electronics. In this study, we develop a universal strategy to fabricate molecular devices based on edge‐selective chemical oxidation of graphene electrodes with atomically defined zigzag edges and controllable nanogaps, thus enabling precise covalent connection of molecules for device construction. Stable single‐molecule devices are then successfully created by covalently connecting three representative wire‐like organometallic ruthenium molecules (**Ru 1**, **Ru 2**, and **Ru 3**) between nanogapped graphene electrodes via an amidation reaction. The superior accuracy of our approach is substantiated by the exceptional device‐to‐device uniformity across various devices, with normalized standard deviations of ∼1.04%, ∼1.27%, and ∼0.91% for **Ru 1**, **Ru 2**, and **Ru 3**, respectively, which significantly surpass conventional methods. Leveraging this platform, we uncover a metal‐enhanced conductance effect characterized by an ultralow attenuation (*β*  =  0.069 nm^−^^1^), arising from strong electrode‐molecule covalent coupling. Furthermore, temperature‐dependent transport measurements reveal a characteristic barrier‐lowering mechanism that modulates charge injection. By enabling accurate investigation of intrinsic molecular transport properties, this study establishes a reproducible and precise experimental platform for developing future functional molecular devices.

## Graphical abstract

An atomically precise platform was developed for investigating single‐molecule charge transport. This innovative platform enables the creation of highly uniform molecular devices using ruthenium‐based molecules. It reveals a significant enhancement in molecular conductance due to the metallic ruthenium center and a unique barrier‐lowering effect during charge transport, offering new insights into molecular electronics.

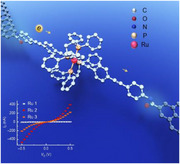

## 1 Introduction

Single‐molecule devices, in which an individual molecule serves as the key component within nanogapped electrodes, represent a frontier in exploring fundamental molecular properties and developing functional applications [1−6]. These devices not only address the miniaturization needs of conventional electronic devices but also enhance the performance of molecular nanocircuits [7−9]. At the single‐molecule scale, even subtle variations in the atomic arrangement or molecule/electrode interface structure can significantly affect the device performance, obscuring intrinsic molecular properties and compromising measurement reliability [9−13]. Therefore, the fabrication of highly precise, uniform, and stable single‐molecule devices becomes a prerequisite for achieving deterministic control over their electronic behavior.

Over decades of development, various methods have been devised for constructing single‐molecule devices with nanometer gaps, including static junctions [14−19] and dynamic junctions [20−25]. Static junctions are particularly well‐suited for creating stable devices that not only yield reliable molecular transport properties but also enable functional device applications. Traditional static junctions, such as electromigration junctions based on gold (Au) electrodes, are limited by the high atomic mobility and incompatibility with molecular sizes and energy levels. Carbon‐based materials, especially graphene, have emerged as promising electrode materials due to their low dimensionality, excellent atomic stiffness, and natural compatibility with organic molecules [26−32]. However, substantial challenges remain in precisely controlling the electrode gap and edge configuration, as well as in achieving an ideal molecule/electrode interface—both of which are essential for probing intrinsic charge transport.

Organometallic ruthenium (Ru) alkynyl molecular wires exhibit exceptional electronic properties due to the strong overlap between the *d*‐orbitals of the metal centers and the *π*‐systems of the alkynyl ligands, demonstrating significant potential for applications in molecular electronics and molecular devices [33]. To address this core challenge in probing intrinsic charge transport, we herein construct atomically precise single‐molecule devices and investigate charge transport in organometallic Ru complexes. To establish these devices, we first employ anisotropic hydrogen plasma etching to fabricate graphene electrodes with atomically precise gaps and zigzag edge configurations, leveraging the distinct reaction rates of zigzag and armchair carbon configurations. Subsequently, we utilize the enhanced reactivity of graphene edges for edge‐selective functionalization, introducing carboxyl groups to enable covalent linkage with amino‐terminated molecules. These functionalized electrodes are then covalently integrated through amidation reactions with a series of amino‐terminated organometallic Ru‐alkynyl wires (Schemes S1 and S2, Figures S1‒S10), which are selected to systematically investigate intrinsic charge transport properties by controlling variations in molecular length and the number of Ru centers. This approach enables the reliable construction of highly uniform single‐molecule devices, thereby allowing systematic investigation of charge transport in Ru‐alkynyl wires and, more importantly, revealing their intrinsic transport properties free from interfacial artifacts.

## 2 Results and Discussion

### 2.1 Fabrication of Atomically Precise Graphene Electrodes

**FIGURE 1:**
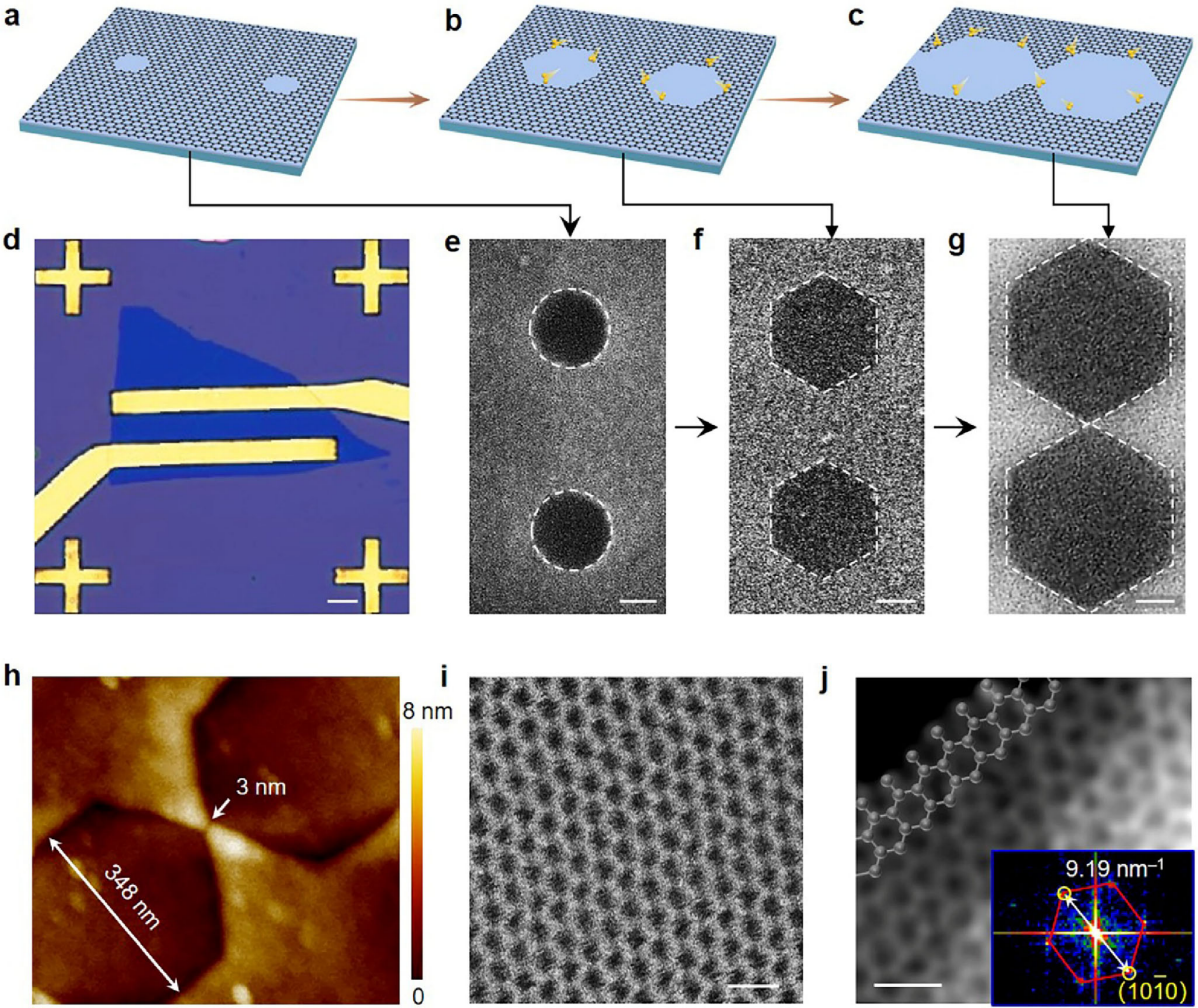
Atomically precise preparation of graphene triangular point electrodes. (a–c) Schematic representation of the formation process of graphene triangular point electrodes with zigzag edges and a controllable gap. (d) Optical image of a graphene sample. Scale bar, 20 µm. (e–g) SEM images of the entire etching evolution process. Scale bars, 100 nm. (h) AFM image of a graphene triangular electrode pair. (i,j) Atomically resolved STEM images of the graphene‐electrode basal plane (i) and edge (j). The Inset shows FFT analysis of the STEM image to determine the zigzag direction. Scale bars, 0.5 nm.

To establish a reliable platform for single‐molecule measurements, we fabricated graphene electrodes using anisotropic etching in a remote hydrogen plasma environment (Figure S11) [32, 34]. The fabrication of atomically precise graphene electrodes is illustrated in Figure 1a‒c and Figure S12. Starting with three‐layer graphene verified by optical microscopy contrast analysis (Figure S13), we integrated the metal electrodes for stepwise conductance monitoring (Figure 1d; Figure S14). A systematic etching approach (Figures S15‒S17) transformed initial circular hole arrays into hexagonal patterns, ultimately yielding triangular point electrodes with well‐defined nanogaps (Figure S18). This process is carefully monitored using scanning electron microscopy (SEM) (Figure 1e‒g) and stepwise current measurements (Figure S19), supported by theoretical modeling [35] to ensure precise gap control (Figures S20 and S21). The resulting electrode architecture features controllable gap dimensions and well‐defined edge configurations, which are essential for accurate molecular property characterization.

The atomic precision of our graphene electrodes is rigorously validated through multi‐modal microscopy characterization. The atomic force microscopy (AFM) is employed to determine the triangular graphene electrodes featuring controllable nanometer gap. An instance of an electrode pair has been included with an approximate gap size of 3 nm and a smooth surface with a transverse spanning width of 348 nm at its maximum etching extent in Figure 1h. Transmission electron microscopy (TEM) is used to validate the lattice structure and edge configuration of the graphene electrodes, with the sample preparation process for TEM elaborated in Section S1 and Figure S22. High‐resolution transmission electron microscopy (HR‐TEM) captures the edge‐smooth hexagonal morphology of the etched graphene, as shown in Figure S23. In addition, atomically resolved scanning transmission electron microscopy (STEM) is utilized for an in‐depth characterization of the graphene sample. The STEM image clearly shows the hexagonal grid with a C‒C bond length of approximately 0.14 nm (Figure 1i), as well as the atomically regular zigzag configuration of the graphene edge (Figure 1j) [36]. The fast Fourier transform (FFT) analysis incorporated in Figure 1j suggests that the six diffraction points correspond to the {$10\bar{1}0$} planes, and the link between the diffraction points ($10\bar{1}0$) and the central spot is perpendicular to the real‐space zigzag direction [37]. This evidence allows us to confirm the zigzag configuration of the graphene electrode's edge. This clear lattice structure and edge configuration form an important technical foundation for the construction of highly uniform single‐molecule devices at the atomic level.

### 2.2 Edge‐Selective Modification of Graphene Electrodes

**FIGURE 2:**
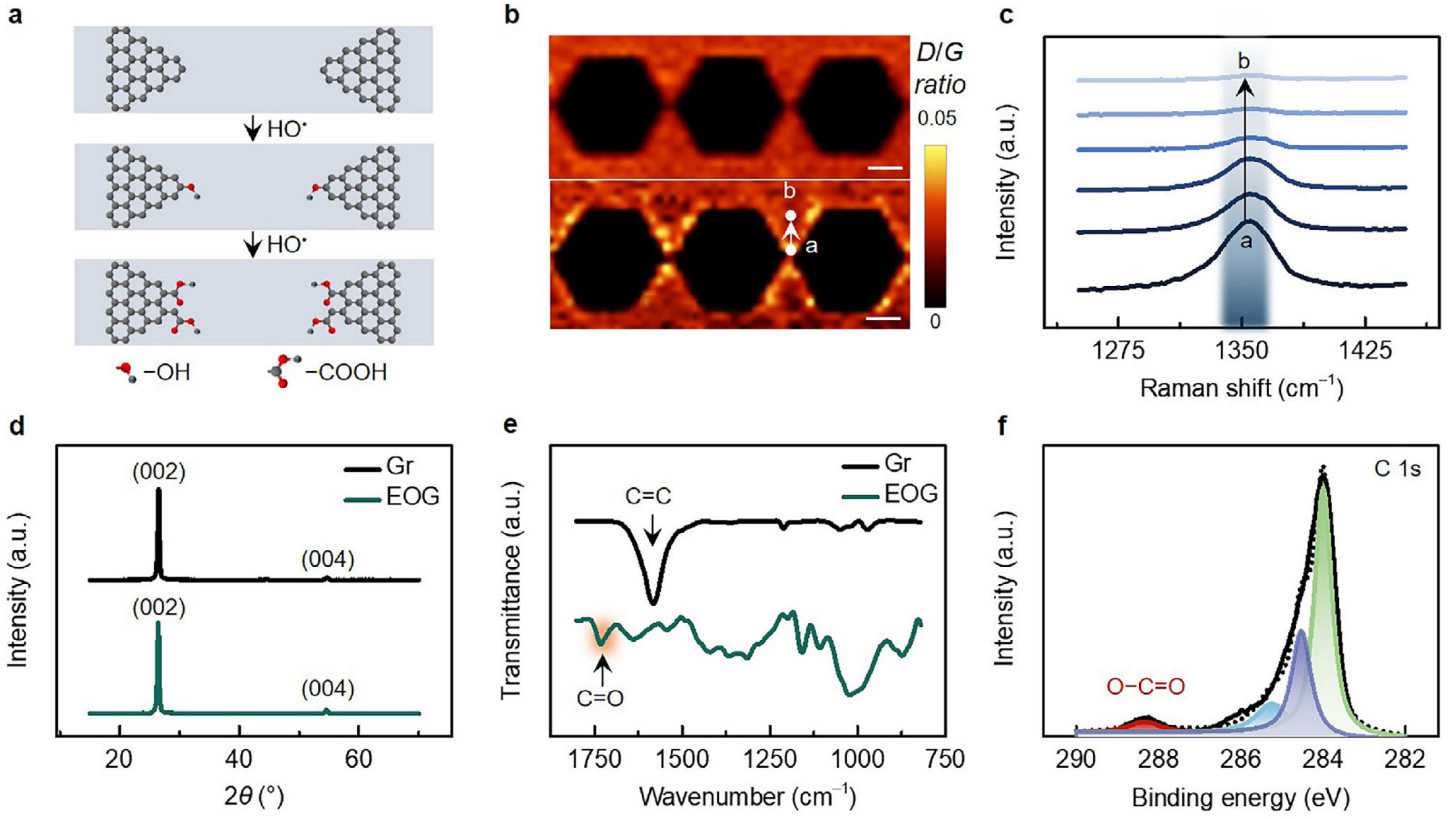
Controllable edge‐selective modification of graphene triangular electrodes. (a) Schematic diagram of edge‐selective oxidation of graphene triangular electrodes. (b) Raman mapping of the *D*/*G* intensity ratio for the hole array of graphene before (upper panel) and after (lower panel) edge‐selective oxidation. Scale bars, 1 µm. (c) Defect peak intensity of single‐point Raman at different sites (from point “a” to “b”) corresponding to the graphene sample. (d) XRD patterns of graphene (black curve) and edge‐oxidized graphene nanoflakes (green curve). (e) FTIR spectra of graphene nanoflakes before (black curve) and after edge‐selective oxidation (green curve). (f) High‐resolution C 1s XPS spectrum of edge‐oxidized graphene nanoflakes.

The edge of graphene triangular electrodes surrounded by hydrogen atoms significantly limits the construction of the molecule/electrode interface. A novel method that employs a mild and controllable oxidation process to selectively adapt the carboxyl functional groups to the edges of graphene electrodes, thereby promoting covalent binding with amino‐terminal molecules (Figure 2a). The process starts with the decomposition of hydrogen peroxide (H_2_O_2_) into hydroxyl radicals (HO^•^) in the presence of concentrated sulfuric acid (H_2_SO_4_), which then oxidize the edge of the graphene, leading to the emergence of hydroxyl groups. Subsequently, the hydroxylated graphene reacts with HO^•^ radicals, resulting in the formation of carbonyl groups. Ultimately, the carbonyl groups at the edge of graphene further react with HO^•^, culminating in the production of edge carboxyl groups (Figure S24). This process does not inflict any damage on the graphene triangular electrodes (Figure S25).

Figure 2b provides a comparative analysis of Raman mapping, specifically focusing on the ratio of defect peak intensity to in‐plane vibration peak intensity (*D*/*G* ratio), for pristine graphene and edge‐selectively oxidized graphene (EOG). Sample preparation for Raman characterization is detailed in Section S1 and Figure S26. It is clearly observable that the defects generated by functionalization are predominantly concentrated at the edges of the regular hexagonal holes, while the basal plane exhibits negligible defect features. The *D*‐band intensities of single‐point Raman at different selected sites (from point “a” to point “b”) in the graphene samples, as illustrated in Figure 2b, show a noticeable difference. The *D*‐band intensity near point “a”, which signifies the edge, is markedly high. On the contrary, the *D*‐band intensity near point “b”, indicative of the basal plane, is considerably weak and can essentially be disregarded (Figure 2c; Figure S27). The specific extent of oxidation at both the basal plane and the edges of graphene can be further elucidated through the analysis of normalized Raman spectra, extracted from edge and center spots in the mappings (Figure S28). These findings suggest that the functionalization reaction occurs selectively at the edges of graphene.

Figure 2d presents a comparative analysis of the X‐ray diffraction (XRD) patterns pertaining to the (002) plane of graphene, both pre and post oxidation. The most intense peak corresponding to the (002) plane before oxidation is found at 2*θ* = 26.52° (black curve in Figure 2d). For post oxidation, however, this peak shifts to 2*θ* = 26.44° (green curve in Figure 2d). According to Bragg's Law, this shift signals an increase in the interlayer spacing of graphene from 0.3358 nm (pre oxidation) to 0.3368 nm (post oxidation), attributed to the emergence of carboxyl functional groups at the edges of graphene nanoflakes. Notably, the (002) plane continues to exhibit a strong peak intensity after oxidation, indicating that the crystallinity of the graphene nanoflakes remains unchanged after edge‐selective oxidation [38]. This oxidation process and resultant carboxylation are substantiated by the detection of C═O stretching vibration peak at 1720 cm^−1^ in the Fourier transform infrared spectra (FTIR) (Figure 2e), an amplified presence of oxygen‐containing groups in the survey X‐ray photoelectron spectra (XPS) (Figure S29), and the detection of O─C═O species (∼288.4 eV) in the high‐resolution C 1s XPS spectrum (Figure 2f; Figure S30).

### 2.3 Conductance Characteristics of Ru Single‐Molecule Devices

**FIGURE 3:**
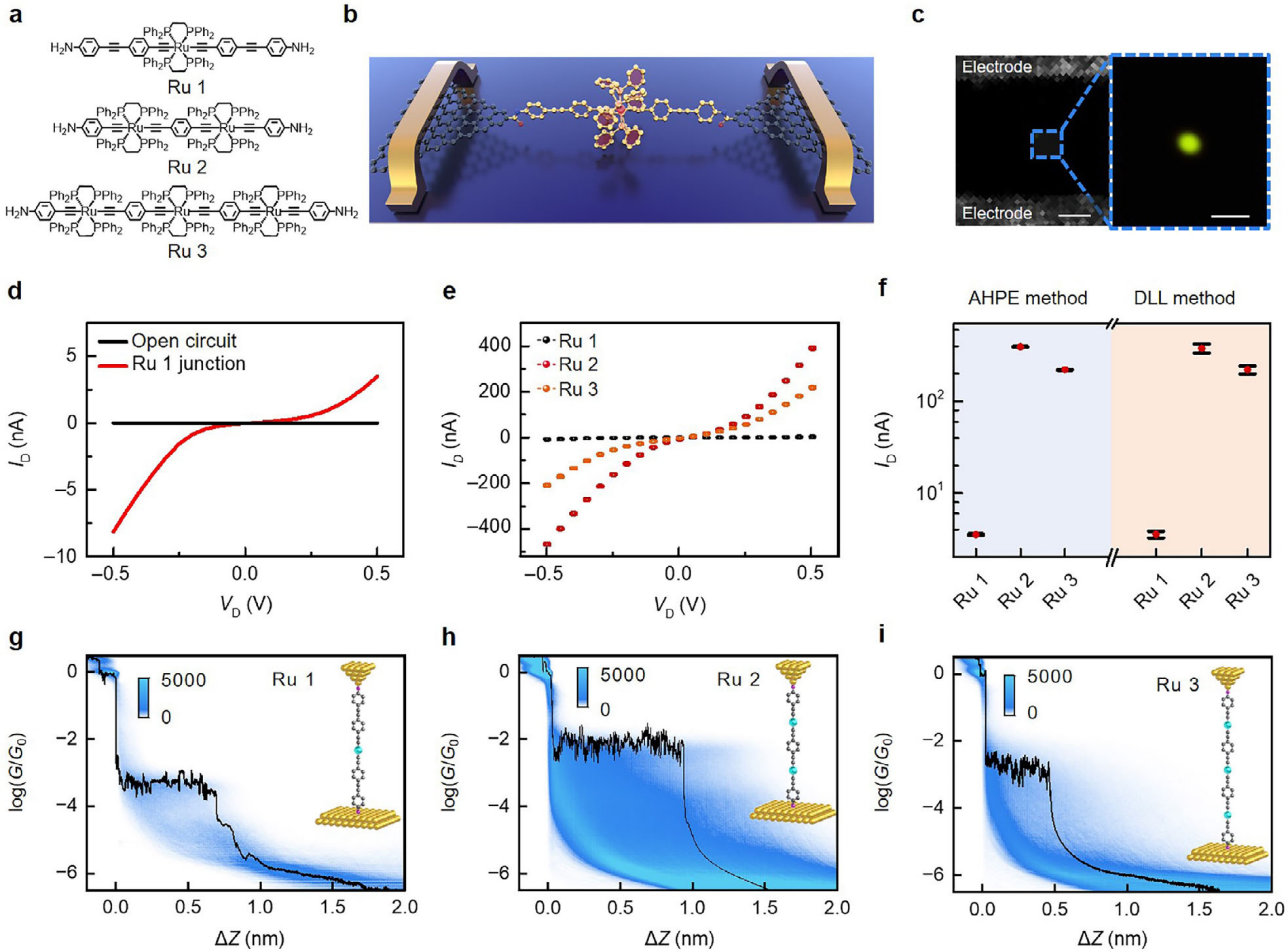
Conductance characteristics of Ru single‐molecule devices. (a) Molecular structures of **Ru 1**, **Ru 2**, and **Ru 3**. (b) Schematic representation of a single‐molecule device. (c) Ultrahigh‐resolution images of a single‐**Ru‐1**‐connected device obtained by the STORM technology. Scale bars, 500 nm (main); 50 nm (inset). (d) *I*_D_–*V*_D_ curves of open circuit (black curve) and single‐molecule device after molecular connection (red curve) of **Ru 1**. (e) *I*_D_–*V*_D_ curves of three organometallic Ru single‐molecule devices. The small dots denote the data means of 5 devices, and the error bars indicate standard deviations (SDs) from the mean values. (f) Current distributions of **Ru 1**, **Ru 2**, and **Ru 3** devices using AHPE and DLL methods under the bias voltage of 0.5 V. The small red dots denote the data means based on 5 devices, and the error bars indicate SDs from the mean values. (g–i) 2D conductance‐displacement histograms of **Ru 1**, **Ru 2**, and **Ru 3** STM‐BJs.

An amidation reaction is employed to link three organometallic Ru molecules (**Ru 1**‒**Ru 3**, as shown in Figure 3a) with graphene electrodes based on the method reported in previous works [28, 29]. This approach facilitates the construction of stable single‐molecule devices (Figure 3b), achieving device yields of 72%, 70%, and 76% for **Ru 1**, **Ru 2**, and **Ru 3**, respectively. These organometallic Ru molecules are fluorescent with excitation and emission wavelengths ranging from 361 to 384 nm and 433 to 458 nm, respectively (Figures S31‒S33). To measure the targeted positions, we adopt stochastic optical reconstruction microscopy (STORM) [39]. The visualization of a bright fluorescent spot between electrodes substantiates the successful connection of a single organometallic Ru molecule (Figure 3c; Figure S34). Furthermore, the successful integration of the three organometallic Ru molecules can be further characterized by analyzing the current‐voltage (*I*_D_–*V*_D_) curves, which show a distinct response in comparison with the flat line noted prior to the connection (Figure 3d; Figure S35). Furthermore, robust covalent anchoring endows these junctions with high operational stability, with representative devices remaining conductive up to ∼3.8 V before bond rupture or irreversible electrode damage (Figure S36).

The uniformity of the device‐to‐device performance is an essential prerequisite for the investigation of the properties of single‐molecule devices. Here, two types of electrode configurations, namely, precise and traditional configurations have been fabricated by the anisotropic hydrogen plasma etching (AHPE) method and the previously described dash‐line lithography (DLL) method as a control (Figure S37) [19], with the aim of examining their respective effects on single‐molecule devices. In the case of precise configuration, a relatively concentrated distribution of conductance curves can be observed (Figure 3e; Figures S38‒40). In contrast, the traditional configuration exhibits a scattered distribution of conductance curves (Figures S41‒S47). Figure 3f illustrates the current distributions of **Ru 1**, **Ru 2**, and **Ru 3** at 0.5 V. In devices with traditional configurations, the currents through **Ru 1**, **Ru 2**, and **Ru 3** show greater variation, while concentrated currents are observed in devices with precise configurations.

Conductance characteristics of molecules are closely related to their lengths and configurations. Gauss simulations reveal that the lengths of **Ru 1**, **Ru 2**, and **Ru 3** complexes measure ∼3.166, ∼3.024, and ∼4.254 nm, respectively (Figure S48). Conductance measurements at 0.1 V, conducted across five different devices, yield average conductance values of approximately 1.24, 196.65, and 180.62 nS for **Ru 1**, **Ru 2** and **Ru 3**, respectively (Figure 3e). According to the formula *G* = *G*_0_exp (−*βL*), the devices incorporating **Ru 2** and **Ru 3** exhibit an extraordinarily low attenuation factor (*β*) of 0.069 nm^‒1^ (Figure S49), a value significantly lower than that observed in typical conjugated wire‐like molecules [40]. This exceptional attenuation minimization in the organometallic Ru molecules suggests strong electronic coupling between the Ru centers and the carbon‐rich ligands. In addition, despite the similar molecular lengths of **Ru 1** and **Ru 2**, the conductance of **Ru 2** significantly surpasses that of **Ru 1**. This disparity in conductivity can be attributed to the molecular configuration, specifically the quantity of embedded organometallic Ru units: **Ru 1** contains one unit, while **Ru 2** encompasses two units. These findings indicate that the inclusion of additional organometallic Ru units can effectively enhance molecular conductance.

To verify the reliability of the molecular properties obtained through the precise graphene electrode configuration, Au electrodes are employed as a reference system for conductance comparison across the three complexes. Single‐molecule conductance measurements are executed, utilizing the scanning tunneling microscopy break junction (STM‐BJ) technique with these Au electrodes [25]. As the Au tip rises vertically from the Au substrate, only a single molecule, equipped with two amine anchor groups on each side, can simultaneously self‐assemble to both the tip and the substrate, thereby forming a single‐molecule junction for conductance measurement. The conductance‐displacement histograms for **Ru 1**, **Ru 2**, and **Ru 3** are presented in both 2D (Figure 3g‒i) and 1D (Figure S50), showing thousands of unfiltered traces. The special peak at 10^0^ *G*_0_ in each figure corresponds to the Au‒Au point contacts, with the unit *G*_0_ = 2*e* ^2^/*h* ≈ 77.5 µS representing the conductance quantum, and other conductance peaks below 10^0^ *G*_0_ corresponding to the bonded molecules [41]. For the **Ru 1** complex, a conductance peak appears around ≈10^−5.0^ *G*_0_ (≈0.775 nS) at 0.1 V. In contrast, the **Ru 2** complex shows a higher conductance peak at ≈10^−2.5^ *G*_0_ (≈245.08 nS), while **Ru 3** exhibits a peak near ≈10^−2.9^ *G*_0_ (≈97.57 nS). Therefore, the conductance of the **Ru 2** complex is the highest, while that of **Ru 1** is the lowest, which aligns with the results obtained from graphene electrodes. Note that **Ru 2** shows a longer stretching plateau than **Ru 1** and **Ru 3** (Figure 3g–i). This difference reflects distinct molecule‐substrate interactions: the Ph−C≡C−Ph moiety in **Ru 1** promotes strong Au–*π* interactions that facilitate early bond rupture, while the greater number of PPh_2_ ligands in **Ru 3** enhances substrate binding, limiting its stretchability relative to **Ru 2**. The conductance attenuation of **Ru 2** and **Ru 3** STM‐BJs gives a *β* value of 0.76 nm^‒1^ (Figure S51), which surpasses the decay constant measured by graphene electrodes. The reason why the conductance attenuation of graphene‐based devices is lower than that of STM‐BJs can be attributed to the differences in electrode‐molecule coupling. Furthermore, the normalized standard deviations (SDs) of **Ru 1**, **Ru 2**, and **Ru 3** measured in devices using the AHPE method consistently fall below those of devices employing the DLL method and STM‐BJ method (Figures S52 and S53). For instance, the normalized SDs for **Ru 1**, **Ru 2**, and **Ru 3** devices using the AHPE method recorded at 0.1 V are 1.04%, 1.27%, and 0.91%, respectively, outperforming other systems (as shown in Figure S53: 1.45%, 3.78%, and 2.16% for the DLL method; 37.20%, 37.19%, and 37.18% for the STM‐BJ method). This indicates that reliable device‐to‐device uniformity in single‐molecule devices has been successfully achieved through the AHPE method.

### 2.4 Temperature‐Dependent Charge Transport Mechanism

**FIGURE 4:**
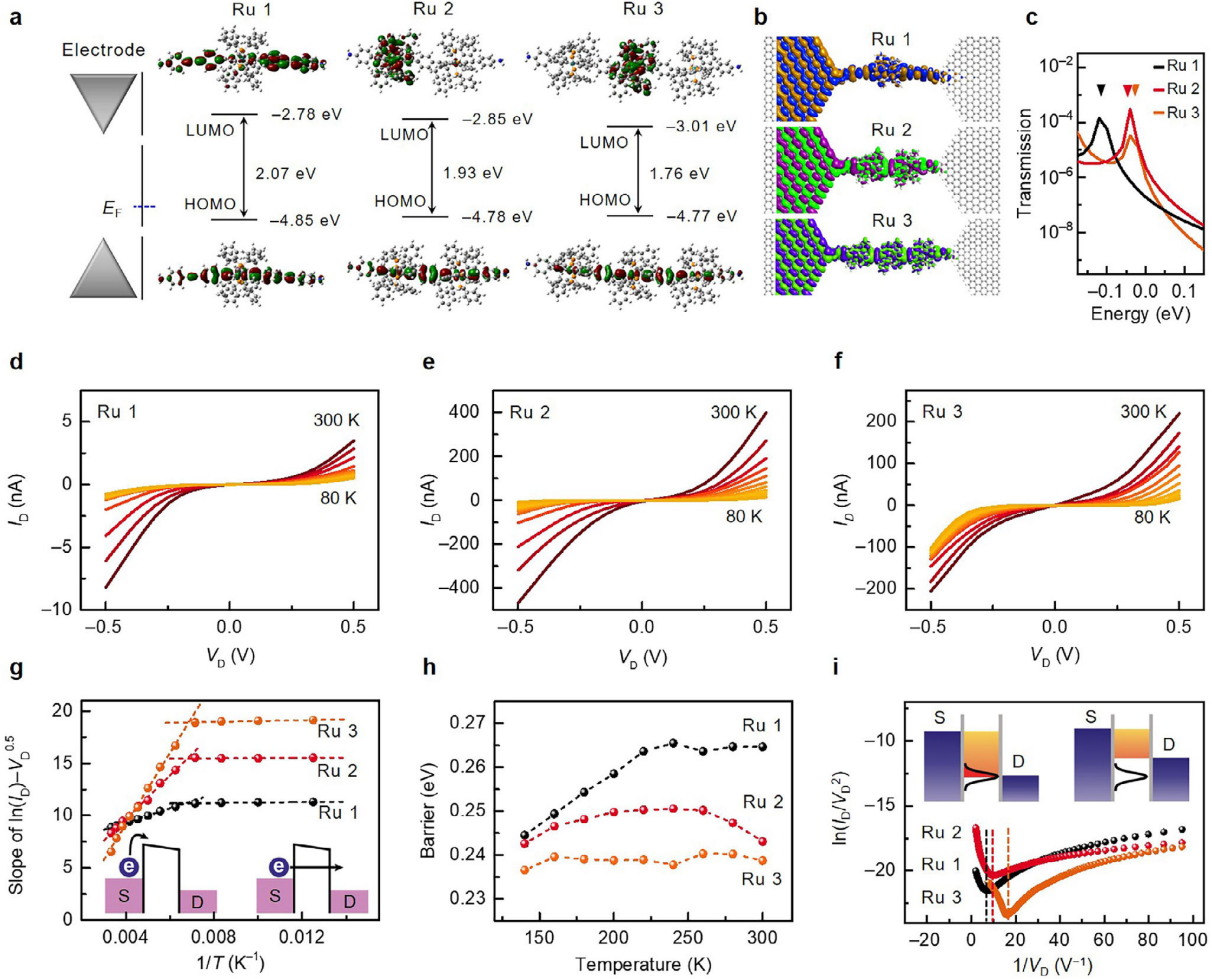
Temperature‐dependent charge transport characteristics of Ru single‐molecule devices. (a) The energy levels and corresponding molecular orbital diagrams of **Ru 1**, **Ru 2**, and **Ru 3** devices. The Fermi level of graphene is −4.6 eV. (b) Scattering states of the *p*‐HOMOs of **Ru 1**, **Ru 2**, and **Ru 3** devices. (c) Theoretical transmission spectra of **Ru 1**, **Ru 2**, and **Ru 3** devices. (d–f) Temperature‐dependent *I*_D_‒*V*_D_ curves of **Ru 1**, **Ru 2**, and **Ru 3** devices. (g) Plots of the slopes of (ln(*I*_D_) vs. *V*_D_^0.5^) vs. 1/*T* of **Ru 1**, **Ru 2**, and **Ru 3** devices. Insets show the barrier models at different temperatures. (h) Thermal emission barriers of **Ru 1**, **Ru 2**, and **Ru 3** at different temperatures. (i) Plots of ln(*I*_D_/*V*_D_^2^) vs. 1/*V*_D_ of **Ru 1**, **Ru 2**, and **Ru 3** devices. Insets show the charge transport models at different biases.

According to the molecular orbital diagrams, supplemented by charge scattering states for **Ru 1**, **Ru 2**, and **Ru 3** devices and molecular projected self‐consistent Hamiltonian (MPSH) analysis, it is clear that the highest occupied molecular orbitals (HOMO) predominantly drive the transmission in all three organometallic Ru electronic devices (Figure 4a,b; Figure S54). Furthermore, the theoretical simulations of transmission spectra for **Ru 1**, **Ru 2**, and **Ru 3** devices, as well as STM‐BJs, suggest that the perturbed highest occupied molecular orbitals (*p*‐HOMOs) are located close to the electrode Fermi level (Figure 4c; Figure S55), which implies that *p*‐HOMOs dominate charge transport. Specifically, the transmission coefficient near the Fermi level for the **Ru 2** devices is slightly higher than that of **Ru 3** and significantly higher than that of **Ru 1**. Consequently, **Ru 2** exhibits superior conductivity in comparison with both **Ru 3** and **Ru 1**, a trend that aligns with the *I*_D_‒*V*_D_ measurements. When plotting the distances between *p*‐HOMO and the electrode Fermi level (*E*_Fermi_ – *E_p_*_‐HOMO_) for **Ru 1**, **Ru 2**, and **Ru 3** molecules extracted from transmission spectra, it can be observed that the decay trend in the graphene‐based devices is less than that in STM‐BJs, resulting in a lower exponential decay (Figure S56).

Temperature‐dependent measurements are conducted to elucidate the inherent charge transport mechanism based on the temperature‐dependent *I*_D_–*V*_D_ characteristics. The temperature‐dependent *I*_D_–*V*_D_ curves are recorded from 80 to 300 K under high vacuum in the dark, within a bias range of ±1 V. As illustrated in Figure 4d‒f, an increase in current is observed for all three single‐molecule devices as the temperature ascended from 80 to 300 K. This implies the existence of a thermally activated transport mechanism, such as thermionic emission or hopping conduction [42]. However, the hopping mechanism can be dismissed based on the temperature‐dependent *I*_D_‒*V*_D_ curves, given that ln(*I*_D_/*V*_D_) exhibits a nonlinear relationship with 1/*T* (Figure S57) [43, 44]. In contrast, linear characteristics are discernible in the slope of (ln(*I*_D_) vs. *V*_D_^0.5^) vs 1/*T* (Figure 4g), as well as in the plots of ln(*I*_D_) vs *V*_D_^0.5^ (Figure S58), indicating the characteristics of the thermionic emission mechanism. By plotting the slope of (ln(*I*_D_) vs. *V*_D_^0.5^) as a function of 1/*T*, we can identify the inflection points for **Ru 1**, **Ru 2**, and **Ru 3** devices at 149, 147, and 146 K, respectively, suggesting a transition in the charge transport mechanism. At higher temperatures, the behavior of the three organometallic Ru molecules aligns well with thermionic emission mechanism, which can be explained by the following relationship from the Richardson‐Schottky (RS) model [45]:

(1) $$I_{D}=AT^{2}\exp\left(-\frac{q\Phi -q\sqrt{\frac{qV_{D}}{4\pi \varepsilon_{0}\varepsilon d}}}{kT}\right)$$

where *Φ* represents the height of the thermal emission barrier, *q* is the electron charge, *ε_0_* is the vacuum dielectric constant, *ε* is the relative dielectric constant, *d* is the distance between the graphene electrodes, and *k* is the Boltzmann constant. Here, *A* is the effective Richardson constant, which can be determined from the *y*‐axis intercept of the logarithmic plot of *I*_D_/*T^2^* vs. 1/*T* (Figure S59) [46]. Using this *A* value, the Schottky barrier can be calculated from Equation (1), as summarized in Figure 4h. The calculated thermal emission barrier decreases sequentially from **Ru 1** to **Ru 2** and then to **Ru 3**, suggesting that the presence of additional metal centers curtails the increase in the barrier with temperature, potentially influenced by the spatial profile of the electrostatic potential (Figure S60). The thermal emission barrier can be typically reflected from the difference between the graphene‐electrode Fermi level and the adjacent conducting molecular orbital energy level. The energy gap extracted from Figure 4c approximately corresponds to the thermal emission barrier [47].

At low temperatures, the dependence of charge transport on temperature is weak, and tunneling emerges as the dominant transition mechanism (Figure 4i). Figure 4i presents the plots of ln(*I*_D_/*V*_D_^2^) vs. 1/*V*_D_ for **Ru 1**, **Ru 2**, and **Ru 3** as specified by Equation (2) [48]:

(2) $$I_{D}\propto {V_{D}}^{2}\exp\left(-\frac{4d\sqrt{2m}\Phi^{3/2}}{3q\hbar V_{D}}\right)$$

The linear decrement at the high bias is in accordance with the Fowler‐Nordheim (F‐N) tunneling regime predicted by Equation (2). The dashed line denotes the transition voltage (*V*_trans_) required for the transition from direct tunneling to F‐N tunneling, which aligns with the energy gap from the molecular orbital diagram. The arrangement of the proximity between *p*‐HOMO and the electrode Fermi level follows the order **Ru 3**, **Ru 2**, and **Ru 1** (Figure 4c). Therefore, the *p*‐HOMO peak of the **Ru 3** device first enters the bias window, resulting in a lower transition voltage [49].

## 3 Conclusion

In conclusion, we have established a robust and atomically precise platform for reliable investigation of single‐molecule charge transport properties. Precise engineering of the fabrication process has enabled the construction of graphene triangular point electrodes featuring well‐defined zigzag edges and controllable nanogaps at the atomic level. The edges of graphene electrodes are precisely functionalized by non‐destructive oxidation, which is crucial for subsequent molecular assembly. The exceptional uniformity and stability of the constructed single‐molecule devices have been confirmed through covalent connection with three organometallic Ru molecules, yielding unprecedented device‐to‐device consistency. Electrical characterization revealed a strong conductance enhancement effect from the Ru center, along with an exceptionally low attenuation coefficient that is significantly smaller than values obtained using Au electrodes. This remarkable reduction in attenuation stems from the strong coupling achieved through covalent bonding at the molecule/electrode interfaces. Temperature‐dependent studies further uncovered a barrier‐lowering mechanism in charge transport. These results provide fundamental insights into charge transport through metal‐organic molecular wires and establish a reliable experimental platform that lays the groundwork for rational design of high‐performance molecular electronic devices.

## Author Contributions

X.G., C.J. and M.L. conceived the idea for the paper. J.G., Q.G., C.Z. and J.W. carried out experimental measurements. P.D. built and analyzed the theoretical model and performed the transport calculation. S.R., L.N., X.He. and H.W. synthesized the molecules. C.W. and Z.W. carried out the STEM. X.G., C.J., M.L., J.G., Q.G., P.D. and C.Z. analyzed the data and wrote the paper. All the authors discussed the results and commented on the manuscript.

## Conflicts of Interest

The authors declare no conflicts of interest.

## Supporting information




**Supporting File**: advs75401‐sup‐0001‐SuppMat.pdf.

## Data Availability

The data that support the findings of this study are available from the corresponding author upon reasonable request.
