# Supplementary material for: Metal‐Enhanced Charge Transport and its Mechanism in Atomically Precise Ruthenium Single‐Molecule Devices
Source: Adv Sci (Weinh). 2026 Apr 20;13(40):e20951. doi: 10.1002/advs.202520951 (PMC13335432; doi:10.1002/advs.202520951)
Supplement: Supplementary file 1 — Supporting File: advs75401‐sup‐0001‐SuppMat.pdf. [file ADVS-13-e20951-s001.pdf]

Supplementary Information

Metal-Enhanced Charge Transport and its Mechanism in Atomically Precise Ruthenium Single-Molecule Devices

Jie Guo[1,†], Qinghua Gao[1,†], Ping Duan[1,†], Cong Zhao[1,†], Huimin Wen[2], Xiaoyan He[3], Lucie Norel[3], Yuzhe Zhang[1], Chunwen Wang[4], Wu Zhou[4], Xinyue Chang[1], Ju Wang[1], Stéphane Rigaut[3*], Mingliang Li[5*], Chuancheng Jia[1*] and Xuefeng Guo[1,6*]

[1]Center of Single-Molecule Sciences, Institute of Modern Optics, Frontiers Science Center for New Organic Matter, Tianjin Key Laboratory of Micro-Scale Optical Information Science and Technology, College of Electronic Information and Optical Engineering, Nankai University, 38 Tongyan Road, Jinnan District, Tianjin 300350, P. R. China.

[2]School of Integrated Circuits, Shanghai Jiao Tong University, Shanghai 200240, P. R. China.

[3]Univ Rennes, CNRS, ISCR (Institut des Sciences Chimiques de Rennes)-UMR 6226, F-35000 Rennes, France.

[4]School of Physical Sciences and CAS Key Laboratory of Vacuum Physics, University of Chinese Academy of Sciences, Beijing 100049, P. R. China.

[5]School of Materials Science and Engineering, Beijing Institute of Technology, Beijing 100081, P. R. China.

[6]Beijing National Laboratory for Molecular Sciences, National Biomedical Imaging Center, College of Chemistry and Molecular Engineering, Peking University, 292 Chengfu Road, Haidian District, Beijing 100871, P. R. China.

*Corresponding authors. E-mail: guoxf@pku.edu.cn (X.G.); jiacc@nankai.edu.cn (C.J.); liml@bit.edu.cn (M.L.); stephane.rigaut@univ-rennes.fr (S.R.)

[†]These authors contributed equally to this work.

Table of Contents

1. General Methods

2. Fabrication of Atomically Precise Graphene Electrodes

3. Molecular Connection

4. Device Characteristics

5. Cartesian Coordinates of the Optimized Molecular Geometries

6. References

1. General Methods

1.1 Fabrication of Atomically Precise Graphene Electrodes: The SiO_2/Si wafer (300 nm SiO_2 layer) was soaked in piranha solution ($V_{H_2SO_4}$:$V_{H_2O_2}$ = 7:3) for 4 hours at 110 °C to remove surface impurities, followed by sonication 3 times in deionized water for cleaning. Mark the cleaned SiO_2/Si wafer with '+' characters by photolithography for the subsequent determination of the position of mechanically exfoliated graphene. Select a silicon wafer with clear mark patterns, and ultrasonically clean it with isopropanol (*i*-PrOH), acetone, water, and ethanol in sequence for about 10 minutes to remove inorganic and organic impurities adsorbed on the surface, and then by oxygen plasma for 6 minutes. Afterwards, press one sheet of Kish graphite (B type, Omtech Opto-electronics Co., Ltd.) to the center area of Scotch tape (3M), and repeatedly fold it in half to evenly distribute the Kish graphite sheet on the tape. Then, stick the tape to the SiO_2/Si substrate and tightly press it on the glass slide for about 4−5 hours, firmly adhere the SiO_2/Si substrate with graphene through van der Waals forces. After removing the Scotch tape from the SiO_2/Si substrate, locate and record the position of graphene sheet with the help of mark patterns under a standard optical microscope equipped with a charge-coupled device (CCD) camera.

Take photos of few-layered graphene under 20 × objective lens and correct the size and angle of the photos. Then, use Auto-CAD software to draw electrode patterns on graphene photos that have been corrected for size and angle and draw four additional '+' marks for further e-beam lithography (EBL) process. Next, two-layered poly(methyl) methacrylate (PMMA, MicroChem.) was spin-coated on the SiO_2/Si wafer. Firstly, coat 495K PMMA by 650 r/min for 3 s and then 4000 r/min for 45 s. Heat the wafers at 180 °C for 2 min. After cooling, repeat the above steps but use 950K PMMA. Then apply EBL (GeminiSEM 300 from Zeiss) to expose the drawn electrode area. After EBL, a solution mixed with *i*-PrOH and methyl isobutyl ketone (MIBK) ($V_{i\text{-PrOH}}$:V_{MIBK} = 3:1) was used to develop for 5 s and the *i*-PrOH solution was used to fix for 20 s. Place the wafer in a thermal resistance evaporation coater and evaporate 8 nm Cr (99.999% purity) and 80 nm Au (99.999% purity), and then place it in an e-beam evaporation coating machine (MB ProVAP from Mbraun) to e-beam evaporate with a 40 nm silicon dioxide

(SiO_2) protective layer. After evaporation, soak the wafer in acetone to peel PMMA off the wafer, obtaining a graphene device covered with Au electrodes.

Take photos of graphene devices under 100 × objective lens. Due to slight distortion during the shooting process, it is necessary to correct the size, angle, etc. of the photo using a self-developed program on MATLAB. Then import the corrected photo into Auto-CAD to draw reference rectangles and circular hole arrays (hole radius 0.1 μm, spacing 0.5 μm) used for pre-etching defect sites. The SiO_2/Si wafer was spin-coated with 950K PMMA and baked at 180 °C for 2 min. After that, the wafer was processed by EBL to expose the pre-etched patterns. A solution mixed with *i*-PrOH and MIBK ($V_{i\text{-PrOH}}$:V_{MIBK} = 3:1) was used to develop PMMA for 30 s, and then fix in *i*-PrOH for 20 s. At this time, PMMA on the exposed reference rectangular and circular hole areas will disappear. Use the reactive ion etching (RIE, Etchlab 200 from SENTECH) technology to etch the initial reaction site and set the oxygen gas flow rate to 50 sccm, etching power to 50 W, and etching time to 50 s. Afterwards, immerse the wafer in acetone to peel the PMMA film off the wafer. Then place the wafer with prefabricated circular holes in a laboratory-built plasma-enhanced hydrogen etching system to gradually etch hexagonal patterns on the initially fabricated circular holes. The pre-etching hexagonal patterns captured by SEM (GeminiSEM 300 from Zeiss) were further imported into Auto CAD, and an angle-measurement tool was selected to measure the angle of one edge of the hexagonal relative to the reference rectangle, that is, the angle of the zigzag-lattice direction of graphene.

Based on the zigzag-lattice direction of graphene determined by pre-etching, repeat the pre-etching steps and obtain circular hole array (hole radius 0.075 μm, spacing 0.4 μm) with the same direction as graphene zigzag lattice orientation through EBL and RIE between Au electrodes. Then place the wafer with circular hole array pattern in laboratory-built system based on plasma-enhanced hydrogen etching, with an etching temperature of 500 °C, an etching power of 8 W, and a hydrogen flow rate of 10 sccm. Graphene in the channel region is gradually etched away by the remote hydrogen plasma until a nanogap is formed.

1.2. TEM Sample Preparation: We used an *i*-PrOH-assisted direct-transfer method with hydrofluoric acid (HF) etching to characterize the edges of etched graphene by TEM. Firstly, with EBL and plasma-enhanced hydrogen etching of high-quality graphene scattered on the SiO_2/Si wafer obtained by mechanical exfoliation, we prepared hexagonal array patterns on the graphene. Place the TEM grid directly on the wafer and drip a drop of *i*-PrOH above the TEM grid. As *i*-PrOH evaporates, the adhesion between the TEM grid and graphene gradually tightens. After the complete evaporation of *i*-PrOH, HF was added dropwise on the SiO_2/Si wafer. The SiO_2 layer on the surface of the wafer loaded with graphene will be corroded by HF, and the TEM grid will detach from the substrate. At the same time, graphene will detach from the substrate and adsorb on the TEM grid. After soaking in deionized water and drying, the TEM grid stuck with graphene can be characterized using HR-TEM (JEM-2800, JEOL) and STEM. STEM experiments were performed on the monochromated Nion U-HERMES 100 microscope equipped with an alpha-type monochromator and a fifth-order aberration corrector at 60 kV.

1.3. Raman Mapping of Edge-Selectively Oxidized Graphene: Firstly, take pre-etching process to determine the zigzag-lattice orientation of mechanically exfoliated graphene. Due to the optical diffraction limit in Raman mapping resolution, we obtained larger initial circular holes (hole radius 0.75 μm, spacing 3.5 μm) on graphene with the same direction as the zigzag-lattice orientation through EBL and RIE, and on this basis, hexagonal holes were formed using plasma-enhanced hydrogen etching. The etching can be stopped until two hexagonal holes come into point-to-point contact, which was directly used for characterization of Raman mapping. Then place the SiO_2/Si wafer in a single bottle and perform the process of controllable edge-selective oxidation as described above. After oxidation, rinse the wafer with deionized water and blow dry it for the Raman characterization. Raman characterization was performed by alpha300RAS from WITec.

1.4. Characterization of Edge-Selectively Oxidized Graphene Nanoflakes: Using graphene nanoflakes (Cheap Tubes Inc.) as raw materials, edge-selectively oxidized graphene was synthesized by a controllable acid-oxidation method. Specifically, a mixture of graphene

nanoflakes (25 mg), deionized water (0.5 mL), and H_2O_2 (0.75 mL, 30%) was stirred for half an hour in an ice bath, and then 0.5 mL of H_2SO_4 was slowly added dropwise. After stirring in an ice water bath for 1 h, remove the ice bath and stir at room temperature for 4 h. Finally, the mixture was filtered, washed multiple times with water, and dried to obtain edge-selectively oxidized graphene nanoflake, which were used for subsequent characterization. X-ray diffraction was performed on Rigaku Smart Lab 3kW (Rigaku Corporation). FTIR was recorded on Nicolet iS50 (THERMO). XPS was conducted on Thermo Scientific ESCALAB 250Xi.

1.5. Molecular synthesis: Experiments were carried out under dry argon using standard Schlenk techniques and vacuum-line systems. Solvents were dried by standard methods and distilled prior to use. Chemicals were purchased from commercial vendors and used as received without further purification unless otherwise noted. ^{1}H, ^{13}C and ^{31}P NMR spectra were recorded on Bruker 400 or 500 MHz spectrometers at ambient temperature, with H_3PO_4 as the external reference for ^{31}P NMR. It should be noted that ^{13}C NMR data could not be obtained for **Ru 1** and **Ru 3** due to their poor solubility. The poor solubility of **Ru 1** could be attributed to the multiple Ph−C≡C−Ph units, which increase the molecular rigidity and promote intermolecular stacking. In the case of **Ru 3**, its multinuclear framework results in even lower solubility, precluding the acquisition of well-resolved ^{13}C NMR spectra. Elemental analysis was conducted on a varioEL cube instrument to verify the composition and purity of these two compounds, **Ru 1** and **Ru 3**. High-resolution mass spectrometry (HR-MS) was recorded on a Finnigan LCQ mass spectrometer or a Bruker MicrO-Tof-Q2 spectrometer. The organometallic **Ru 2** molecule was obtained following a reported procedure[1]. Organometallic **Ru 1** and **Ru 3** were synthesized as described below:

Synthesis of Ru 1:

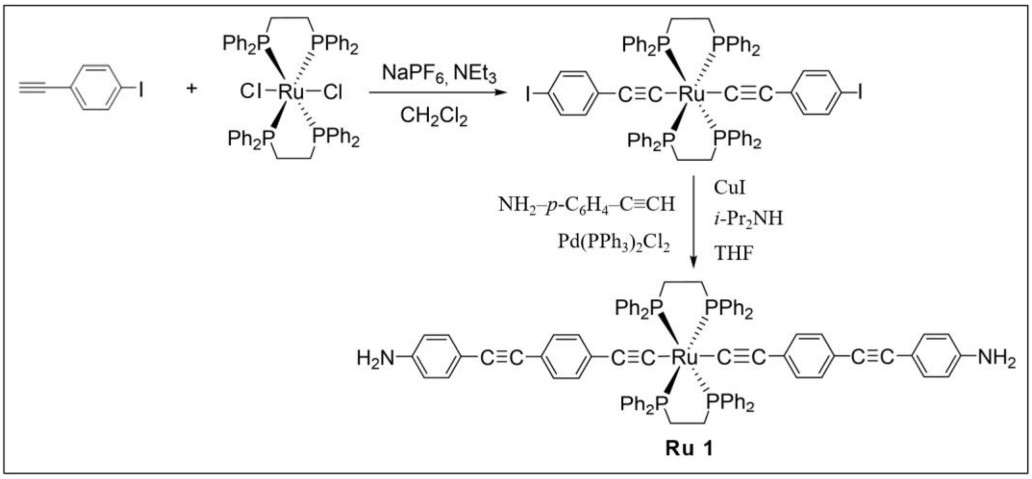

Scheme S1. Synthesis route of **Ru 1**.

Ru 1: Compound I–C_6H_4–C≡C–Ru(dppe)$_2$–C≡C–C_6H_4–I was prepared from $NaPF_6$, NEt_3, I–*p*-C_6H_4–C≡CH, and *cis*-[(dppe)$_2$RuCl$_2$] following a previously reported procedure[2]. I–C_6H_4–C≡C–Ru(dppe)$_2$–C≡C–C_6H_4–I (100 mg, 0.074 mmol), NH_2-*p*-C_6H_4-C≡CH (29 mg, 0.25 mmol), Pd(PPh$_3$)$_2$Cl$_2$ (3.2 mg, 0.005 mmol), and CuI (1.7 mg, 0.009 mmol) were added to a 100 mL two-necked round-bottom flask. Subsequently, 20 mL of THF and 20 mL of *i*-Pr$_2$NH were sequentially added to the reaction system. The reaction was stirred at room temperature under an argon atmosphere for 24 h. Upon completion, the reaction mixture was concentrated under reduced pressure to remove the organic solvent, yielding the crude product. The residue was then partitioned between CH_2Cl_2 and H_2O. The organic layer was collected, dried over anhydrous $MgSO_4$, and filtered. The filtrate was concentrated under reduced pressure to remove the solvent. The crude product was then washed sequentially with copious amounts of *n*-hexane (4 × 10 mL) and diethyl ether (2 × 10 mL) to afford the desired product as a brown solid (~60 mg, ~61%). **^{1}H NMR** (DMSO-*d*$_6$, 400 MHz, ppm): δ = 7.42 (m, 18H), 7.17–7.24 (m, 16H), 6.97 (t, J = 7.3 Hz, 14H), 6.71 (d, J = 7.9 Hz, 4H), 6.57 (d, J = 8.2 Hz, 4H), 5.53 (s, 4H), 2.64 (m, 8H). **^{31}P NMR** (DMSO-*d*$_6$, 162 MHz, ppm): δ = 51.39 (s). **IR** (KBr): v (cm^{-1}) = 1689 (C≡C), 2052 (C≡C). **HR-MS** (ESI) (*m*/*z*): 1330.3414 ([M]$^+$ calcd: 1330.3376). **Elemental analysis** (%) for $C_{84}H_{68}N_2P_4Ru \cdot CH_2Cl_2$: C, 72.03; H, 4.98; N, 1.98 (calcd: C, 72.13; H, 4.99; N, 1.98).

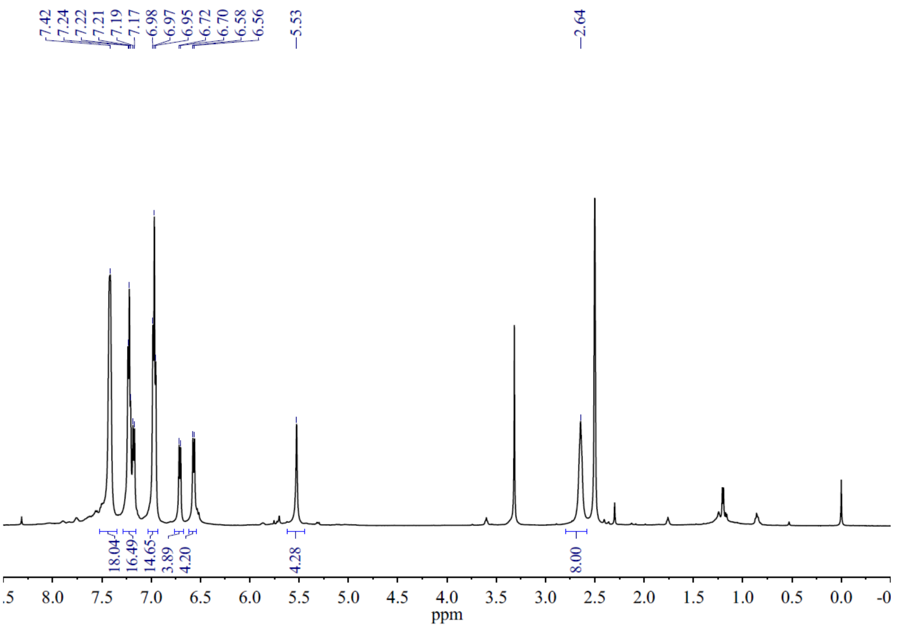

Figure S1. ^{1}H NMR spectrum of **Ru 1** in DMSO-d_6.

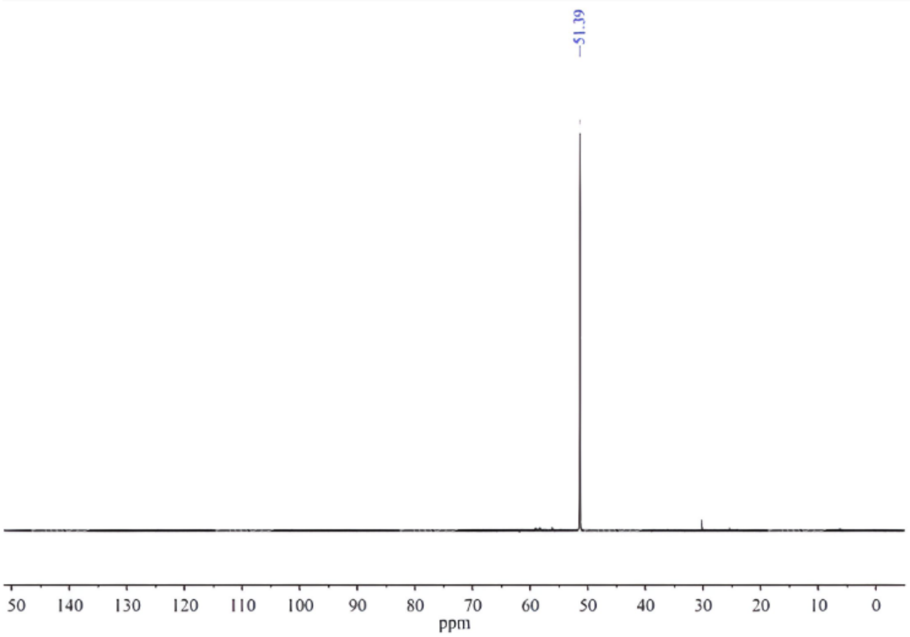

Figure S2. ^{31}P NMR spectrum of **Ru 1** in DMSO-d_6.

Synthesis of Ru 3:

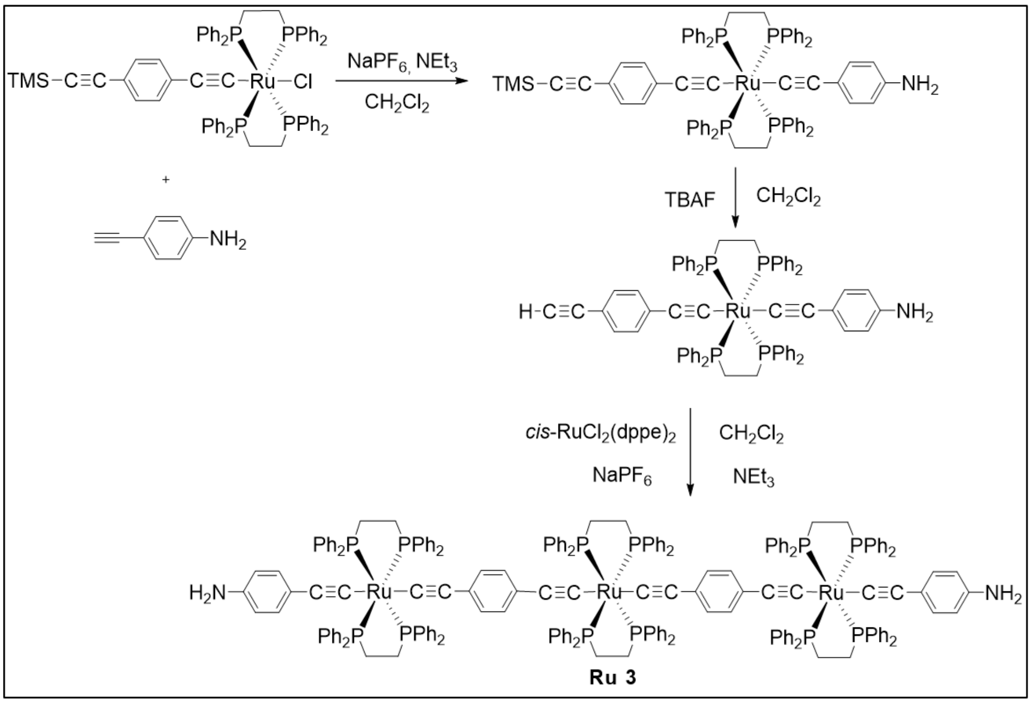

Scheme S2. Synthesis route of **Ru 3**.

***Trans*-[TMS-C≡C-C_6H_4-C≡C–Ru(dppe)$_2$–C≡C–C_6H_4–NH_2]:** In a Schlenk tube, *trans*-[TMS–C≡C–C_6H_4–C≡C–Ru(dppe)$_2$–Cl] (380 mg, 0.34 mmol), $NaPF_6$ (169 mg, 1.01 mmol), HC≡C–*p*-C_6H_4–NH_2 (59 mg, 0.50 mmol) were successively added with CH_2Cl_2 (60 mL) and NEt_3 (1.4 mL, 10 mmol). The reaction mixture was stirred for 5 days at room temperature. The solvent was then removed under vacuum. The residue was dissolved in CH_2Cl_2, and the product was precipitated with methanol. Then, the precipitate was washed with pentane (3 × 10 mL) to afford a yellow powder after removal of the residual solvent (~366 mg, ~90%). **^{1}H NMR** (CD_2Cl_2, 400 MHz, ppm): δ = 7.63 (d, J = 6.8 Hz, 8H), 7.37 (d, J = 6.9 Hz, 8H), 7.20–7.14 (m, 10H), 7.00–6.91 (m, 16H), 6.68 (d, J = 8.4 Hz, 2H), 6.60 (d, J = 8.2 Hz, 2H), 6.50 (d, J = 8.5 Hz, 2H), 3.56 (s, 2H), 2.64 (m, 8H), 0.26 (s, 9H). **^{13}C NMR** (CD_2Cl_2, 126 MHz, ppm): δ = 143.08 (s), 140.20 (m), 137.78 (m), 137.49 (m), 134.82 (s), 134.44 (s), 131.45 (s), 131.08 (s), 130.21 (s), 129.06 (s), 128.94 (s), 127.38 (s), 123.80 (m), 121.80 (s), 117.11 (br s), 117.02 (s), 116.84 (s), 114.96 (s), 106.75 (s), 93.88 (s), 31.86 (m), 0.20 (s). **^{31}P NMR** (CD_2Cl_2, 162 MHz, ppm): δ = 53.07 (s). **HR-MS** (ESI) (*m*/*z*): 1211.3042 ([M]$^+$ calcd: 1211.3037).

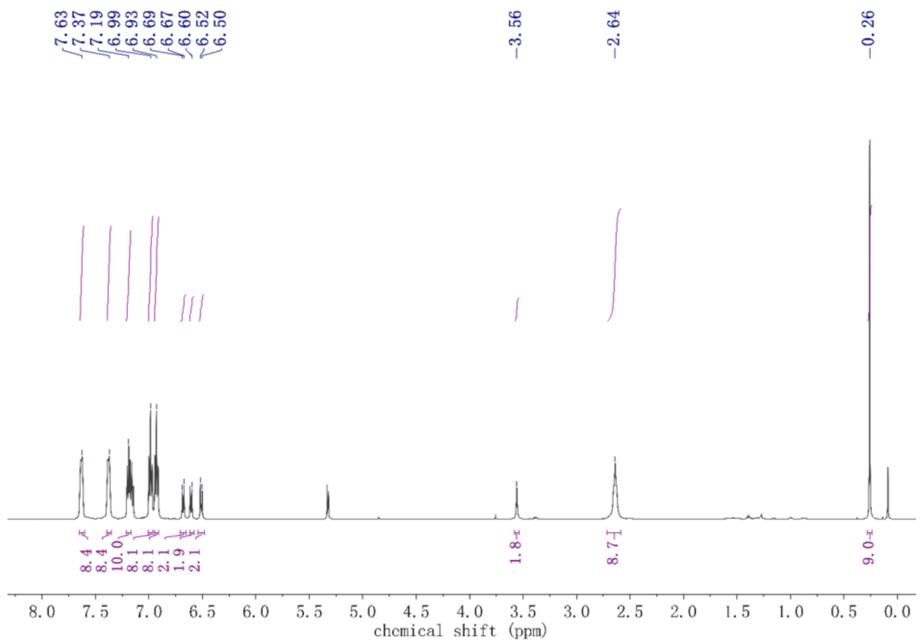

Figure S3. ^{1}H NMR spectrum of *trans*-[TMS–C≡C–C_6H_4–C≡C–Ru(dppe)$_2$–C≡C–C_6H_4–NH_2] in CD_2Cl_2.

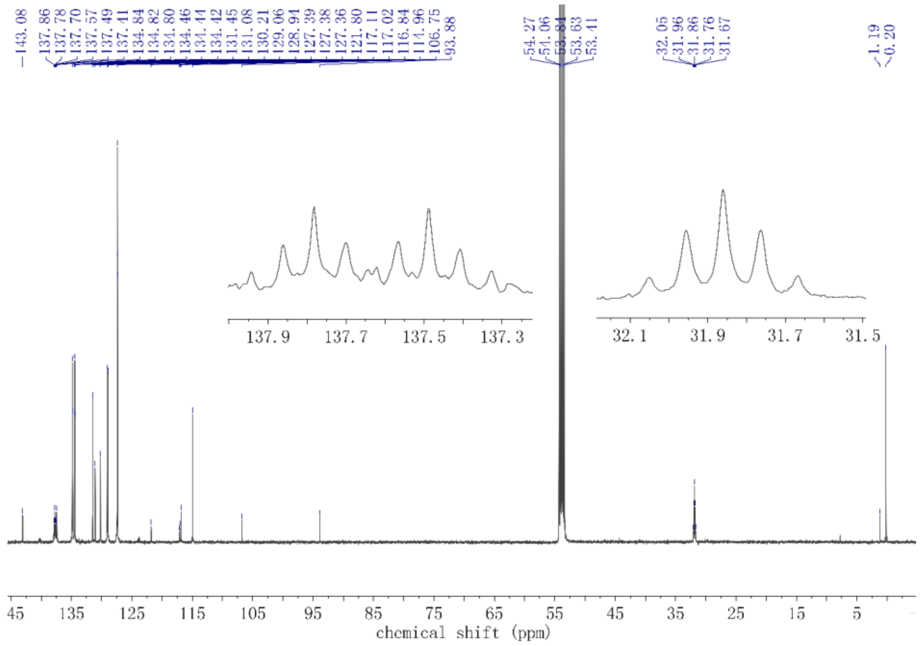

Figure S4. ^{13}C NMR spectrum of *trans*-[TMS–C≡C–C_6H_4–C≡C–Ru(dppe)$_2$–C≡C–C_6H_4–NH_2] in CD_2Cl_2.

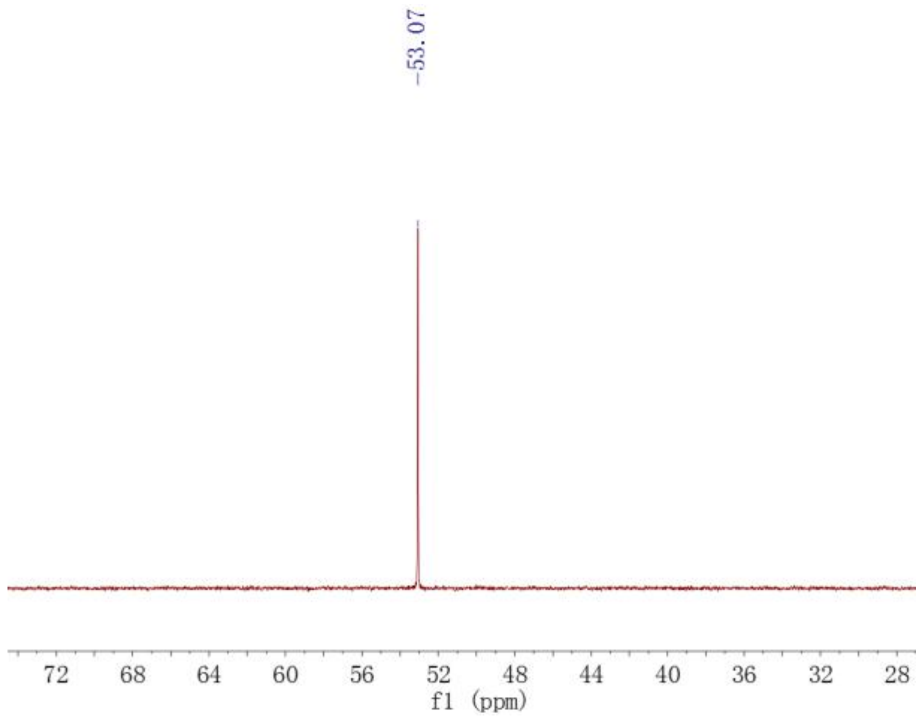

Figure S5. ^{31}P NMR spectrum of *trans*-[TMS–C≡C–C_6H_4–C≡C–Ru(dppe)$_2$–C≡C–C_6H_4–NH_2] in CD_2Cl_2.

***Trans*-[H–C≡C–C_6H_4–C≡C–Ru(dppe)$_2$–C≡C–C_6H_4–NH_2]:** In a Schlenk tube, *trans*-[TMS–C≡C–C_6H_4–C≡C–Ru(dppe)$_2$–C≡C–C_6H_4–NH_2] (366 mg, 0.30 mmol) and Bu_4NF (1 M in THF, 0.37 mL, 0.37 mmol) were dissolved in CH_2Cl_2 (130 mL). The solution was stirred for 1 h at room temperature and then evaporated to dryness. The residue was washed with distilled methanol (3 × 20 mL) and dried under vacuum. Then, a light-yellow powder was obtained (~200 mg, ~58%). **^{1}H NMR** (CD_2Cl_2, 400 MHz, ppm): δ = 7.67 (d, J = 6.6 Hz, 8H), 7.42 (d, J = 6.5 Hz, 8H), 7.24 (d, J = 8.2 Hz, 2H), 7.18–7.13 (m, 8H), 6.98–6.90 (m, 16H), 6.71 (d, J = 8.4 Hz, 2H), 6.66 (d, J = 8.2 Hz, 2H), 6.55 (d, J = 8.4 Hz, 2H), 3.60 (s, 2H), 3.19 (s, 1H), 2.68 (m, 8H). **^{13}C NMR** (CD_2Cl_2, 126 MHz, ppm): δ = 143.07 (s), 137.77 (m), 137.50 (m), 134.81 (s), 134.45 (s), 131.70 (s), 131.67 (s), 131.07 (s), 130.23 (s), 129.06 (s), 128.95 (s), 127.38 (s), 121.80 (s), 117.06 (s), 116.79 (s), 115.74 (s), 114.96 (s), 85.07 (s), 77.04 (s), 31.85 (m). **^{31}P NMR** ($CDCl_3$, 162 MHz, ppm): δ = 53.73 (s). **IR** (KBr): v (cm^{-1}) = 2058 (C≡C). **HR-MS** (ESI) (m/z): 1139.2636 ([M]$^+$ calcd: 1139.2641). **UV-Vis** [λ_{max}, (ε, 10^3 M^{-1}·cm^{-1}), toluene, nm]: 368 (34100).

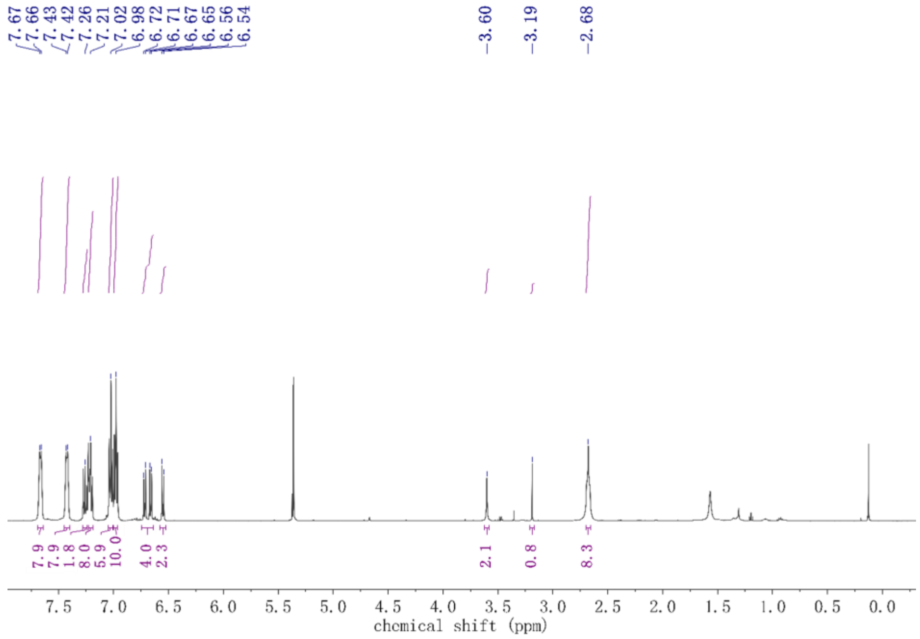

Figure S6. ^{1}H NMR spectrum of *trans*-[H–C≡C–C_6H_4–C≡C–Ru(dppe)$_2$–C≡C–C_6H_4–NH_2] in CD_2Cl_2.

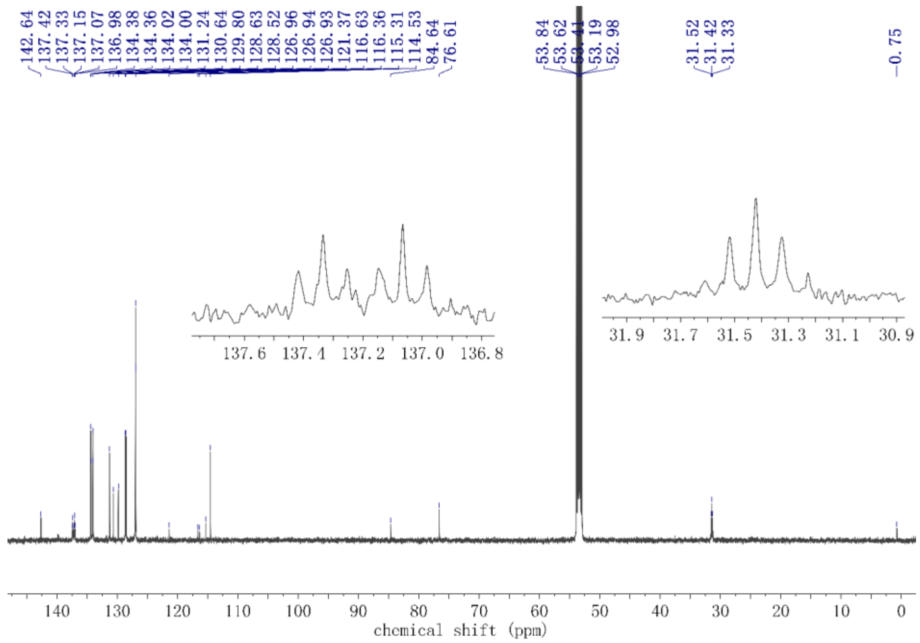

Figure S7. ^{13}C NMR spectrum of *trans*-[H–C≡C–C_6H_4–C≡C–Ru(dppe)$_2$–C≡C–C_6H_4–NH_2] in CD_2Cl_2. Note that the solvent signal found at ~53.41 ppm should be calibrated to the standard at ~53.84 ppm. Accordingly, all peaks should be increased by ~0.43 ppm to obtain the correct values.

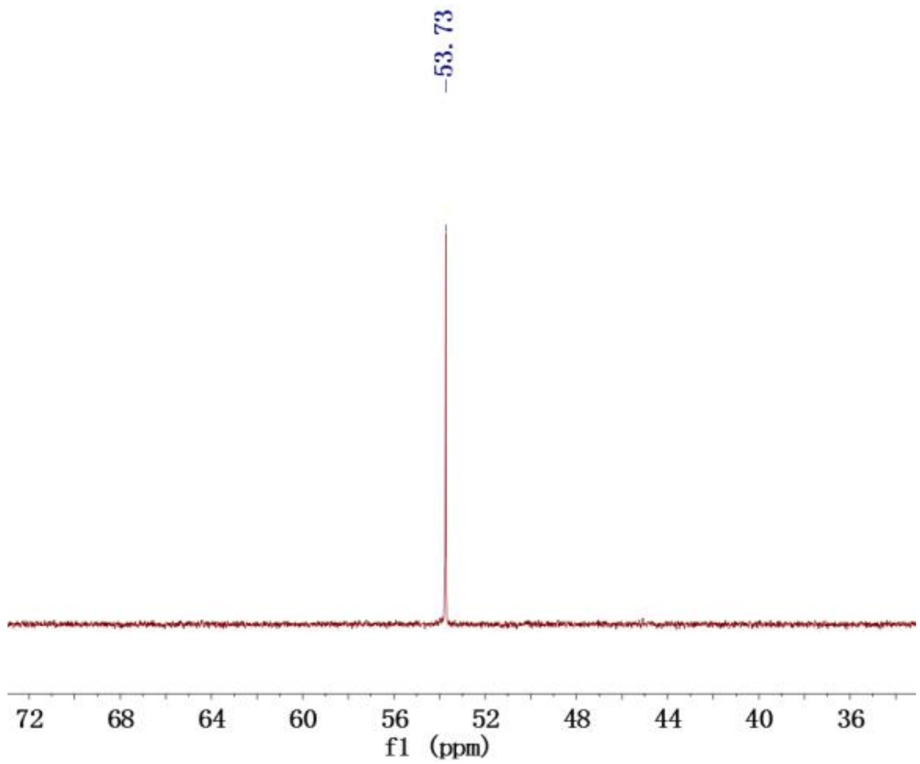

Figure S8. ^{31}P NMR spectrum of *trans*-[H–C≡C–C_6H_4–C≡C–Ru(dppe)$_2$–C≡C–C_6H_4–NH_2] in $CDCl_3$.

Ru 3: In a Schlenk tube, *trans*-[H–C≡C–C_6H_4–C≡C–Ru(dppe)$_2$–C≡C–C_6H_4–NH_2] (100 mg, 0.088 mmol), $NaPF_6$ (28.8 mg, 0.17 mmol), *cis*-$RuCl_2(dppe)_2$ (41.4 mg, 0.043 mmol) were introduced, and CH_2Cl_2 (10 mL) and NEt_3 (0.18 mL, 1.29 mmol) were further added. The reaction mixture was stirred for 3 days at room temperature, after which the solvent was removed under reduced pressure. The resulting residue was dissolved in CH_2Cl_2 and precipitated with methanol. The precipitate was collected and washed with pentane (3 × 10 mL). Removal of residual solvent under reduced pressure afforded the desired product as a yellow solid (~60 mg, ~44%). **^{1}H NMR** (benzene-d_6, 400 MHz, ppm): δ = 7.81 (m, 48H), 7.16–6.96 (m, 84H), 6.45 (d, J = 8.4 Hz, 4H), 2.81 (s, 4H), 2.66 (m, 24H). **^{31}P NMR** (benzene-d_6, 162 MHz, ppm): δ = 53.91 (s) and 53.74 (s). **IR** (KBr): ν (cm^{-1}) = 2058 (C≡C), 3442 (N–H). **HR-MS** (ESI) (*m*/*z*): 3174.6905 ($[M]^+$ calcd: 3174.6876). **Elemental analysis** (%) for $C_{192}H_{164}N_2P_{12}Ru_3 \cdot CH_2Cl_2$: C, 70.97; H, 5.12; N, 0.84 (calcd: C, 71.12; H, 5.13; N, 0.86).

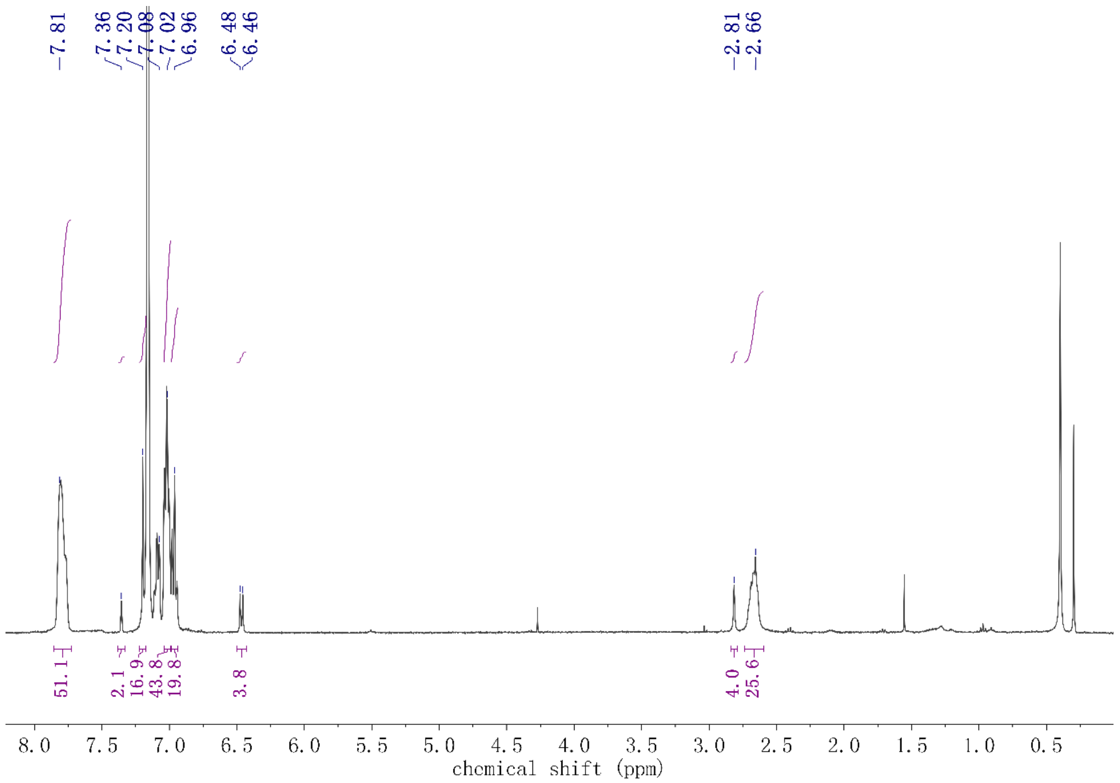

Figure S9. ^{1}H NMR spectrum of **Ru 3** in benzene-d_6.

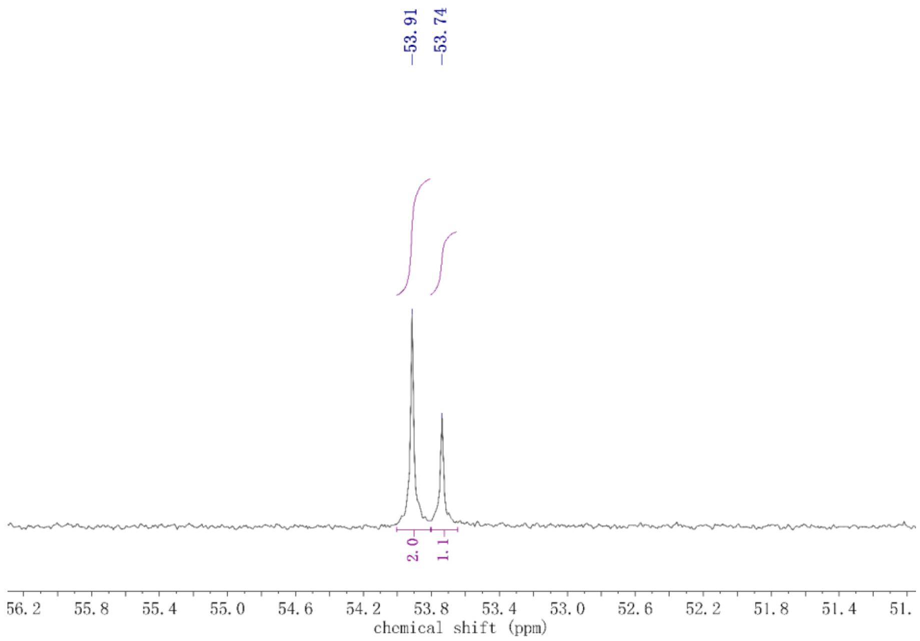

Figure S10. ^{31}P NMR spectrum of **Ru 3** in benzene-d_6.

1.6. Fabrication of Single-Molecule Devices: Place SiO_2/Si wafer containing edge-selectively oxidized triangular graphene electrodes into a two-neck bottle, then add 10^{-4} mol of organometallic Ru molecules and 11.5 mg 1-ethyl-3-(3-dimethylaminopropyl) carbodiimide hydrochloride (EDCI). Under the protection with inert gas, inject 10 mL of anhydrous pyridine into the mixture. After the reaction for 48 h, remove the device from the mixture and rinse with deionized water and acetone. After drying with N_2, the single-molecule device can be obtained.

1.7. Electrical Characterization of Graphene-Based Single-Molecule Junction: The electrical properties of the fabricated devices were tested by using the Agilent 4155C semiconductor parameter system (B1500A, Keysight) with manual probe station and vacuum cryogenic probe station scanned from –0.5 V to 0.5 V. The TTPX cryogenic probe stage was DCH-196 from Uotest Electronic Technology (Shenzhen) Co., Ltd. The temperature control module of the hot and cold chuck was HCC214S from INSTEC. The temperature-dependent I_D−V_D curves were measured in vacuum environment at the sweep interval of 0.05 V/step. A temperature-control module with cooling system based on liquid N_2 was used to regulate the device temperature from 80 K to 300 K.

1.8. Electrical Characterization with STM-BJs: STM-BJ consists of an Au tip, an Au substrate and molecular solutions. The Au tip was prepared by electrochemical etching of an Au wire. Target molecules were dissolved in 1,2,4-trichlorobenzene (TCB, anhydrous, ≥99% purity, Aladdin) to form a molecular solution (0.1 mM). The substrate was fabricated by thermal evaporation. 8 nm Cr and 200 nm Au were deposited onto the Si/SiO_2 wafer (300 nm SiO_2 layer) at ~1 Å/s, respectively. The electrical characterization of STM-BJ was measured in solution at a bias voltage of 0.1 V. In the STM-BJ experiment, the tip's movement was controlled by a stepper motor and a piezoelectric stack. When a bias voltage was applied between the tip and the substrate, the conductance was used as the feedback signal of the piezoelectric stack. Thus, the distance gap between the Au tip and the substrate was controlled. The conductance versus displacement traces were then collected during repeated cycles of molecular junction formation and destruction. All measurements were taken at room temperature.

1.9. Theoretical Calculation: Geometrical optimizations were performed using the density functional theory combined (DFT) with non-equilibrium Green's functions (NEGF) to simulate molecular junction evolutions. The molecular geometries and molecular orbital distributions were obtained by the Gaussian 16 based on the DFT with the B3LYP functional with 6-31g(d) for C, H, N, P and SDD basis set for Ru[3,4]. The theoretical transport properties were obtained by first-principles calculations (Quantum Atomistix ToolKit software package, T-2022.03 version) employing the combination of the DFT treatment for the electronic structure and the NEGF formalism in the simulation of coherent transport[5]. FHI pseudopotential and double-zeta polarized atomic orbital basis set was applied in the NEGF-DFT calculation, and exchange-correlation was treated at the Perdew-Burke-Ernzerh (PBE) generalized gradient approximation level (GGA+PBE). The cut-off energy for the real-space grid was set to be 125 Hartree, and the k-point grid was set to be 4 × 1 × 150 as default value. The NEGF-DFT self-consistent calculations were deemed converged when every element of the Hamiltonian matrix and the density matrix converged to less than 10^{-5} a.u. For the calculation of the transmission spectra, the k-point sampling was set as 15. The MPSH spectra of the four states near the graphene Fermi level were also calculated to assign the dominant transport channels, and the isovalue was set at 0.03 $Å^{-3/2}$. The two Au (111) electrodes consist of a unit cell 5 × 5 and the extension region thickness are six to guarantee the calculation accuracy; a pyramidal Au atoms cluster was employed to fabricate stable contacts with the methylthio anchors on both electrode sides. The exchange-correlation Generalized Gradient Approximation (GGA) with the PBE parameterization was selected for molecular device calculations. The double-ξ basis set was employed for k-atoms, and the double-ξ polarization basis function was used for the other atoms. The energy cutoff was set to 150 Ry, which is enough for the Au electrode system. The geometry optimization at the zero bias was optimized to the convergence criterion of 0.05 eV/A. The k-point with a grid of (4 × 4 × 150) was used in the calculation of the transmission spectra.

2. Fabrication of Atomically Precise Graphene Electrodes

2.1 Schematic Diagram of the Fabrication of Graphene Electrodes

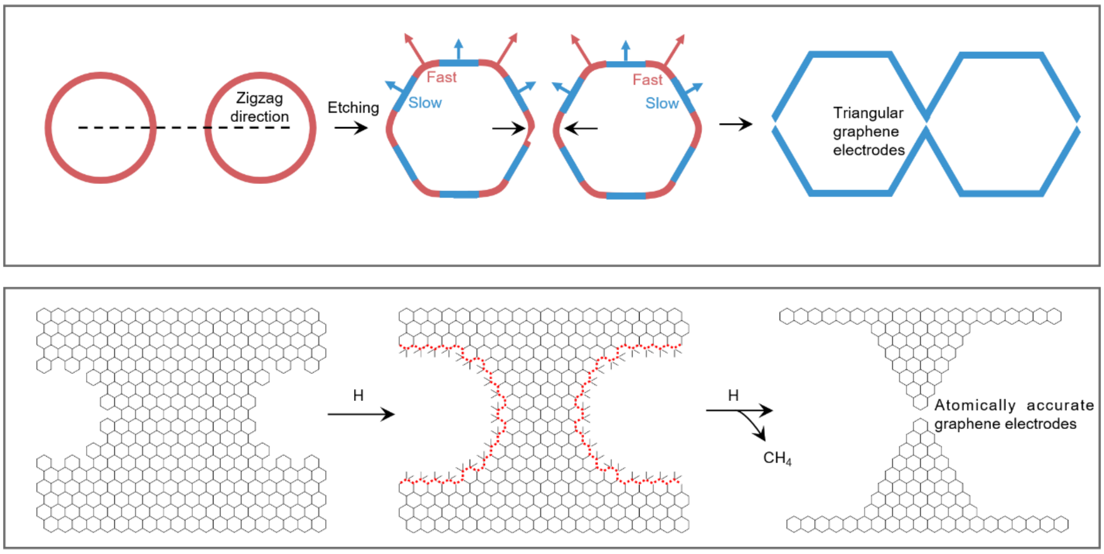

Figure S11. Mechanism of the preparation of atomically precise gaps and zigzag-edged graphene electrodes.

In remote hydrogen plasma environment, hydrogen atoms chemisorb onto the graphene edges, catalyzing a transition of carbon atoms from sp^2 to sp^3 hybridization, as a thermodynamically favorable process. This tetrahedral configuration of the sp^3 state triggers local lattice deformation, leading to C–C bond breakage and the subsequent formation of $–CH_3$ and $–CH_2$ groups. The suspended $–CH_3$ group, unstable at high temperatures, tends to undergo further hydrogenation, generating CH_4 gas and thus completing an etching cycle. In addition, the zigzag edge configuration in graphene represents a thermodynamically more stable state, characterized by the lowest hydrogen etching rate. Consequently, during the etching process, other edge configurations of graphene will be etched away at a faster rate, promoting a higher proportion of zigzag units in the edge configuration, and ultimately resulting in the formation of hexagonal holes. The adjacent vertices of these hexagonal holes then interconnect to form graphene triangular point electrodes with molecular-scale gaps.

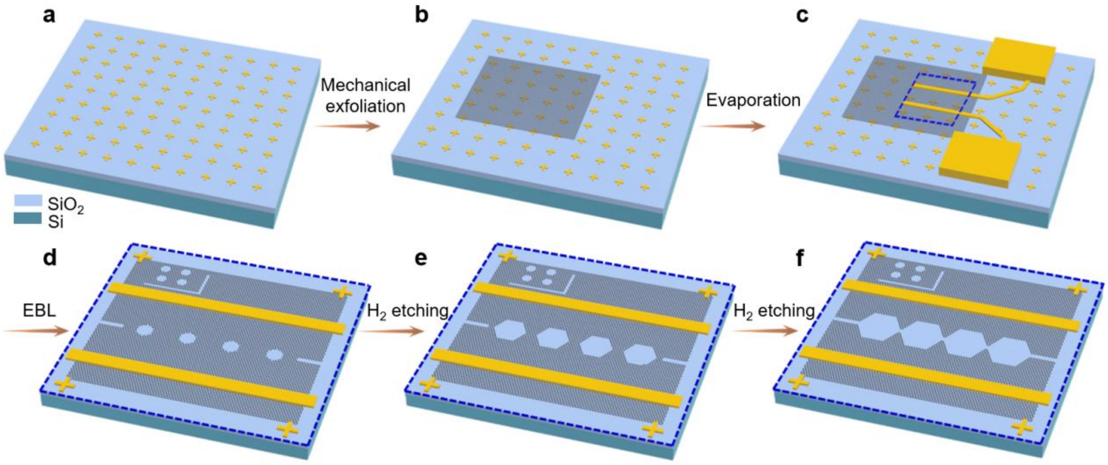

Figure S12. Schematic illustration of the preparation of atomically accurate graphene electrodes. (a) SiO_2/Si wafer with '+' markers. (b) Mechanically exfoliated graphene on SiO_2/Si wafer. (c) Fabrication of metal electrodes. (d–f) Formation process of graphene triangular point electrodes by stepwise etching.

2.2 Fabrication of the Graphene Samples

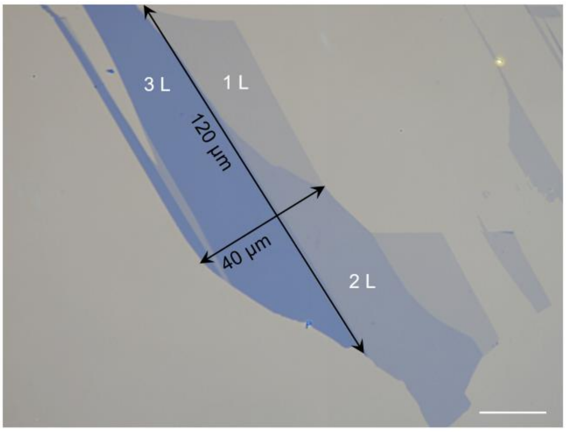

Figure S13. Determination of the layer number of the graphene sheets. The optical image of mechanically exfoliated graphene with different layers. 1 L, 2 L, and 3 L represent the single-layer, double-layer, and three-layer graphene sheets, respectively. Scale bar, 20 μm.

A mark pattern consisting of 8 nm Cr (99.999% purity) followed by 80 nm Au (99.999% purity) was deposited on the SiO_2/Si wafer (300 nm SiO_2 layer) to facilitate the subsequent determination of the position of mechanically exfoliated graphene. The photolithography process was performed using the MJB4 from SUSS MicroTec, and the thermal evaporation coating was conducted using the GSL-1800X-ZF4 from KeJing Automation Equipment (Shenyang) Co., Ltd. The graphene samples were placed on the substrate by the mechanical exfoliation method. Optical images of graphene samples were captured using a standard optical microscope (BX53M, Olympus). The gray values of graphene (G_{gr}) and the substrate (G_{sub}) were obtained using the software Image J. The image contrast (r) can be calculated according to the following formula, and the layer of graphene can be determined accordingly:[6]

$$r = 1 - \frac{G_{\mathrm{gr}}}{G_{\mathrm{sub}}}$$

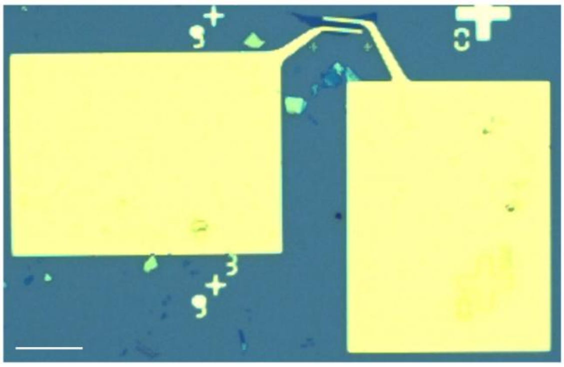

Figure S14. Optical image of a graphene device with gold electrodes. Electrodes fabricated using e-beam lithography, thermal evaporation and e-beam evaporation techniques, depositing layers of 8 nm Cr, 80 nm Au, and 40 nm SiO_2. Scale bar, 50 µm.

2.3 Accurate Preparation of Graphene Triangular Electrodes

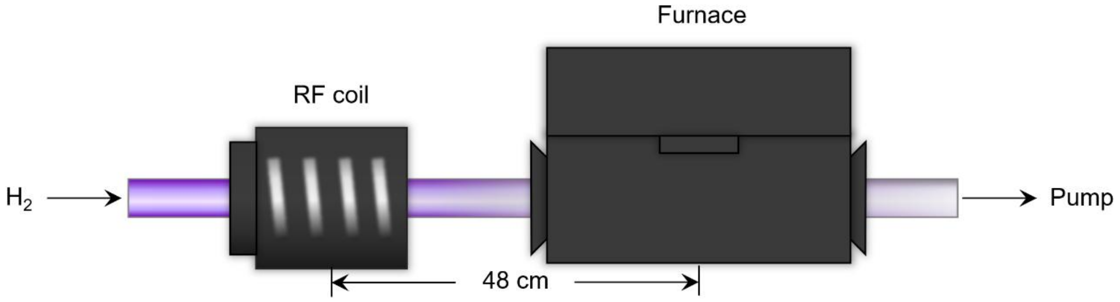

Figure S15. Schematic diagram of the laboratory-built plasma-enhanced hydrogen etching system. An inductively coupled radio-frequency (RF) coil is used as the hydrogen-plasma source, providing continuously variable intensity of the RF power. A 5 cm quartz-tube furnace is used to heat the SiO_2/Si wafer to the desired temperatures. The coil is positioned 48 cm from the center of the furnace, and the etching process is carried out at a temperature of 500 °C, with an etching power of 8 W and a hydrogen-flow rate of 10 sccm.

Anisotropic hydrogen plasma etching of graphene was performed using a laboratory-built plasma-enhanced hydrogen etching system. The coil was fixed at a distance of 48 cm from the center of the furnace to ensure that high-energy ions, such as H^+, H_2^+, and H_3^+, were fully attenuated, leaving only less demanding hydrogen atoms[7].

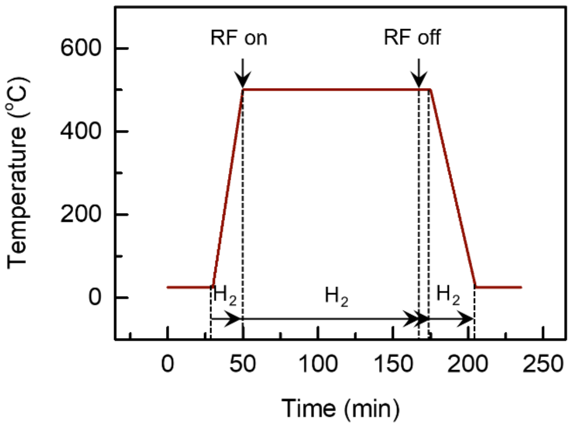

Figure S16. Schematic diagram of the pre-etching process using an inductively coupled RF coil. The pre-etching process is conducted in a laboratory-built plasma-enhanced hydrogen etching system. Firstly, the chip is heated to 500 °C in a hydrogen atmosphere with a flow rate of 10 sccm. Then, the RF power is turned on with an etching power of 8 W for 2 h to generate hydrogen atoms for the anisotropic etching of graphene. At the end of the anisotropic etching, the RF power is turned off and the system is held at 500 °C for 5 minutes. Finally, the heating is turned off, and once the quartz-tube furnace has cooled to room temperature, the hydrogen gas is shut off.

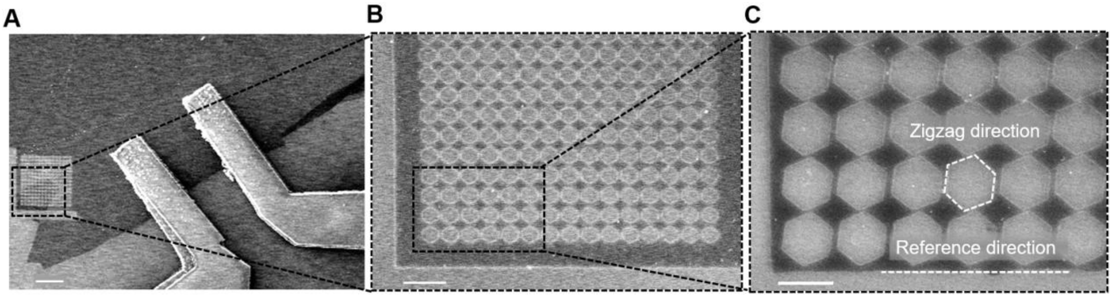

Figure S17. Pre-etching process to determine the zigzag-lattice direction of graphene. (a–c) Scanning electron microscope (SEM) images of hexagonal etching holes after pre-etching and its enlarged images. Scale bars, 4 μm (a); 1 μm (b); 500 nm (c). The white hexagonal frame indicates the zigzag direction, while the white line serves as the reference direction for angle measurements during the etching process. The zigzag direction is ascertained by quantifying the angular deviation between the zigzag edges and the reference direction.

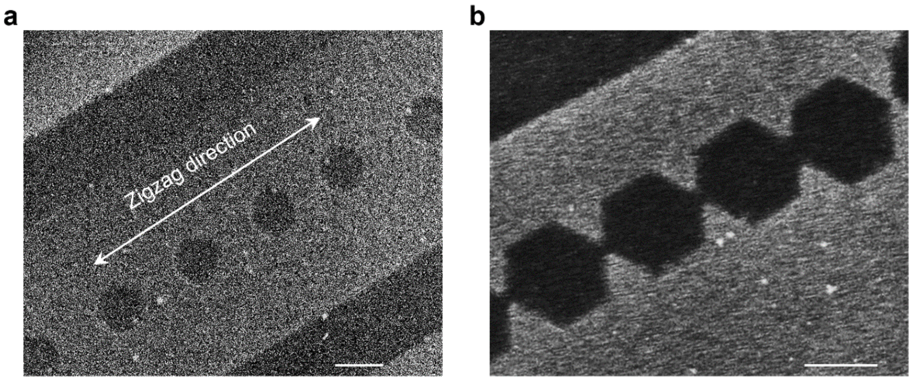

Figure S18. Formation process of graphene-based electrodes. (a) SEM image of a circular hole array with the same zigzag-lattice orientation of graphene. (b) Well-defined graphene triangular electrodes. All scale bars are 200 nm.

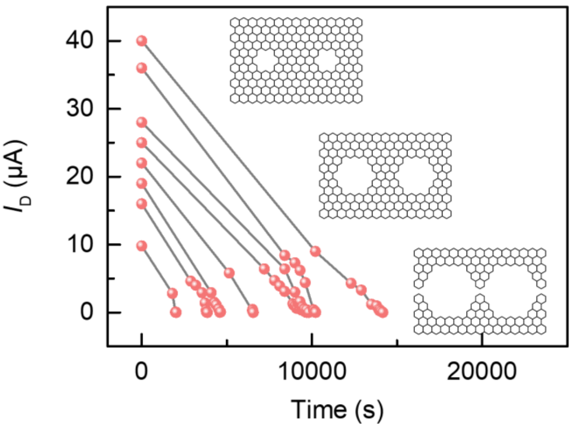

Figure S19. Stepwise-etching process by monitoring the current after a certain period of etching time. Stepwise-etching methods are used to avoid the problem of uncontrollable gaps after etching. During the first etching, a fixed etching time is set to transform the circular holes into hexagonal holes. At this point, there is still a certain distance between the vertices of adjacent hexagons. Then, by in-situ monitoring the current of the graphene electrodes on both sides, the width of the groove is preliminarily estimated, so as to set the next etching time. Repeat this process until the conductivity drops to the noise level (~10 pA), and the graphene channel is completely cut off, forming a triangular point electrode pair with a controllable gap.

During the etching process, it is necessary to avoid excessive etching, which can lead to a large gap and the molecule cannot be successfully connected. At the same time, it is also necessary to avoid the case that the gap is not formed due to the insufficient etching. Due to the inevitable discrepancy between the developed holes and the initial set size, the gap size cannot be accurately determined by the etching rate of hydrogen plasma. Therefore, we adopt a stepwise electrical feedback etching strategy, in which the entire etching process is divided into multiple short cycles. After each cycle, the device is taken out for ex-situ conductance measurements, providing an electrical feedback mechanism that enables precise control of the etching process. As the etching proceeds and the conductive graphene channel narrows, the device resistance increases and the measured current correspondingly decreases. By dynamically adjusting the subsequent etching duration based on the current reading after each step, we can accurately control the etching process and reliably avoid both over-etching and under-etching.

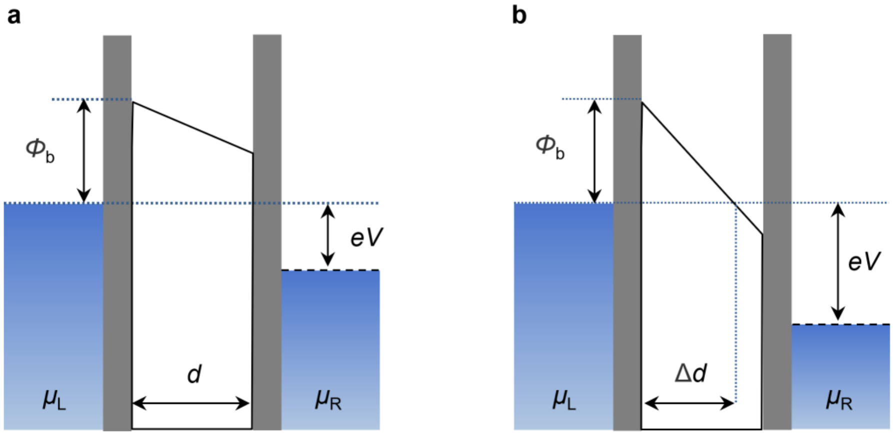

Figure S20. Potential barriers between graphene electrodes. (a) Rectangular barrier with height $eV < \Phi_b$. (b) Triangular barrier with height $eV > \Phi_b$, this leads to an effective barrier width Δd.

The model uses a generalized formula to describe the electrical tunneling effect between electrodes separated by a thin insulating layer. When a gap is formed, a vacuum insulation layer with high potential barriers is generated between the two electrodes. When a bias voltage is applied, the current flowing through the nanogap is a result of electron tunneling between the two triangular graphene electrodes. For rectangular barrier (Figure S20a), the Simmons model can be expressed as:

$$I_D = \frac{Ae}{4\pi^2\hbar d^2}\left\{\left(\Phi_b - \frac{eV}{2}\right) exp\left(\frac{-2d}{\hbar}\sqrt{2m}\sqrt{\Phi_b - \frac{eV}{2}}\right) - \left(\Phi_b + \frac{eV}{2}\right) exp\left(\frac{-2d}{\hbar}\sqrt{2m}\sqrt{\Phi_b + \frac{eV}{2}}\right)\right\}$$

where $\hbar$, e, m, and V_D are the reduced Planck's constant, the elementary charge, the electron mass, and the applied bias, respectively. At small biases, the tunneling barrier is generally trapezoidal. As the bias increases, the shape of the potential barrier gradually changes from trapezoidal to triangular (Figure S20b), then the corresponding tunneling process becomes Fowler-Nordheim tunneling, and this formula is no longer valid. Therefore, to more accurately describe the nanogap size, we chose a small bias (± 0.6 V) for the fitting. The three parameters

used for the Simmons fitting are the area of the junction A, the height of the rectangular barrier Φ_b and the nanogap size d. In our devices, the area of the junction is comparable to the area size of a single atom, $A = 0.01\ nm^2$. Therefore, only the barrier height and the effective nanogap size serve as the fitting parameters according to the measured I_D–V_D properties. The change in the gap size will cause a considerable variation in the tunneling current, and for a large d, this will no longer be measurable.

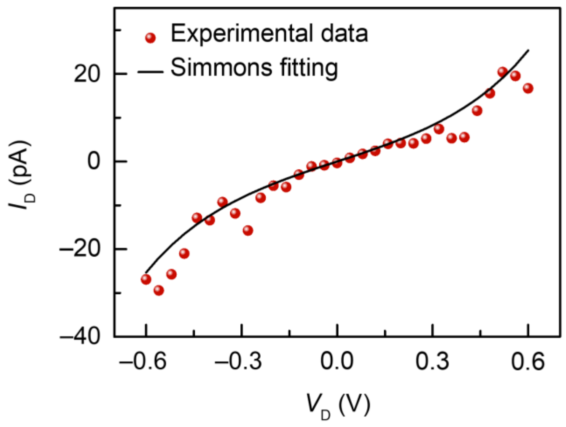

Figure S21. The gap size determined by Simmons fitting. The I_D–V_D characteristics of an empty device. According to the I_D–V_D fitting equation, a gap size of 1.09 nm can be determined.

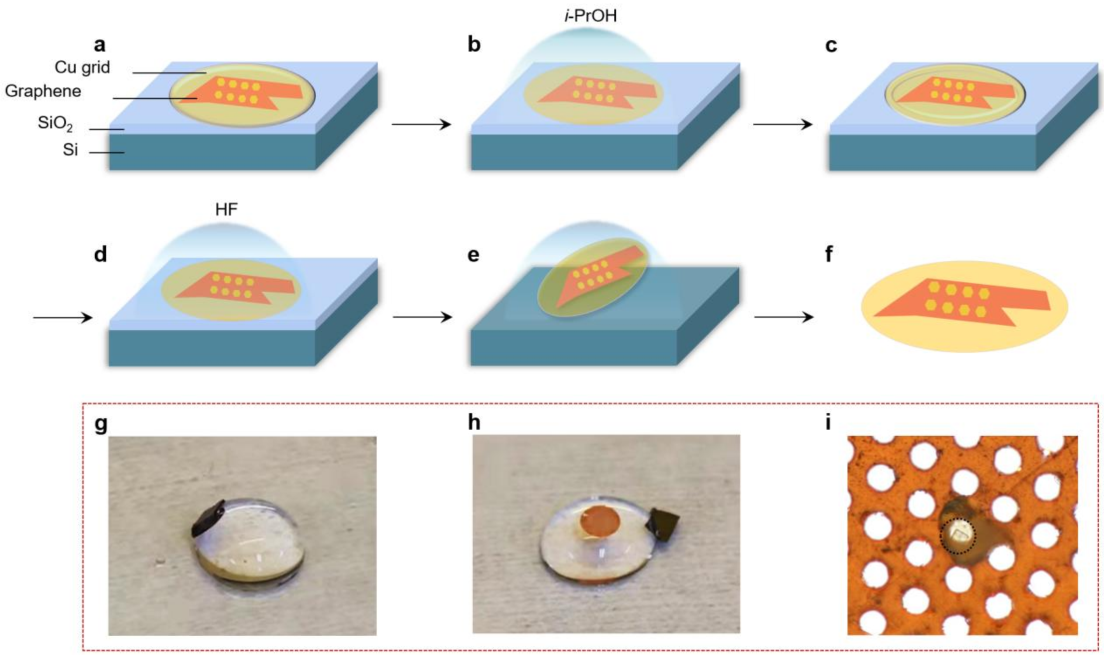

Figure S22. Schematic diagram of TEM sample preparation.

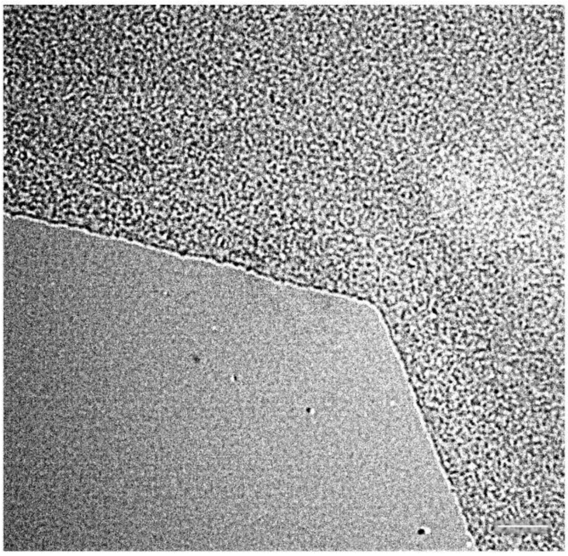

Figure S23. A high-resolution transmission electron microscope (HR-TEM) image of a hexagonal-edge graphene generated by remote hydrogen plasma etching. Scale bar, 50 nm.

2.4 Controllable Edge-Selective Modification of Graphene Electrodes

A mixture of 5 mL deionized water and 0.75 mL 30% H_2O_2 was added to the SiO_2/Si wafer with graphene triangular electrodes, and stirred at 0 °C for 0.5 h. Then add 0.5 mL H_2SO_4 dropwise and let stand for another hour in an ice bath. Subsequently, remove the ice bath and continue to stand for 4 h. After the reaction, clean the wafer with deionized water and dry it with N_2 to obtain controllable edge-selectively oxidized triangular graphene electrodes.

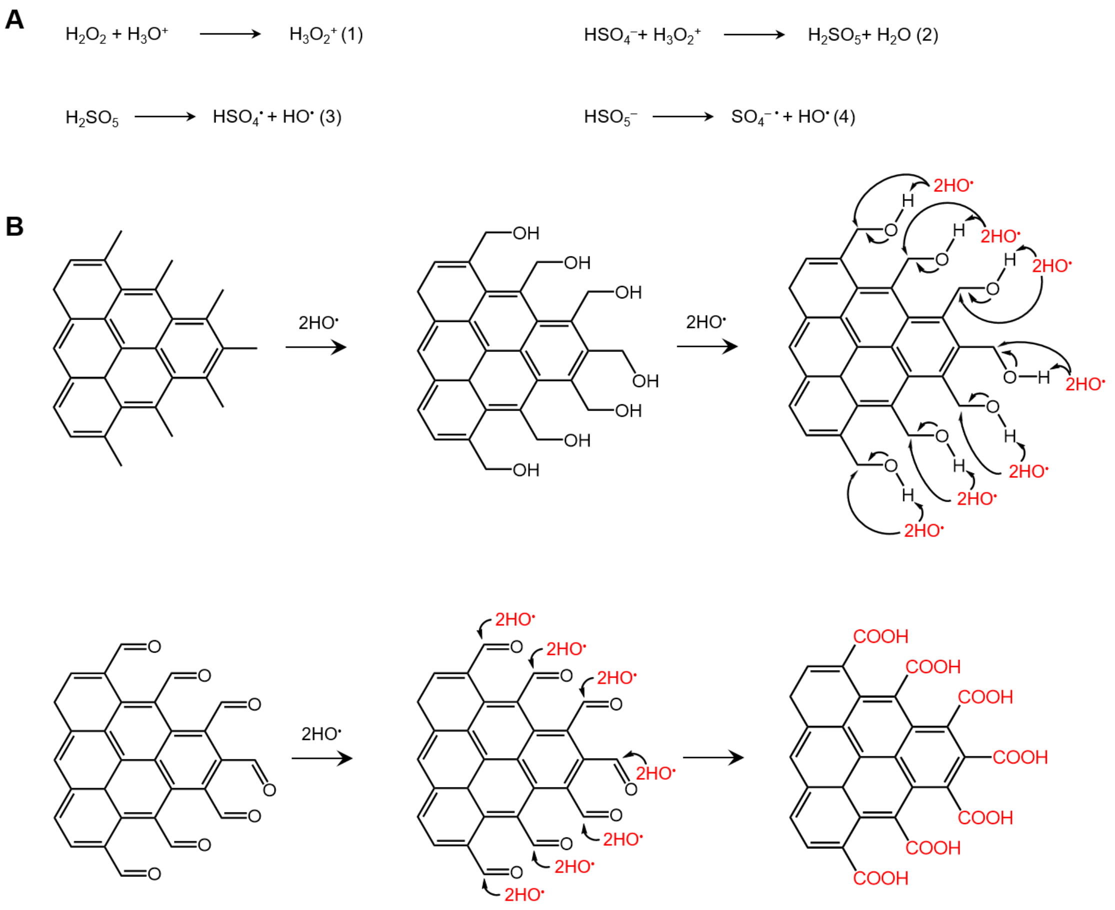

Figure S24. Edge-selective oxidation of graphene electrodes. (a) Reaction pathways of H_2O_2 mixing with H_2SO_4 and decomposed to $HO^{\bullet}$. (b) Mechanism for edge-selective oxidation of graphene triangular electrodes.

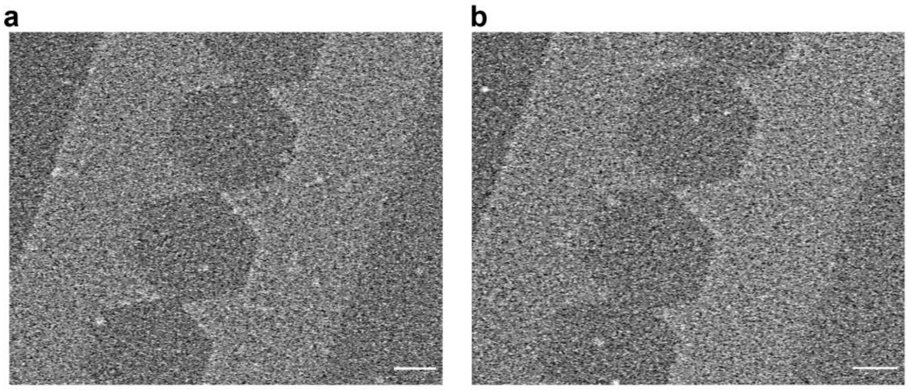

Figure S25. Controllable oxidation of graphene triangular electrodes. SEM images of triangular graphene electrodes before (a) and after (b) edge-selective oxidation. All scale bars are 50 nm. After the oxidation, the SEM images reveal no discernible changes, indicating that the graphene electrodes are not damaged.

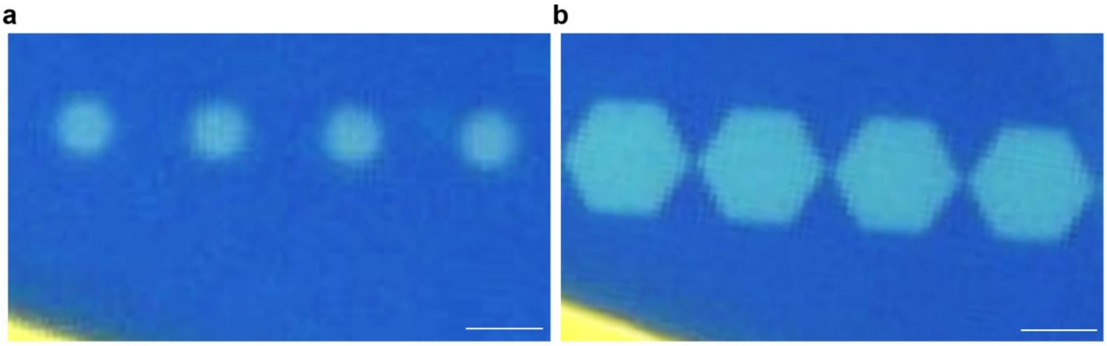

Figure S26. Preparation process of the sample for Raman mapping. Optical images of mechanically exfoliated graphene before (a) and after (b) plasma-enhanced hydrogen etching on SiO_2/Si wafer. All scale bars are 2 μm.

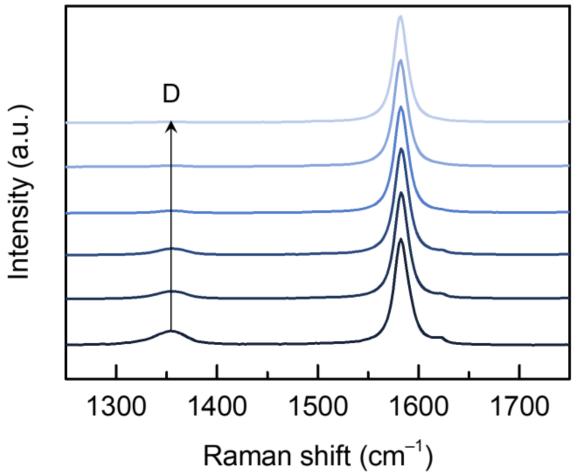

Figure S27. Normalized Raman spectra of edge-selectively oxidized graphene from edge area to center area. The *D*-band intensity is prominent near the edge, while it is relatively weak in the basal plane.

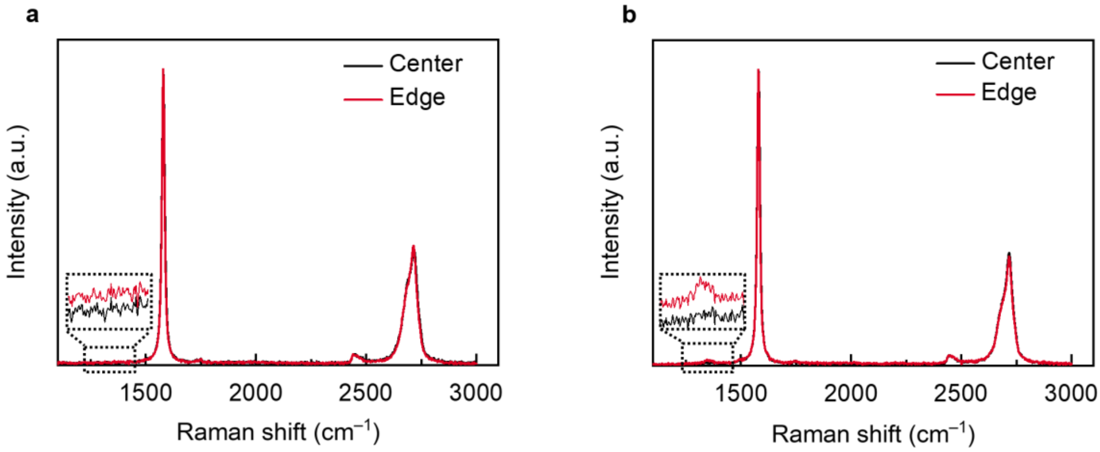

Figure S28. Single-point Raman spectra. Raman spectra of pristine graphene (a) and edge-selectively oxidized graphene (b) measured at edge and center regions.

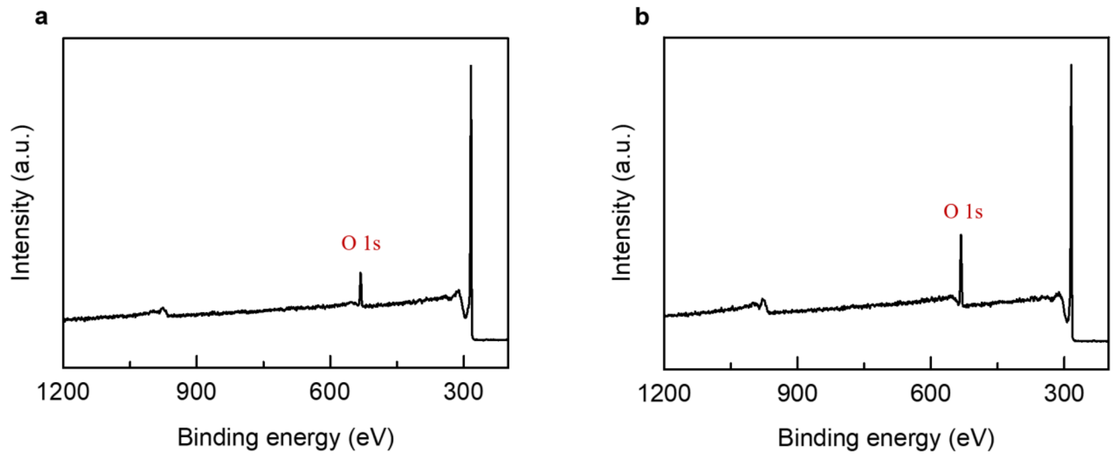

Figure S29. X-ray photoelectron spectroscopy (XPS) spectra of pristine graphene (a) and edge-selectively oxidized graphene (b).

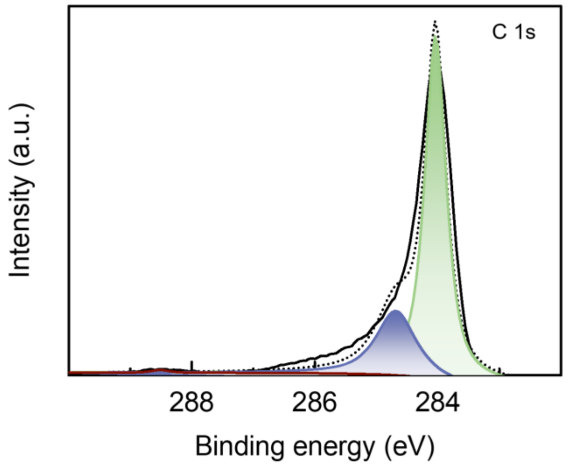

Figure S30. C 1s XPS spectrum of graphene nanoflakes.

3. Molecular Connection

3.1 Characterization of Organometallic Ru Molecules

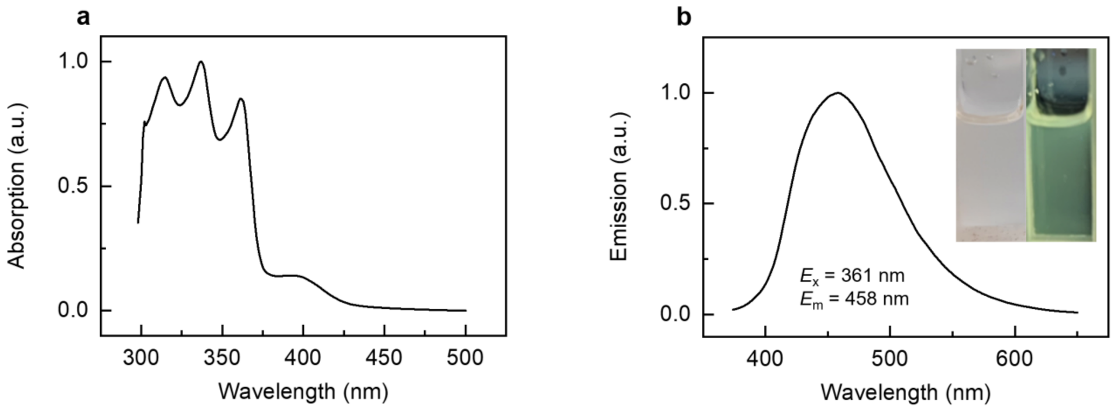

Figure S31. Excitation and emission spectra of **Ru 1**. (a) UV-Vis absorption and (b) photoluminescence spectra of **Ru 1** in TCB. Insert: photograph of the **Ru 1** solution under white light (left) and UV light (right).

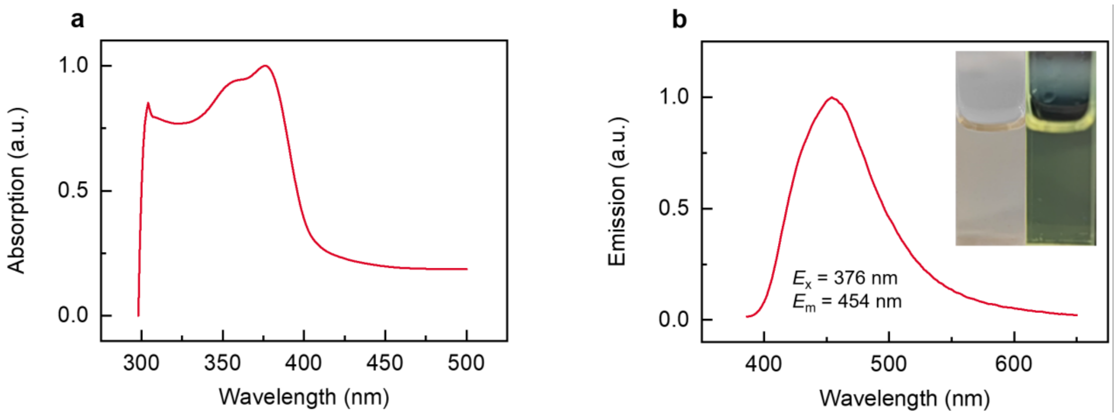

Figure S32. Excitation and emission spectra of **Ru 2**. (a) UV-Vis absorption and (b) photoluminescence spectra of **Ru 2** in TCB. Insert: photograph of the **Ru 2** solution under white light (left) and UV light (right).

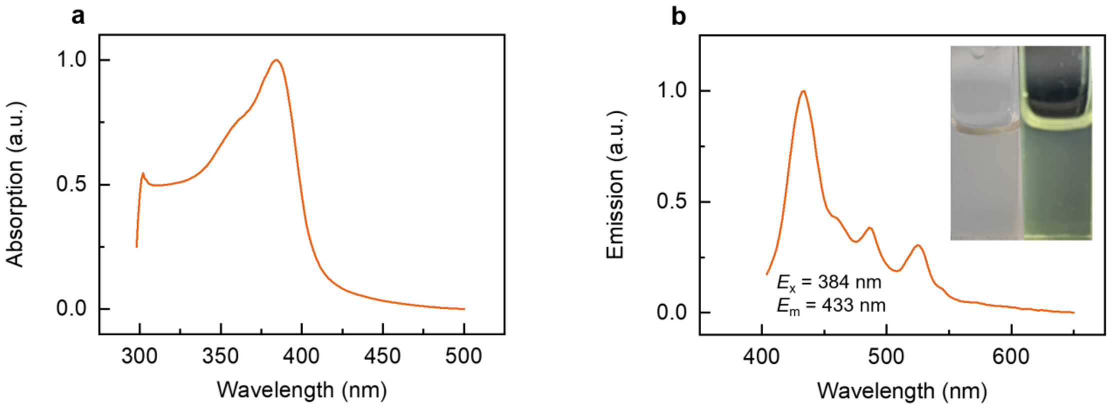

Figure S33. Excitation and emission spectra of **Ru 3**. (a) UV-Vis absorption and (b) photoluminescence spectra of **Ru 3** in TCB. Insert: photograph of the **Ru 3** solution under white light (left) and UV light (right).

3.2 Characterization of Molecular Connection in Devices

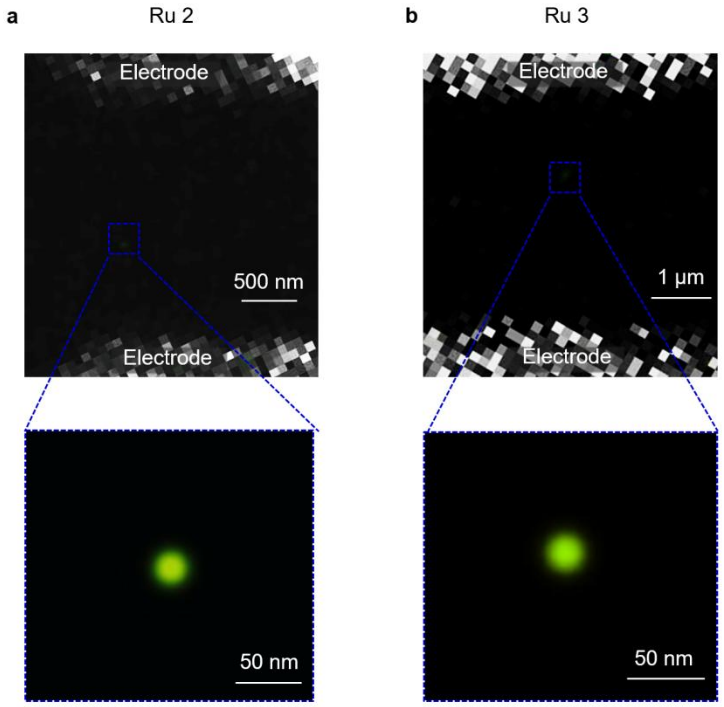

Figure S34. Superhigh-resolution fluorescent image of the single-molecule site by stochastic optical reconstruction microscopy (STORM). The devices are irradiated by a 405 nm, 5 mW laser through a 100 × oil lens with 5000 photos taken within an exposure time of 50 ms.

4. Device Characteristics

4.1 Graphene-based Single-Molecule Junctions

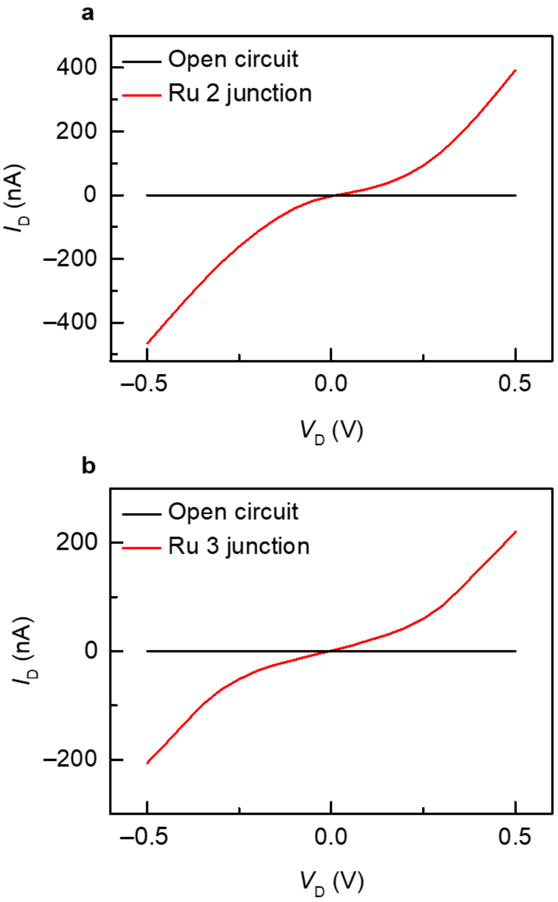

Figure S35. Electrical characteristics of the single-molecule connection. I_D–V_D curves of open circuits (black) and single-molecule devices after the molecular connection (red) of **Ru 2** (a), **Ru 3** (b).

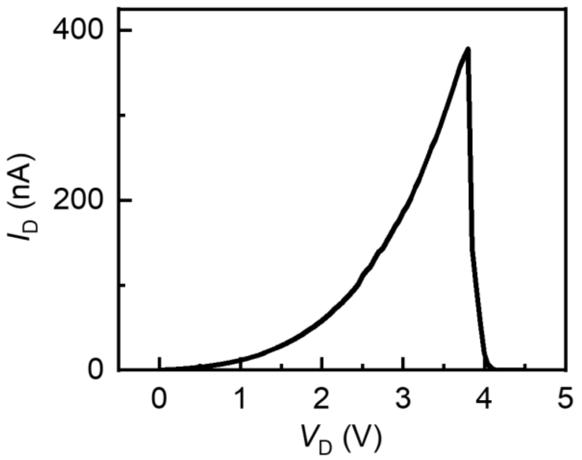

Figure S36. The *I–V* curve of a representative **Ru 1** single-molecule device over a wide bias range.

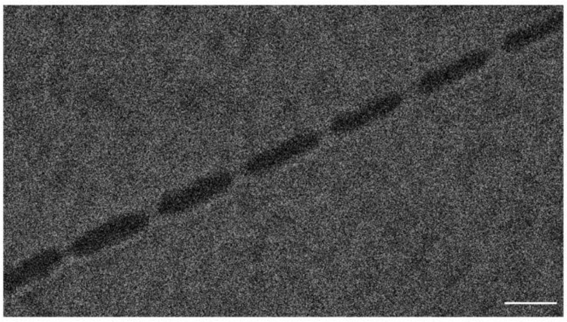

Figure S37. Graphene electrodes with traditional configurations. SEM image of graphene electrodes prepared by the DLL method. Scale bar, 100 nm.

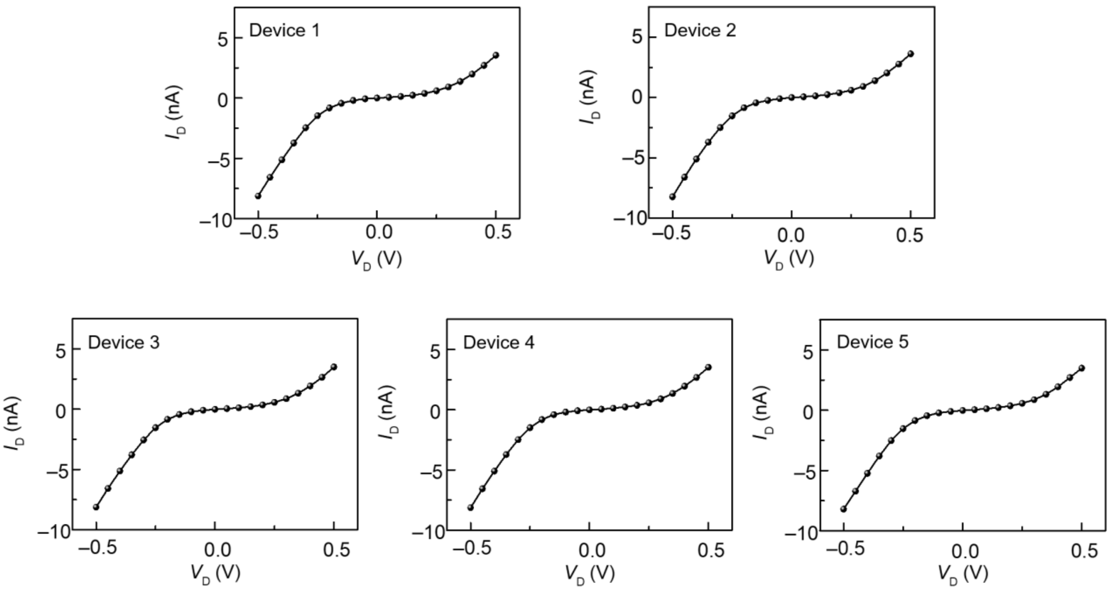

Figure S38. I_D–V_D curves of Ru 1 devices 1~5 fabricated by the AHPE method. The normalized standard deviation (SD) at 0.1 V is 1.04%. These data were obtained from multiple independently fabricated device batches.

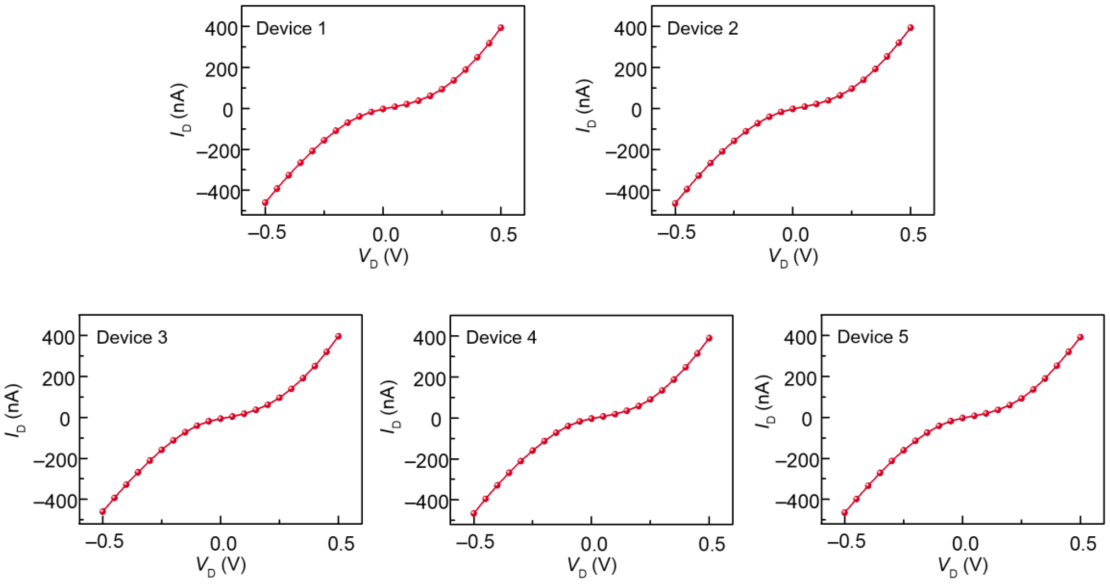

Figure S39. I_D–V_D curves of Ru 2 devices 1~5 fabricated by the AHPE method. The normalized SD at 0.1 V is 1.27%. These data were obtained from multiple independently fabricated device batches.

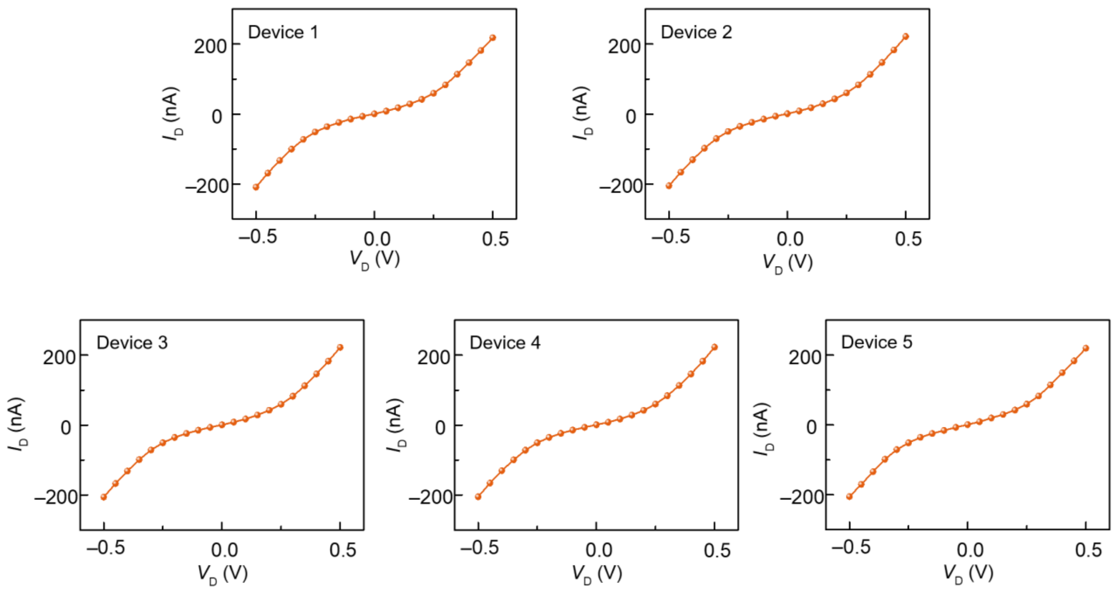

Figure S40. I_D–V_D curves of Ru 3 devices 1~5 fabricated by the AHPE method. The normalized SD at 0.1 V is 0.91%. These data were obtained from multiple independently fabricated device batches.

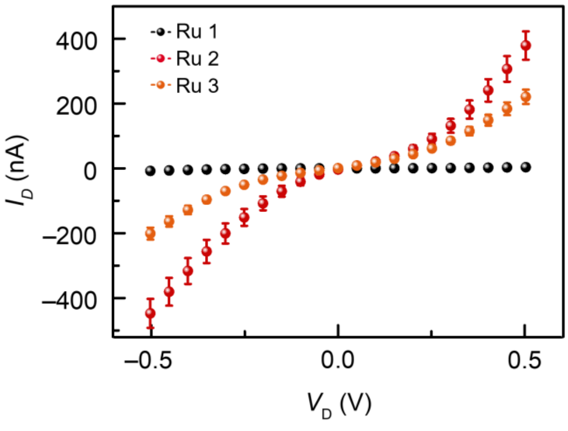

Figure S41. I_D–V_D curves of three organometallic Ru molecules measured in 5 devices with traditional configurations. The small dots denote the data means based on five devices and the bars indicate SDs from the mean values.

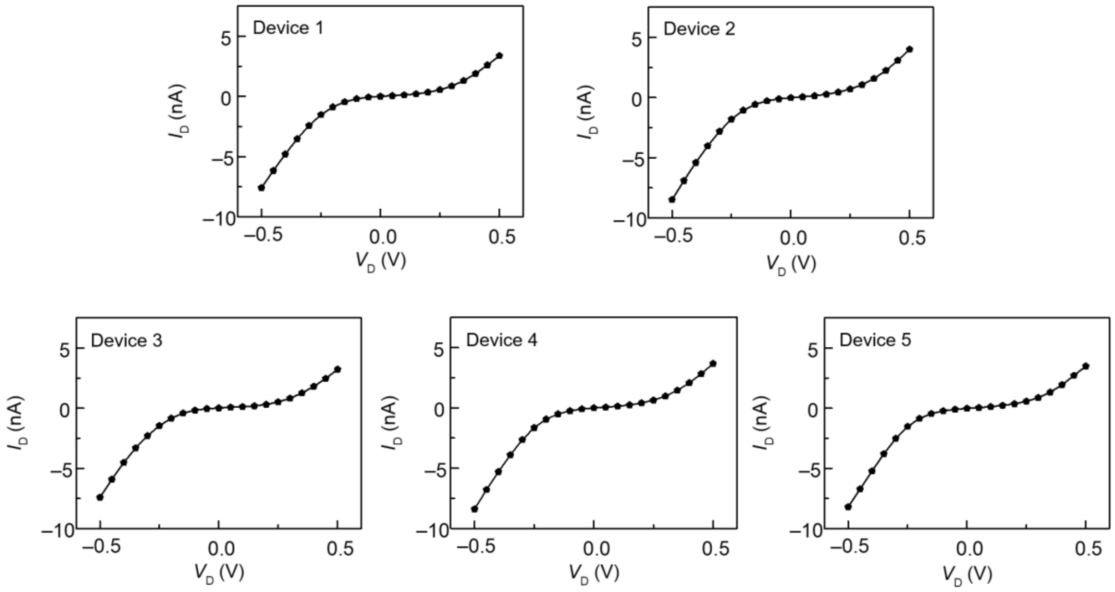

Figure S42. I_D–V_D curves of **Ru 1** devices 1~5 fabricated by the DLL method. The normalized SD at 0.1 V is 1.45%. These data were obtained from multiple independently fabricated device batches.

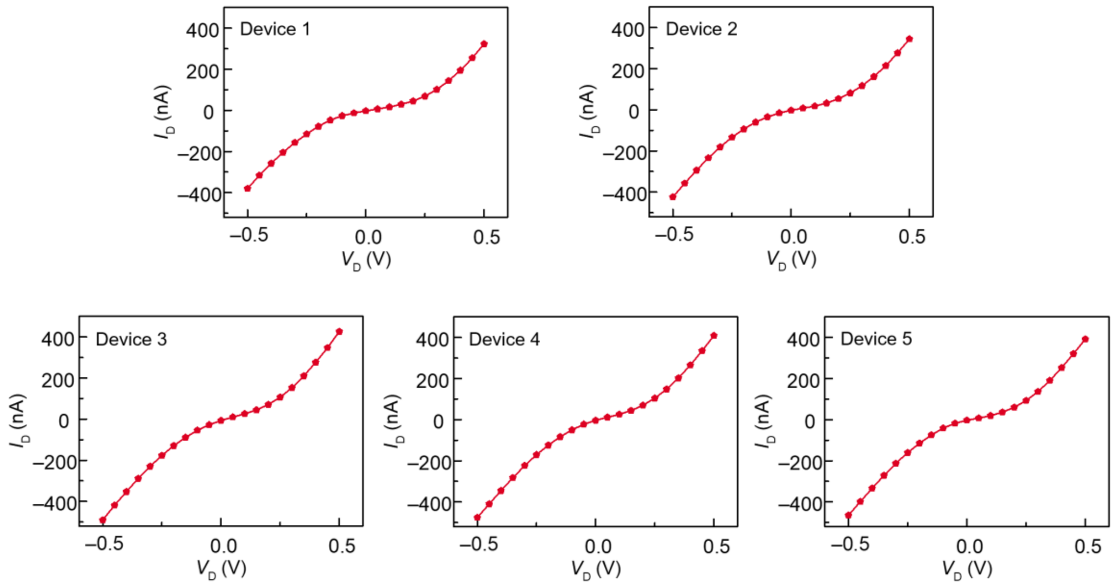

Figure S43. I_D–V_D curves of **Ru 2** devices 1~5 fabricated by the DLL method. The normalized SD at 0.1 V is 3.78%. These data were obtained from multiple independently fabricated device batches.

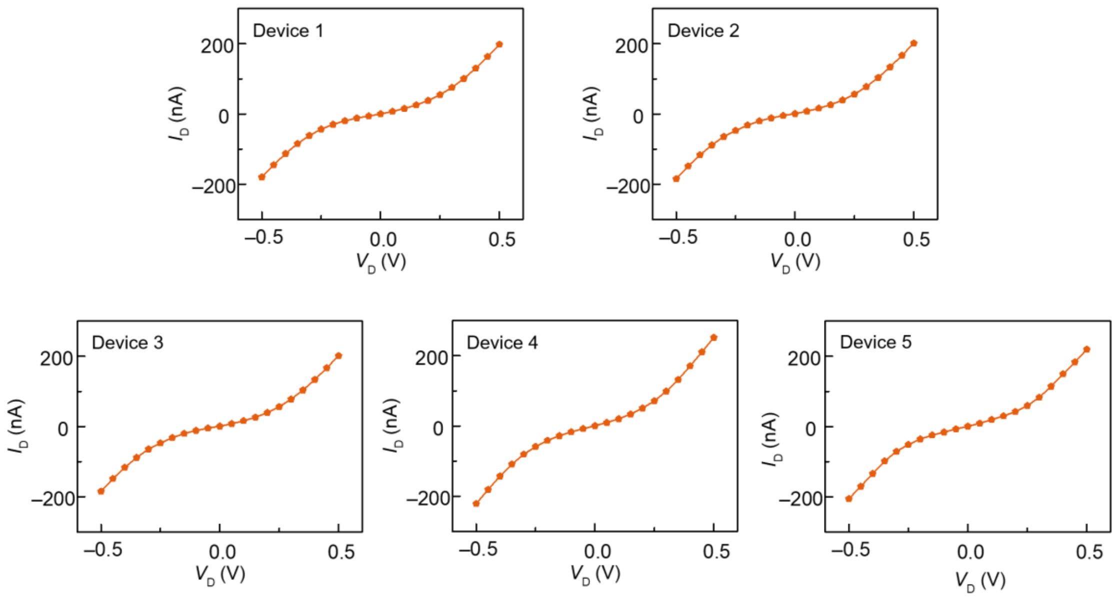

Figure S44. I_D–V_D curves of **Ru 3** devices 1~5 fabricated by the DLL method. The normalized SD at 0.1 V is 2.16%. These data were obtained from multiple independently fabricated device batches.

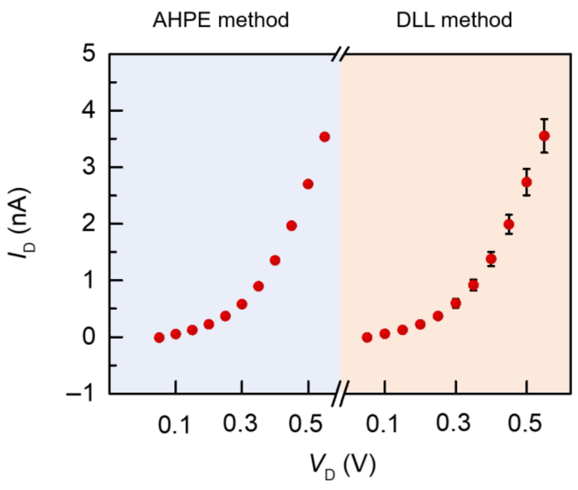

Figure S45. Current distribution of **Ru 1** devices at 300 K with precise and traditional configurations. The small red dots denote the data means based on five devices and the error bars indicate SDs from mean values.

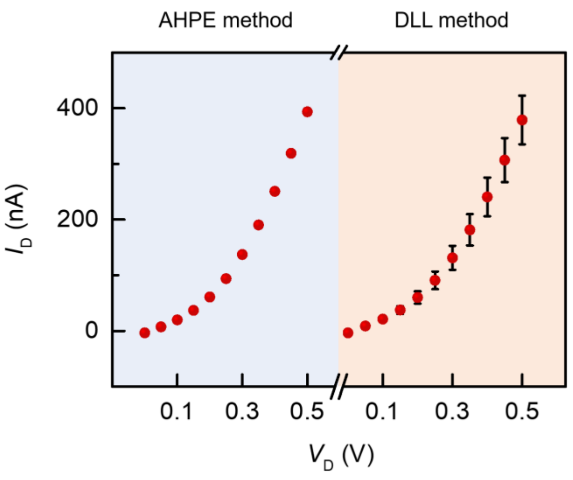

Figure S46. Current distribution of **Ru 2** devices at 300 K with precise and traditional configurations. The small red dots denote the data means based on five devices and the error bars indicate SDs from mean values.

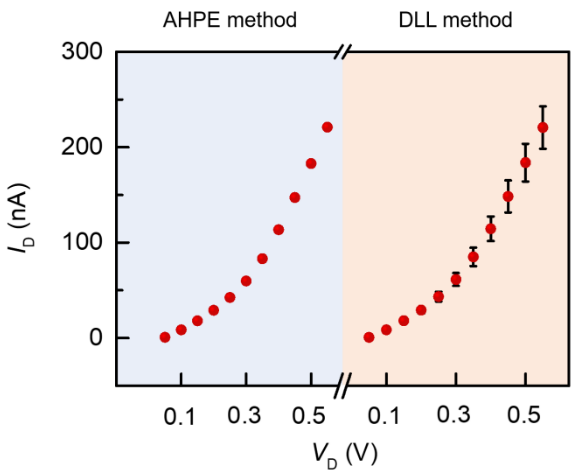

Figure S47. Current distribution of **Ru 3** devices at 300 K with precise and traditional configurations. The small red dots denote the data means based on five devices and the error bars indicate SDs from mean values.

Ru 1

Ru 2

Ru 3

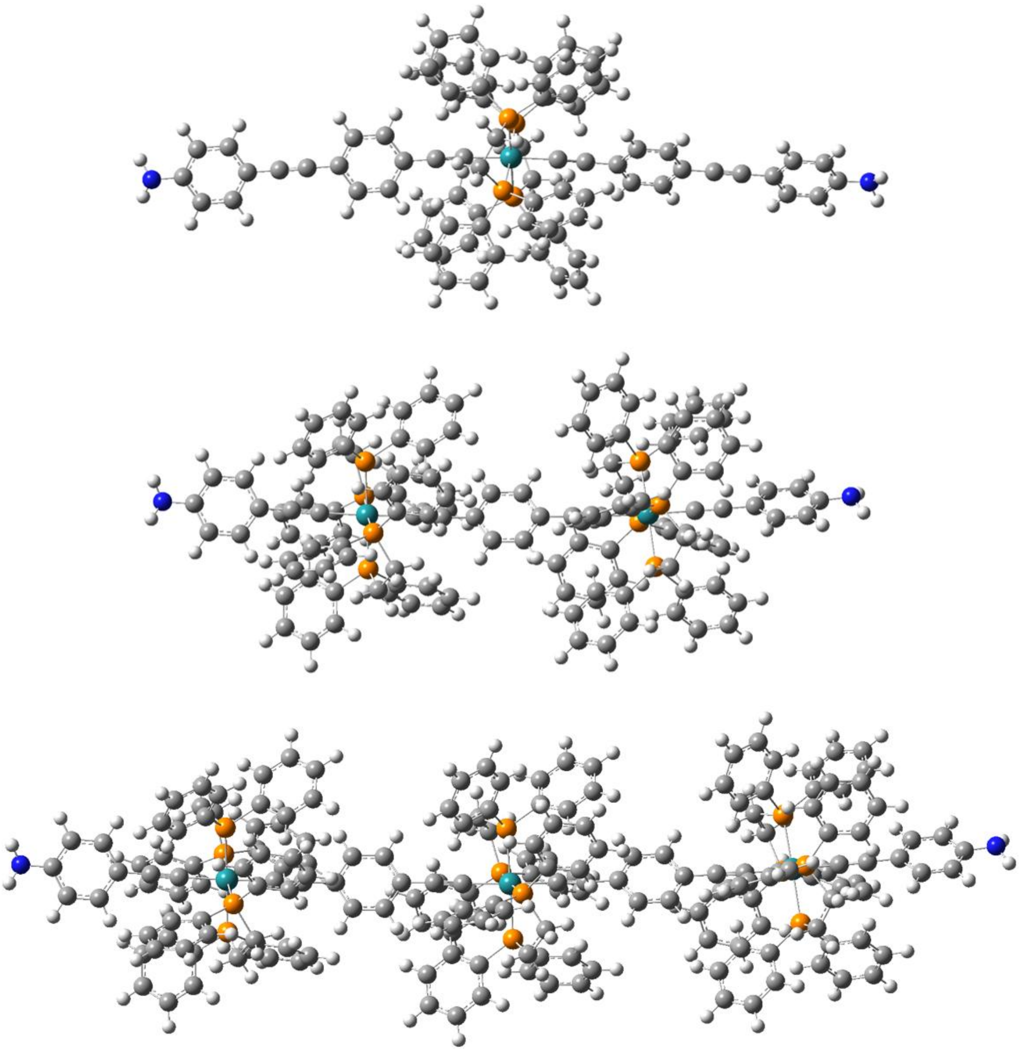

Figure S48. DFT calculated structures of three organometallic Ru molecules. The lengths of **Ru 1**, **Ru 2**, and **Ru 3** complexes are ~3.17 nm, ~3.02 nm, and ~4.25 nm, respectively.

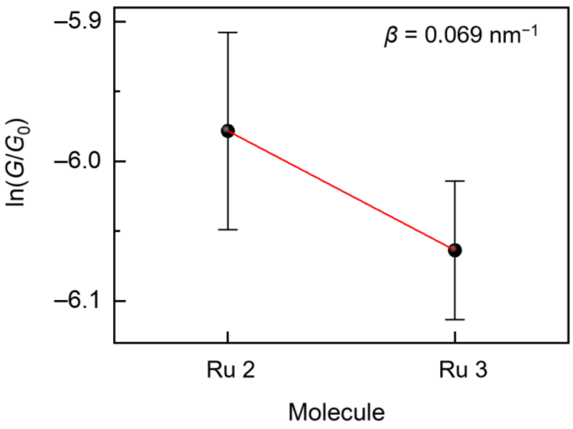

Figure S49. Dependence of conductance on the length of Ru 2 and Ru 3 devices. Error bars refer to SDs from mean values.

4.2 Scanning Tunneling Microscope Break Junctions

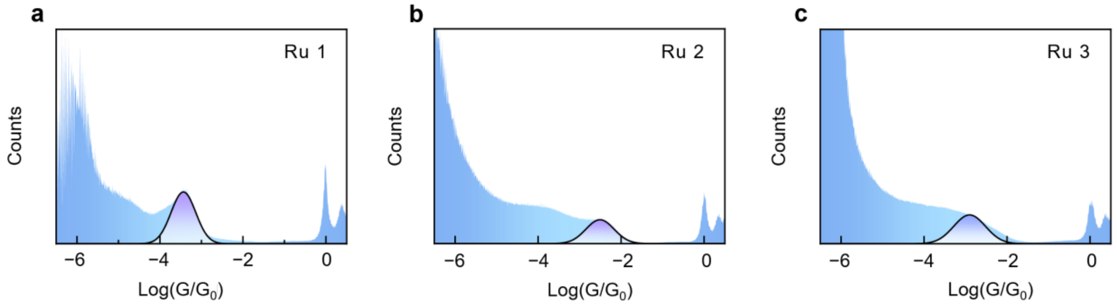

Figure S50. Electrical characteristics of organometallic Ru molecules based on scanning tunneling microscope break junctions (STM-BJs). 1D conductance histograms of system **Ru 1** (a), **Ru 2** (b), and **Ru 3** (c).

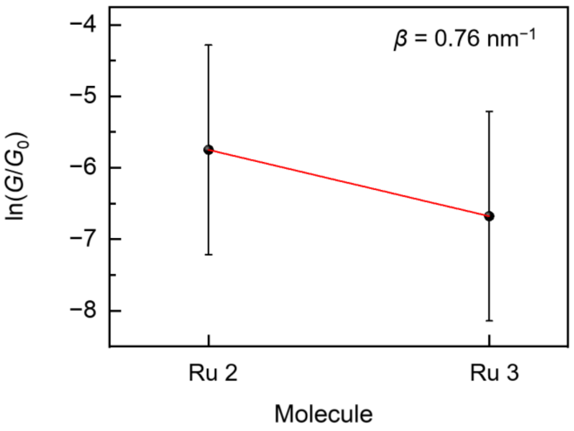

Figure S51. Dependence of conductance on length of **Ru 2** and **Ru 3** STM-BJs. Error bars refer to SDs from mean values.

4.3 Conductivities and Charge Transport Mechanisms of Ru Devices

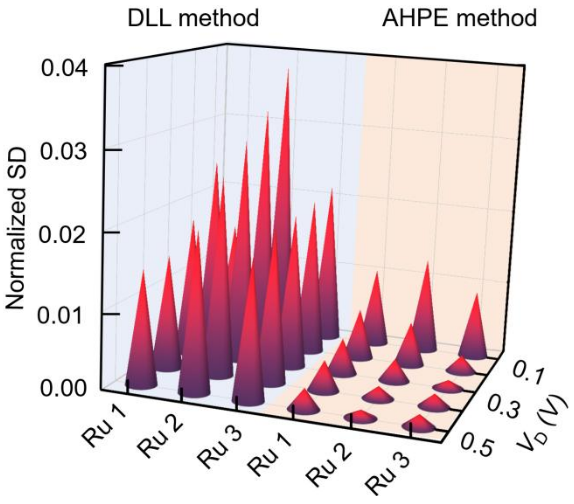

Figure S52. Normalized SDs of three organometallic Ru molecules measured in devices with precise and traditional configurations at different bias voltages. The conductance is normalized by $y_i = \frac{x_i}{\sum x_i}$, and the SD is derived from $\sigma = \frac{\Sigma(y_i - \mu)^2}{N}$, where the y_i is the normalized conductance, μ and σ are the mean and SD, respectively. N is the sample quantity.

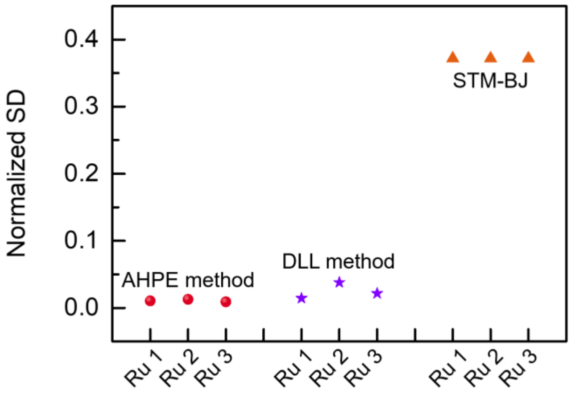

Figure S53. Normalized SDs of single-molecule devices using AHPE, DLL, and STM-BJ methods under a bias voltage of 0.1 V.

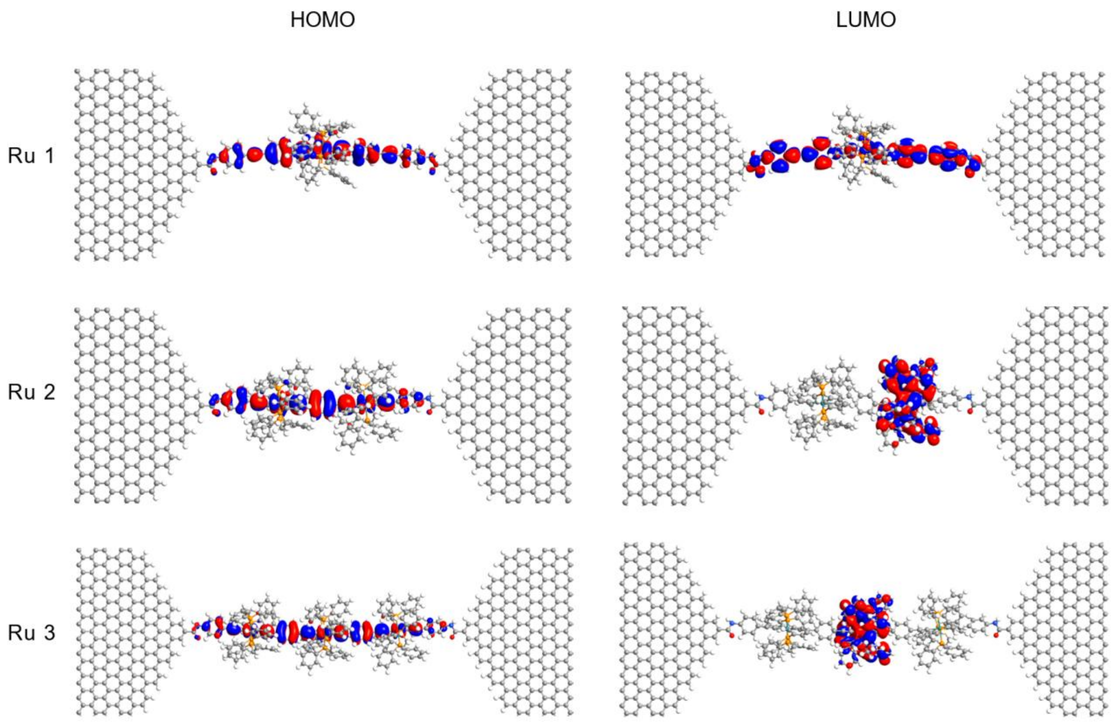

Figure S54. Molecular projected self-consistent Hamiltonian (MPSH) analysis of **Ru 1**, **Ru 2**, and **Ru 3** devices. The highest occupied molecular orbitals (HOMOs) drive the transmission in three organometallic Ru devices.

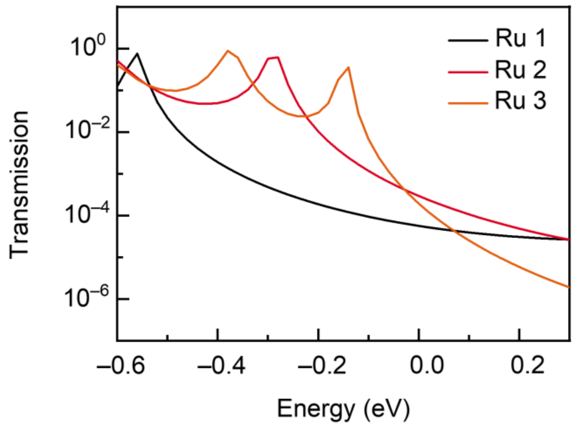

Figure S55. Theoretical transmission spectra of **Ru 1**, **Ru 2**, and **Ru 3** STM-BJs.

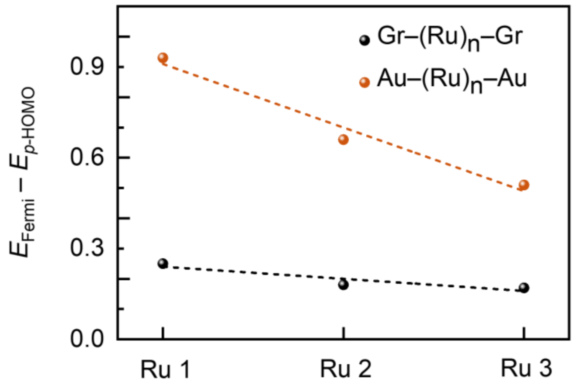

Figure S56. The distances between *p*-HOMO transmission peaks and the Fermi levels of **Ru 1**, **Ru 2**, and **Ru 3** devices. The distances between *p*-HOMO and the electrode Fermi levels for **Ru 1**, **Ru 2**, and **Ru 3** molecules in the graphene-based devices are smaller than those in STM-BJs.

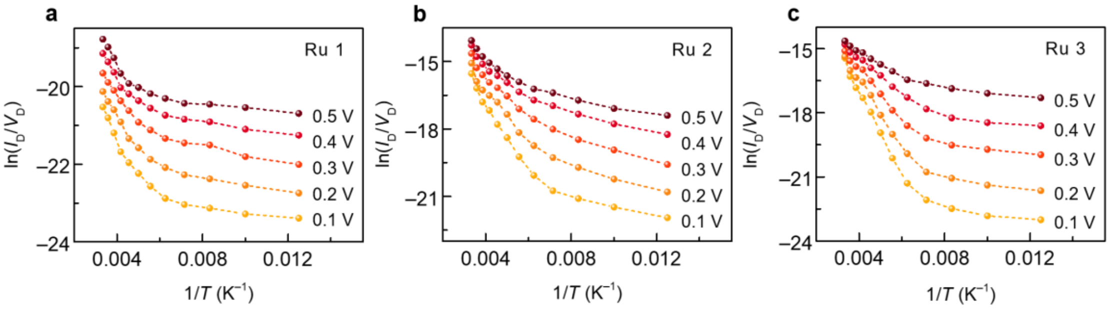

Figure S57. Plots of ln(I_D/V_D) vs 1/T under different biases for **Ru 1** (a), **Ru 2** (b), and **Ru 3** (c) devices.

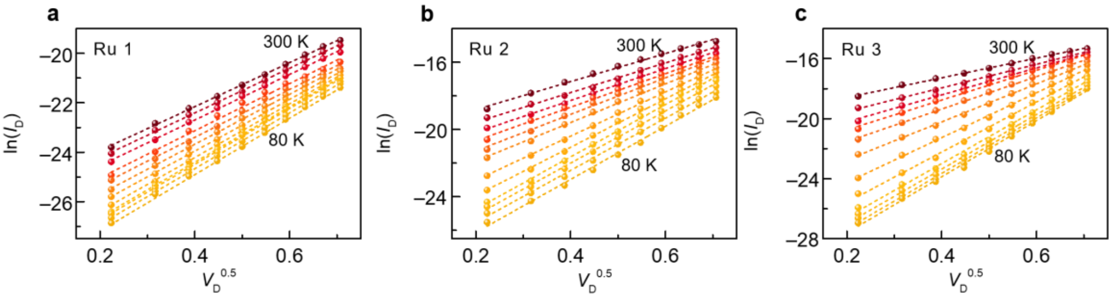

Figure S58. Plots of ln(I_D) vs $V_D^{0.5}$ at different temperatures for **Ru 1** (a), **Ru 2** (b), and **Ru 3** (c) devices.

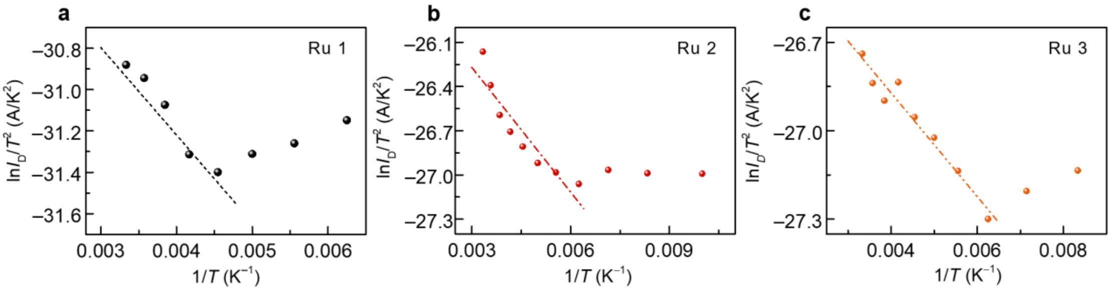

Figure S59. Plots of ln(I_D/T^2) vs 1/T for **Ru 1** (a), **Ru 2** (b), and **Ru 3** (c) devices.

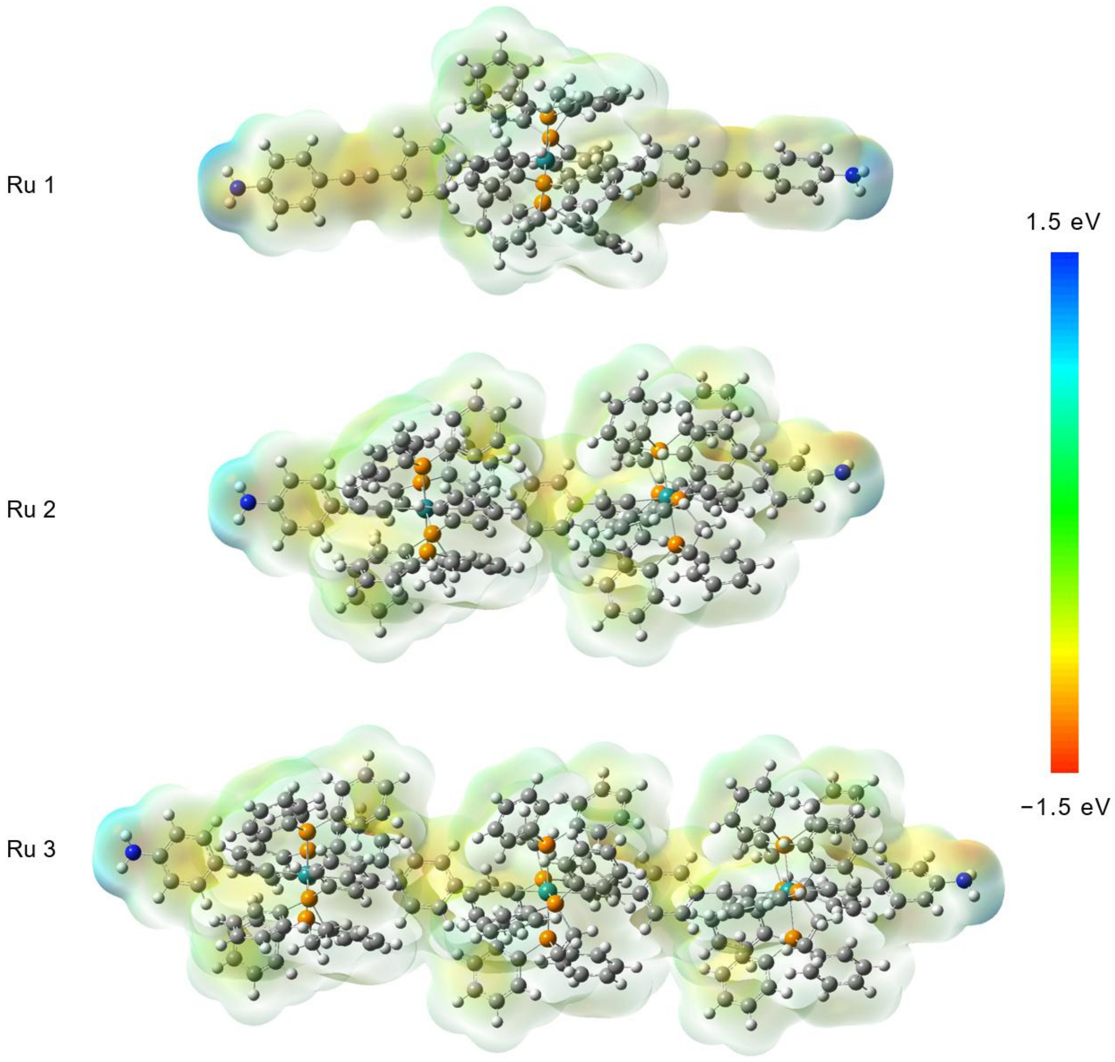

Figure S60. Calculated electrostatic-potential mapping of **Ru 1**, **Ru 2**, and **Ru 3**.

5. Cartesian Coordinates of the Optimized Molecular Geometries

All molecular geometry optimizations and orbital calculations were performed using the following methodology:

“#p opt b3lyp/6-31g(d) nosymm em=gd3bj”

Ru 1

C	2.08484	0.05430	0.04105
Ru	0.01480	0.14863	0.10588
C	-2.06005	0.14179	0.05685
P	0.19132	1.64513	-1.80153
P	0.11541	-1.58100	-1.53138
P	-0.08439	1.85536	1.82708
P	-0.33075	-1.32164	1.99013
C	-1.35500	-0.30419	3.17500
H	-2.32318	-0.12929	2.70088
H	-1.50968	-0.85400	4.10991
C	-0.62417	1.01424	3.43106
H	-1.24371	1.69860	4.01911
H	0.29155	0.82539	4.00100
C	1.01488	-1.83997	3.16941
C	2.30766	-1.30894	3.06369
C	0.72202	-2.68730	4.25538
C	3.28864	-1.62988	4.00865
H	2.55192	-0.65660	2.23281
C	1.69969	-3.00232	5.19744
H	-0.27182	-3.11187	4.36261
C	2.98929	-2.47598	5.07476
H	4.28702	-1.21461	3.90041
H	1.45470	-3.66036	6.02731
H	3.75213	-2.72567	5.80773
C	-1.30551	-2.88918	1.82866
C	-0.65493	-4.13173	1.73507
C	-2.70892	-2.86557	1.78577
C	-1.38585	-5.31473	1.61429
H	0.42746	-4.18399	1.77020
C	-3.43831	-4.05076	1.66944
H	-3.24099	-1.92338	1.82260
C	-2.78144	-5.27882	1.58544
H	-0.86031	-6.26403	1.55014
H	-4.52365	-4.00539	1.64292

H	-3.35129	-6.20050	1.50015
C	1.39531	2.80082	2.42641
C	2.54115	2.93440	1.63306
C	1.36467	3.43718	3.68157
C	3.63284	3.68482	2.08441
H	2.59148	2.43572	0.67451
C	2.45674	4.17518	4.13417
H	0.47957	3.37015	4.30771
C	3.59589	4.30200	3.33371
H	4.50949	3.77985	1.44978
H	2.41590	4.65644	5.10797
H	4.44630	4.88145	3.68399
C	-1.31246	3.24598	1.71422
C	-2.66527	3.04119	2.03001
C	-0.90648	4.53050	1.31924
C	-3.58071	4.09397	1.96271
H	-3.01545	2.05729	2.32116
C	-1.82498	5.57877	1.23867
H	0.13721	4.72088	1.09145
C	-3.16529	5.36546	1.56309
H	-4.61996	3.91671	2.22762
H	-1.48732	6.56590	0.93376
H	-3.87865	6.18367	1.51180
C	0.93847	-0.82609	-3.04063
H	2.00088	-0.82376	-2.77711
H	0.80751	-1.48215	-3.90826
C	0.46623	0.59166	-3.34941
H	-0.49055	0.57234	-3.87731
H	1.17937	1.10041	-4.00644
C	1.60871	2.84053	-1.89606
C	2.89855	2.40511	-2.23924
C	1.41890	4.20015	-1.60004
C	3.96748	3.30261	-2.28212
H	3.08249	1.35993	-2.45775
C	2.48792	5.09683	-1.64155
H	0.42818	4.56614	-1.35150
C	3.76632	4.65092	-1.98172
H	4.95756	2.94124	-2.54616
H	2.31773	6.14599	-1.41380
H	4.59840	5.34922	-2.01738
C	-1.17668	2.73186	-2.43315
C	-1.02134	3.37912	-3.67497

C	-2.36926	2.91485	-1.72532
C	-2.03718	4.17810	-4.19455
H	-0.09662	3.27110	-4.23500
C	-3.38769	3.71977	-2.24796
H	-2.51423	2.40635	-0.78216
C	-3.22688	4.35022	-3.47974
H	-1.89949	4.66880	-5.15473
H	-4.30847	3.84368	-1.68502
H	-4.02067	4.97275	-3.88474
C	1.21171	-3.07222	-1.37593
C	2.05713	-3.24490	-0.27300
C	1.28076	-4.00733	-2.42494
C	2.93920	-4.32745	-0.21012
H	2.05224	-2.51352	0.52391
C	2.15977	-5.08856	-2.36257
H	0.63535	-3.90217	-3.29199
C	2.99152	-5.25283	-1.25215
H	3.59032	-4.43576	0.65308
H	2.19450	-5.80137	-3.18246
H	3.67909	-6.09326	-1.20430
C	-1.45714	-2.31471	-2.18220
C	-2.33227	-1.53431	-2.95654
C	-1.83387	-3.63267	-1.88152
C	-3.51487	-2.07209	-3.46353
H	-2.11344	-0.49067	-3.14860
C	-3.02442	-4.16785	-2.37909
H	-1.19918	-4.25235	-1.25836
C	-3.86191	-3.39538	-3.18381
H	-4.17258	-1.44907	-4.06330
H	-3.29190	-5.19230	-2.13373
H	-4.78454	-3.81373	-3.57748
C	-3.29323	0.07938	0.03810
C	3.30890	-0.05729	-0.08324
C	4.72502	-0.17039	-0.18256
C	5.33355	-0.95603	-1.19015
C	5.58196	0.50051	0.72307
C	6.71366	-1.06029	-1.29154
H	4.69954	-1.49306	-1.88978
C	6.96198	0.39590	0.62435
H	5.14055	1.11082	1.50553
C	7.56185	-0.38558	-0.38711
H	7.15663	-1.66996	-2.07386

H	7.59889	0.92078	1.33076
C	-4.71503	-0.01429	-0.01015
C	-5.54198	1.10101	0.26312
C	-5.35775	-1.23452	-0.33024
C	-6.92581	1.00400	0.22511
H	-5.07542	2.05115	0.50463
C	-6.74158	-1.33339	-0.36686
H	-4.74662	-2.10141	-0.55826
C	-7.55993	-0.21736	-0.08836
H	-7.53869	1.87534	0.43827
H	-7.21168	-2.28045	-0.61668
C	-8.97769	-0.31970	-0.12470
C	-10.19239	-0.40834	-0.15479
C	8.97608	-0.48961	-0.49097
C	10.18790	-0.57709	-0.58091
C	-11.61031	-0.51185	-0.19099
C	-12.43134	0.60132	0.08607
C	-12.24578	-1.73095	-0.50720
C	-13.81543	0.50221	0.04738
H	-11.96610	1.55153	0.33052
C	-13.62969	-1.83226	-0.54645
H	-11.63560	-2.60210	-0.72605
C	-14.44139	-0.71703	-0.26994
H	-14.42586	1.37507	0.26901
H	-14.09485	-2.78530	-0.78930
C	11.60249	-0.67935	-0.68529
C	12.20597	-1.45852	-1.69461
C	12.45194	-0.00650	0.21790
C	13.58661	-1.56160	-1.79530
H	11.57345	-1.98758	-2.40110
C	13.83280	-0.10820	0.11898
H	12.01147	0.59824	1.00491
C	14.42686	-0.88818	-0.88993
H	14.02692	-2.16602	-2.58549
H	14.46565	0.42405	0.82597
N	-15.83283	-0.83201	-0.25324
H	-16.33682	0.03297	-0.40487
N	15.81525	-0.94519	-1.02519
H	16.16671	-1.76429	-1.50551
H	16.33737	-0.75725	-0.17831
H	-16.20804	-1.58511	-0.81648

Ru 2

C	-8.30145	0.07629	-0.26599
Ru	-6.22543	0.05987	-0.08871
C	-4.15583	0.06917	0.04430
P	-6.24838	-1.08152	-2.23485
P	-6.27349	2.02040	-1.48372
P	-6.21106	-1.90414	1.33502
P	-6.04948	1.22170	2.01542
C	-5.13806	0.02932	3.12749
H	-4.12458	-0.06234	2.73276
H	-5.08920	0.42330	4.14873
C	-5.84976	-1.32201	3.09619
H	-5.26708	-2.08302	3.62548
H	-6.82144	-1.24219	3.59511
C	-7.48485	1.62578	3.12958
C	-8.77966	1.18831	2.82341
C	-7.27036	2.29937	4.34726
C	-9.83801	1.42741	3.70721
H	-8.96002	0.67186	1.88767
C	-8.32536	2.53379	5.22708
H	-6.27623	2.65184	4.60710
C	-9.61566	2.09948	4.90755
H	-10.83603	1.08557	3.44543
H	-8.14076	3.05755	6.16171
H	-10.43870	2.28571	5.59298
C	-5.04410	2.77030	2.16975
C	-5.67015	4.02444	2.07004
C	-3.65358	2.73180	2.36802
C	-4.92913	5.20280	2.16812
H	-6.74307	4.08624	1.92143
C	-2.91457	3.91283	2.46977
H	-3.13161	1.78462	2.42191
C	-3.54876	5.15176	2.37018
H	-5.43635	6.15994	2.08341
H	-1.84017	3.85715	2.62430
H	-2.97250	6.06999	2.44907
C	-7.68480	-2.98769	1.65823
C	-8.76344	-3.04721	0.76777
C	-7.71176	-3.80661	2.80303
C	-9.84595	-3.89811	1.01841
H	-8.77260	-2.41591	-0.10977
C	-8.79366	-4.64785	3.05575
H	-6.87808	-3.80196	3.49903

C	-9.86662	-4.69590	2.16110
H	-10.67080	-3.92649	0.31208
H	-8.79578	-5.26987	3.94729
H	-10.70980	-5.35404	2.35542
C	-4.91437	-3.21298	1.08892
C	-3.58256	-2.98784	1.47308
C	-5.23823	-4.44339	0.49445
C	-2.60412	-3.96200	1.26622
H	-3.29558	-2.04175	1.91496
C	-4.25835	-5.41549	0.28393
H	-6.26278	-4.65172	0.20505
C	-2.93772	-5.17842	0.66833
H	-1.57983	-3.76041	1.56894
H	-4.53209	-6.36226	-0.17483
H	-2.17640	-5.93733	0.50724
C	-7.25064	1.48463	-2.97732
H	-8.25788	1.30641	-2.59230
H	-7.31169	2.28086	-3.72721
C	-6.65932	0.20186	-3.56361
H	-5.71495	0.41910	-4.07121
H	-7.33489	-0.24062	-4.30264
C	-7.51519	-2.39687	-2.57791
C	-8.84484	-2.06299	-2.88102
C	-7.16257	-3.75512	-2.52285
C	-9.79397	-3.05871	-3.12174
H	-9.15431	-1.02533	-2.91248
C	-8.11237	-4.74995	-2.76179
H	-6.13822	-4.03928	-2.30566
C	-9.43143	-4.40545	-3.06226
H	-10.81748	-2.77533	-3.35203
H	-7.81692	-5.79529	-2.71990
H	-10.16988	-5.18002	-3.25237
C	-4.76333	-1.88159	-3.00854
C	-4.77248	-2.18363	-4.38381
C	-3.63577	-2.22167	-2.25303
C	-3.67512	-2.79453	-4.98681
H	-5.64608	-1.95498	-4.98808
C	-2.53610	-2.83852	-2.85964
H	-3.60386	-1.97873	-1.19984
C	-2.55045	-3.12314	-4.22353
H	-3.69925	-3.01836	-6.05037
H	-1.66774	-3.08636	-2.25567

H	-1.69350	-3.59933	-4.69341
C	-7.17532	3.57600	-1.01110
C	-8.43394	3.48067	-0.39134
C	-6.65410	4.85109	-1.28161
C	-9.14716	4.63213	-0.05296
H	-8.85371	2.50325	-0.17777
C	-7.37014	6.00044	-0.93906
H	-5.68412	4.95497	-1.75422
C	-8.61746	5.89621	-0.32239
H	-10.11836	4.53583	0.42545
H	-6.94729	6.97827	-1.15595
H	-9.17313	6.79170	-0.05564
C	-4.70770	2.69267	-2.20895
C	-4.55516	2.95798	-3.58006
C	-3.63689	2.98860	-1.35069
C	-3.36439	3.49032	-4.07968
H	-5.36406	2.76254	-4.27594
C	-2.45102	3.53045	-1.84838
H	-3.72460	2.78732	-0.29149
C	-2.30790	3.77765	-3.21476
H	-3.26808	3.68275	-5.14521
H	-1.63508	3.74412	-1.16405
H	-1.38003	4.18985	-3.60279
C	-2.92731	0.09000	0.16754
C	-9.52451	0.10426	-0.43719
C	-10.94564	0.12095	-0.59455
C	-11.56870	0.85885	-1.62506
C	-11.79590	-0.59900	0.27475
C	-12.95078	0.87752	-1.78009
H	-10.95007	1.43441	-2.30867
C	-13.17811	-0.58100	0.12368
H	-11.35309	-1.18351	1.07595
C	-13.78294	0.15741	-0.90752
H	-13.39511	1.45578	-2.58826
H	-13.80251	-1.15113	0.80944
C	-1.50155	0.09484	0.20537
C	-0.77941	-0.11484	1.40364
C	-0.74261	0.30559	-0.96998
C	0.61059	-0.11962	1.42852
H	-1.32875	-0.27917	2.32828
C	0.64575	0.30313	-0.94484
H	-1.26826	0.47473	-1.90479

C	1.36790	0.08828	0.25267
H	1.13400	-0.29358	2.36486
H	1.19690	0.47629	-1.86596
C	4.03144	0.06383	0.28466
Ru	6.10796	0.02302	0.18130
C	8.17776	-0.00684	0.02109
P	5.80317	-1.98576	-1.15149
P	5.83632	1.10575	-1.94916
P	6.35899	-1.05454	2.33782
P	6.55721	2.02199	1.45236
C	7.62422	1.43629	2.86995
H	8.57644	1.11824	2.44124
H	7.80492	2.25694	3.57310
C	6.93062	0.26448	3.56301
H	7.58358	-0.18619	4.31753
H	6.02651	0.61492	4.07279
C	5.28389	2.97294	2.42281
C	3.97172	2.49880	2.54918
C	5.65188	4.13447	3.12816
C	3.04654	3.17401	3.35321
H	3.67015	1.60969	2.00734
C	4.72939	4.80385	3.93008
H	6.66202	4.52454	3.04390
C	3.42052	4.32514	4.04389
H	2.03204	2.79162	3.43085
H	5.03200	5.70041	4.46536
H	2.70092	4.84916	4.66798
C	7.55576	3.40929	0.73685
C	6.90678	4.50396	0.14058
C	8.96036	3.39426	0.75453
C	7.63991	5.55105	-0.41902
H	5.82310	4.54699	0.11844
C	9.69230	4.44590	0.19804
H	9.49476	2.55432	1.17967
C	9.03541	5.52712	-0.39080
H	7.11449	6.38411	-0.87781
H	10.77827	4.41210	0.22519
H	9.60608	6.34436	-0.82424
C	4.97106	-1.80795	3.31567
C	3.76658	-2.18664	2.71143
C	5.13473	-2.03938	4.69482
C	2.74777	-2.77732	3.46749

H	3.61210	-2.00079	1.65761
C	4.11653	-2.62049	5.44810
H	6.06765	-1.77866	5.18616
C	2.91684	-2.99223	4.83411
H	1.82234	-3.06330	2.97552
H	4.26190	-2.78839	6.51225
H	2.12293	-3.44849	5.42012
C	7.64635	-2.37635	2.55949
C	9.01014	-2.05045	2.63169
C	7.27972	-3.72970	2.63723
C	9.97738	-3.04708	2.77366
H	9.32852	-1.01827	2.55417
C	8.24764	-4.72642	2.77479
H	6.23186	-4.00895	2.60320
C	9.60030	-4.38917	2.84301
H	11.02666	-2.76789	2.82142
H	7.94040	-5.76741	2.83590
H	10.35340	-5.16515	2.95291
C	4.68017	-0.01499	-2.88721
H	3.73054	0.06127	-2.35117
H	4.51732	0.34003	-3.91073
C	5.20314	-1.45232	-2.86591
H	6.06883	-1.55001	-3.52752
H	4.43935	-2.15225	-3.21958
C	4.50996	-3.23333	-0.67615
C	3.14900	-2.99804	-0.92869
C	4.87483	-4.43176	-0.04142
C	2.18158	-3.93306	-0.55441
H	2.83123	-2.07630	-1.40085
C	3.90690	-5.36484	0.33446
H	5.92137	-4.64569	0.14893
C	2.55665	-5.11885	0.07949
H	1.13417	-3.72619	-0.75803
H	4.21169	-6.28841	0.82012
H	1.80388	-5.84793	0.36818
C	7.17373	-3.13974	-1.64012
C	6.96690	-4.05783	-2.68773
C	8.40791	-3.14014	-0.98057
C	7.97407	-4.94013	-3.07248
H	6.00921	-4.09653	-3.19952
C	9.41724	-4.02954	-1.36596
H	8.59282	-2.42891	-0.18727

C	9.20582	-4.92706	-2.41056
H	7.79639	-5.64032	-3.88492
H	10.36948	-4.00819	-0.84353
H	9.99238	-5.61527	-2.71003
C	4.98385	2.74703	-2.14344
C	3.85506	3.03592	-1.35644
C	5.41374	3.70264	-3.07773
C	3.18014	4.24938	-1.50216
H	3.50382	2.30733	-0.63341
C	4.73723	4.91653	-3.21818
H	6.28277	3.50889	-3.69598
C	3.61992	5.19587	-2.43036
H	2.31025	4.45226	-0.88291
H	5.08892	5.64362	-3.94596
H	3.09538	6.14181	-2.53961
C	7.28274	1.29060	-3.09132
C	7.26077	0.85866	-4.42779
C	8.44118	1.92566	-2.61631
C	8.36645	1.04621	-5.26078
H	6.37979	0.37941	-4.84113
C	9.54200	2.12233	-3.45095
H	8.48809	2.26115	-1.58913
C	9.51080	1.67898	-4.77436
H	8.32709	0.70048	-6.29064
H	10.42800	2.61153	-3.05660
H	10.37163	1.82536	-5.42173
C	9.41002	0.01329	-0.04313
C	2.79583	0.07665	0.26555
C	10.83122	-0.01279	-0.18351
C	11.69664	0.33448	0.87740
C	11.44167	-0.39581	-1.39967
C	13.08092	0.30234	0.73726
H	11.27014	0.63236	1.83236
C	12.82404	-0.42927	-1.54312
H	10.80705	-0.66982	-2.23655
C	13.67130	-0.08015	-0.47776
H	13.71548	0.58195	1.57650
H	13.25922	-0.72560	-2.49605
N	15.06627	-0.05380	-0.64358
H	15.58656	-0.18044	0.21692
H	15.40756	-0.68344	-1.36071

Ru 3

C	-14.45905	0.04118	-0.45304
Ru	-12.38570	0.04900	-0.24870
C	-10.31759	0.08339	-0.08832
P	-12.36453	-1.07541	-2.40284
P	-12.43949	2.01984	-1.62823
P	-12.37104	-1.92602	1.16120
P	-12.24950	1.19575	1.86662
C	-11.33648	0.00482	2.97867
H	-10.31840	-0.07315	2.59304
H	-11.30177	0.39126	4.00338
C	-12.03362	-1.35351	2.93025
H	-11.44833	-2.11230	3.45983
H	-13.01098	-1.28725	3.41993
C	-13.70365	1.57045	2.96665
C	-14.98838	1.11743	2.64106
C	-13.51324	2.23504	4.19328
C	-16.06020	1.33221	3.51476
H	-15.15065	0.60753	1.69844
C	-14.58163	2.44508	5.06308
H	-12.52752	2.59933	4.46839
C	-15.86161	1.99513	4.72433
H	-17.05001	0.97857	3.23794
H	-14.41546	2.96198	6.00495
H	-16.69512	2.16223	5.40199
C	-11.26611	2.75536	2.04802
C	-11.90646	4.00206	1.94534
C	-9.87893	2.73284	2.27075
C	-11.18221	5.18895	2.06348
H	-12.97731	4.05099	1.77798
C	-9.15688	3.92246	2.39264
H	-9.34602	1.79198	2.32906
C	-9.80502	5.15386	2.28925
H	-11.69990	6.14020	1.97593
H	-8.08481	3.87947	2.56607
H	-9.24192	6.07875	2.38377
C	-13.84037	-3.02242	1.45999
C	-14.90178	-3.09394	0.54981
C	-13.87958	-3.84170	2.60420
C	-15.97925	-3.95677	0.78052
H	-14.90194	-2.46296	-0.32796
C	-14.95657	-4.69493	2.83700
H	-13.05921	-3.82825	3.31569

C	-16.01222	-4.75484	1.92275
H	-16.79072	-3.99382	0.05926
H	-14.96818	-5.31699	3.72844
H	-16.85155	-5.42223	2.10156
C	-11.06272	-3.22467	0.92197
C	-9.73630	-2.98959	1.31837
C	-11.37144	-4.45682	0.32307
C	-8.74841	-3.95562	1.11927
H	-9.46114	-2.04162	1.76362
C	-10.38189	-5.42084	0.12031
H	-12.39157	-4.67311	0.02414
C	-9.06674	-5.17397	0.51709
H	-7.72852	-3.74606	1.43128
H	-10.64404	-6.36919	-0.34196
H	-8.29782	-5.92653	0.36247
C	-13.38655	1.48343	-3.14060
H	-14.39694	1.28850	-2.77200
H	-13.44769	2.28462	-3.88516
C	-12.76914	0.21335	-3.72798
H	-11.81932	0.44698	-4.21799
H	-13.42666	-0.23119	-4.48192
C	-13.61203	-2.40156	-2.77431
C	-14.93992	-2.07937	-3.09700
C	-13.24662	-3.75646	-2.72045
C	-15.87515	-3.08322	-3.35758
H	-15.25914	-1.04466	-3.12876
C	-14.18248	-4.75945	-2.97946
H	-12.22312	-4.03155	-2.48809
C	-15.50005	-4.42657	-3.29896
H	-16.89776	-2.80881	-3.60224
H	-13.87724	-5.80199	-2.93814
H	-16.22762	-5.20749	-3.50440
C	-10.85971	-1.85534	-3.15816
C	-10.84272	-2.14692	-4.53556
C	-9.74358	-2.19450	-2.38541
C	-9.73167	-2.74780	-5.12344
H	-11.70659	-1.91841	-5.15370
C	-8.63106	-2.80286	-2.97668
H	-9.73044	-1.95799	-1.33033
C	-8.61961	-3.07789	-4.34254
H	-9.73562	-2.96353	-6.18893
H	-7.77423	-3.05445	-2.35769

H	-7.75261	-3.54754	-4.80039
C	-13.37039	3.55836	-1.15529
C	-14.63617	3.43931	-0.55454
C	-12.86417	4.84328	-1.40732
C	-15.37088	4.57720	-0.21615
H	-15.04457	2.45396	-0.35595
C	-13.60173	5.97897	-1.06486
H	-11.88941	4.96544	-1.86538
C	-14.85591	5.85116	-0.46679
H	-16.34722	4.46251	0.24749
H	-13.19030	6.96476	-1.26722
H	-15.42824	6.73610	-0.20006
C	-10.87377	2.72326	-2.32348
C	-10.70647	3.00599	-3.68940
C	-9.82126	3.02972	-1.44654
C	-9.51959	3.56766	-4.16564
H	-11.50128	2.80237	-4.39905
C	-8.63963	3.60128	-1.92072
H	-9.92018	2.81419	-0.39114
C	-8.48211	3.86722	-3.28205
H	-9.41180	3.77400	-5.22746
H	-7.83812	3.82376	-1.22231
H	-7.55789	4.30392	-3.65157
C	-9.09143	0.12176	0.05292
C	-15.68005	0.05267	-0.64004
C	-17.09915	0.04939	-0.81545
C	-17.71963	0.77856	-1.85370
C	-17.94993	-0.68327	0.04267
C	-19.09972	0.77688	-2.02665
H	-17.10073	1.36348	-2.52908
C	-19.33017	-0.68560	-0.12630
H	-17.50894	-1.26180	0.84917
C	-19.93240	0.04421	-1.16511
H	-19.54202	1.34890	-2.84035
H	-19.95491	-1.26516	0.55119
C	-7.66721	0.15073	0.12808
C	-6.97430	-0.11944	1.33159
C	-6.88004	0.44711	-1.00957
C	-5.58588	-0.10419	1.39552
H	-7.54567	-0.34917	2.22840
C	-5.49282	0.46918	-0.94393
H	-7.38241	0.66436	-1.94717

C	-4.79996	0.18947	0.25716
H	-5.08518	-0.33360	2.33229
H	-4.91954	0.70984	-1.83610
C	-2.14011	0.16018	0.39031
Ru	-0.06139	0.08469	0.39932
C	2.01172	0.02519	0.35605
P	-0.30721	-1.56906	-1.35950
P	-0.16833	1.61813	-1.45059
P	0.00783	-1.44508	2.28119
P	0.34276	1.73567	2.11296
C	1.30989	0.81407	3.41974
H	2.27924	0.56509	2.98353
H	1.46659	1.45163	4.29697
C	0.54900	-0.45559	3.79656
H	1.14948	-1.08942	4.45720
H	-0.36926	-0.19313	4.33263
C	-0.97245	2.48610	3.19568
C	-2.30337	2.05578	3.12013
C	-0.62601	3.43730	4.17430
C	-3.26723	2.56966	3.99497
H	-2.58808	1.32890	2.36804
C	-1.58711	3.94559	5.04609
H	0.39786	3.79192	4.25048
C	-2.91401	3.51341	4.95756
H	-4.29535	2.22649	3.91302
H	-1.30054	4.68076	5.79395
H	-3.66362	3.91236	5.63639
C	1.40668	3.21755	1.78914
C	0.81484	4.43161	1.40055
C	2.80610	3.16083	1.89886
C	1.59849	5.55453	1.13263
H	-0.26370	4.50601	1.31084
C	3.58821	4.28802	1.63513
H	3.30006	2.23541	2.16679
C	2.98798	5.48783	1.25117
H	1.11740	6.48031	0.82929
H	4.66894	4.22006	1.72820
H	3.59773	6.36376	1.04529
C	-1.47124	-2.32107	2.98478
C	-2.61621	-2.56222	2.21586
C	-1.43806	-2.79728	4.30934
C	-3.70561	-3.25099	2.76050

H	-2.67098	-2.19649	1.20013
C	-2.52577	-3.47876	4.85217
H	-0.55385	-2.65006	4.92230
C	-3.66636	-3.70675	4.07697
H	-4.58416	-3.42087	2.14450
H	-2.48061	-3.83539	5.87808
H	-4.51536	-4.23851	4.49920
C	1.22897	-2.84690	2.28337
C	2.59764	-2.60550	2.48396
C	0.80932	-4.16951	2.06763
C	3.51857	-3.65456	2.46972
H	2.95594	-1.59372	2.62759
C	1.73161	-5.21771	2.04859
H	-0.24411	-4.38702	1.92613
C	3.08928	-4.96463	2.25011
H	4.57283	-3.43967	2.62369
H	1.38429	-6.23458	1.88385
H	3.80593	-5.78177	2.23935
C	-1.26970	0.76635	-2.68961
H	-2.25569	0.75452	-2.21687
H	-1.35145	1.34251	-3.61788
C	-0.77180	-0.65605	-2.95066
H	0.13827	-0.63335	-3.55710
H	-1.51772	-1.23845	-3.50076
C	-1.64922	-2.85096	-1.26464
C	-2.98274	-2.52411	-1.55783
C	-1.35224	-4.17017	-0.88573
C	-3.98979	-3.48766	-1.47310
H	-3.24939	-1.51159	-1.83554
C	-2.35981	-5.13255	-0.79933
H	-0.32718	-4.45420	-0.67131
C	-3.68231	-4.79498	-1.09256
H	-5.01450	-3.20658	-1.70166
H	-2.10698	-6.14943	-0.50983
H	-4.46574	-5.54584	-1.02969
C	1.07757	-2.63183	-1.99246
C	0.94455	-3.28026	-3.23539
C	2.24590	-2.83603	-1.25043
C	1.96107	-4.09670	-3.72643
H	0.03664	-3.16189	-3.82036
C	3.26336	-3.66082	-1.74243
H	2.37431	-2.33127	-0.30313

C	3.12687	-4.28890	-2.97867
H	1.84129	-4.58720	-4.68920
H	4.16319	-3.80171	-1.15004
H	3.92023	-4.92644	-3.36081
C	-0.98689	3.28271	-1.32731
C	-2.15200	3.41998	-0.55347
C	-0.49565	4.40723	-2.00940
C	-2.80340	4.65152	-0.46379
H	-2.54786	2.55864	-0.02642
C	-1.14873	5.63833	-1.91460
H	0.40277	4.33053	-2.61147
C	-2.30263	5.76612	-1.14026
H	-3.70235	4.73620	0.14124
H	-0.75021	6.49775	-2.44802
H	-2.80857	6.72547	-1.06603
C	1.35614	2.01760	-2.42336
C	1.42993	1.88000	-3.81924
C	2.47662	2.52142	-1.74404
C	2.59135	2.22679	-4.51397
H	0.58186	1.51031	-4.38557
C	3.63239	2.87913	-2.43903
H	2.45044	2.62873	-0.66794
C	3.69622	2.72851	-3.82560
H	2.62607	2.10829	-5.59398
H	4.48742	3.26151	-1.88914
H	4.60034	2.99897	-4.36490
C	3.24640	0.02822	0.37100
C	-3.37325	0.18975	0.31363
C	4.67180	0.01488	0.31825
C	5.46464	0.01299	1.49012
C	5.35973	0.00698	-0.91800
C	6.85344	0.00720	1.43403
H	4.97132	0.01535	2.45974
C	6.74734	0.00882	-0.97401
H	4.78042	0.00946	-1.83603
C	7.53981	0.01002	0.19761
H	7.43192	-0.00667	2.35394
H	7.24277	0.01708	-1.94197
N	-21.31997	-0.00781	-1.37341
H	-21.69676	0.80116	-1.85369
H	-21.85653	-0.20764	-0.53725
C	10.20173	0.02053	0.11111

Ru	12.27584	-0.00148	-0.03593
C	14.34274	-0.02256	-0.22873
P	11.96715	-2.01774	-1.35598
P	11.96095	1.07031	-2.16485
P	12.56460	-1.06377	2.12448
P	12.73679	2.00791	1.21646
C	13.83279	1.43396	2.61636
H	14.77648	1.11427	2.17029
H	14.02628	2.25914	3.31071
C	13.15414	0.26588	3.32976
H	13.82115	-0.17808	4.07589
H	12.25855	0.61844	3.85290
C	11.47840	2.96083	2.20448
C	10.16284	2.49857	2.33712
C	11.86121	4.11703	2.91083
C	9.24840	3.18012	3.14815
H	9.85141	1.61239	1.79630
C	10.94978	4.79232	3.72034
H	12.87412	4.49876	2.82067
C	9.63707	4.32555	3.84011
H	8.23065	2.80721	3.22985
H	11.26367	5.68450	4.25644
H	8.92590	4.85453	4.46965
C	13.71427	3.39744	0.47643
C	13.04798	4.48721	-0.10964
C	15.11901	3.39015	0.46729
C	13.76453	5.53733	-0.68476
H	11.96379	4.52416	-0.11165
C	15.83438	4.44488	-0.10472
H	15.66614	2.55401	0.88368
C	15.16043	5.52141	-0.68275
H	13.22589	6.36660	-1.13506
H	16.92085	4.41721	-0.09799
H	15.71817	6.34117	-1.12810
C	11.19224	-1.81129	3.12795
C	9.98864	-2.21692	2.53942
C	11.36678	-2.01127	4.51061
C	8.97982	-2.79949	3.31459
H	9.82636	-2.05893	1.48237
C	10.35918	-2.58639	5.28257
H	12.29948	-1.72984	4.99069
C	9.15915	-2.98231	4.68454

H	8.05375	-3.10309	2.83459
H	10.51281	-2.72984	6.34914
H	8.37293	-3.43210	5.28574
C	13.85796	-2.38113	2.33672
C	15.22205	-2.05219	2.38705
C	13.49496	-3.73449	2.42891
C	16.19307	-3.04614	2.52186
H	15.53741	-1.01996	2.29836
C	14.46665	-4.72855	2.55891
H	12.44719	-4.01566	2.41203
C	15.81951	-4.38843	2.60546
H	17.24243	-2.76483	2.55297
H	14.16229	-5.76965	2.63146
H	16.57558	-5.16230	2.70972
C	10.79648	-0.06275	-3.07705
H	9.85650	0.00729	-2.52316
H	10.61049	0.28726	-4.09836
C	11.33414	-1.49470	-3.06177
H	12.18980	-1.58387	-3.73752
H	10.57215	-2.20326	-3.40174
C	10.69353	-3.27640	-0.85838
C	9.32710	-3.05232	-1.09016
C	11.07746	-4.47135	-0.22839
C	8.37253	-3.99415	-0.70099
H	8.99541	-2.13340	-1.55800
C	10.12237	-5.41150	0.16265
H	12.12840	-4.67706	-0.05377
C	8.76641	-5.17642	-0.07200
H	7.32099	-3.79415	-0.88933
H	10.44169	-6.33234	0.64415
H	8.02368	-5.91089	0.22869
C	13.34179	-3.15950	-1.86079
C	13.13531	-4.07187	-2.91333
C	14.57805	-3.15905	-1.20512
C	14.14502	-4.94736	-3.30709
H	12.17613	-4.11166	-3.42218
C	15.58985	-4.04165	-1.59937
H	14.76242	-2.45239	-0.40771
C	15.37887	-4.93320	-2.64920
H	13.96763	-5.64307	-4.12341
H	16.54369	-4.01960	-1.07992
H	16.16742	-5.61598	-2.95575

C	11.09291	2.70383	-2.34972
C	9.96649	2.97837	-1.55429
C	11.50761	3.66693	-3.28311
C	9.27890	4.18570	-1.69086
H	9.62761	2.24347	-0.83160
C	10.81824	4.87461	-3.41445
H	12.37435	3.48378	-3.90779
C	9.70340	5.13992	-2.61832
H	8.41109	4.37791	-1.06530
H	11.15782	5.60787	-4.14177
H	9.16875	6.08092	-2.72078
C	13.38531	1.26050	-3.33381
C	13.33819	0.83148	-4.67062
C	14.55132	1.89717	-2.88006
C	14.42694	1.02336	-5.52455
H	12.45036	0.35110	-5.06769
C	15.63530	2.09808	-3.73559
H	14.61752	2.23041	-1.85324
C	15.57922	1.65761	-5.05909
H	14.36833	0.67980	-6.55422
H	16.52773	2.58847	-3.35756
H	16.42691	1.80734	-5.72284
C	15.57381	0.00280	-0.31135
C	8.96605	0.01477	0.13388
C	16.99306	-0.01415	-0.47167
C	17.87050	0.34421	0.57561
C	17.58948	-0.39724	-1.69476
C	19.25288	0.32274	0.41622
H	17.45500	0.64259	1.53525
C	18.96992	-0.42004	-1.85746
H	16.94557	-0.67978	-2.52167
C	19.82922	-0.05970	-0.80555
H	19.89682	0.61083	1.24537
H	19.39414	-0.71666	-2.81522
N	21.22141	-0.02242	-0.99128
H	21.75499	-0.14080	-0.13778
H	21.55782	-0.65226	-1.71053

6. References

[1] N. Xin, C. Hu, H. Al Sabea, M. Zhang, C. Zhou, L. Meng, C. Jia, Y. Gong, Y. Li, G. Ke, X. He, P. Selvanathan, L. Norel, M. A. Ratner, Z. Liu, S. Xiao, S. Rigaut, H. Guo, X. Guo, Tunable symmetry-breaking-induced dual functions in stable and photoswitched single-molecule junctions. *J. Am. Chem. Soc.* **2021**, *143*, 20811.

[2] A. Klein, O. Lavastre, J. Fiedler, Role of the bridging arylethynyl ligand in bi- and trinuclear ruthenium and iron complexes. *Organometallics* **2006,** *25*, 635

[3] D. Andrae, U. Häußermann, M. Dolg, H. Stoll, H. Preuß, Energy-adjusted ab initio pseudopotentials for the second and third row transition elements. *Theor. Chim. Acta* **1990**, *77*, 123.

[4] A. D. Becke, Density-functional thermochemistry. I. The effect of the exchange-only gradient correction. *J. Chem. Phys.* **1992**, *96*, 2155.

[5] M. Brandbyge, J. L. Mozos, P. Ordejón, J. Taylor, K. Stokbro, Density-functional method for nonequilibrium electron transport. *Phys. Rev. B* **2002**, *65*, 165401.

[6] B. Ma, P. Wang, S. Ren, C. Jia, X. Guo, Versatile optical determination of two-dimensional atomic crystal layers. *Carbon* **2016**, *109*, 384.

[7] B. Ma, S. Ren, P. Wang, C. Jia, X. Guo, Precise control of graphene etching by remote hydrogen plasma. *Nano Res.* **2019**, *12*, 137.
